# The DNA replication checkpoint limits Okazaki fragment accumulation to protect and restart stalled forks

**DOI:** 10.1016/j.molcel.2025.06.001

**Published:** 2025-06-26

**Authors:** Berta Canal, Agostina P. Bertolin, Giselle C. Lee, Lucy S. Drury, Masashi Minamino, John F.X. Diffley

**Affiliations:** 1 Chromosome Replication Laboratory, The Francis Crick Institute, London NW1 1AT, UK; 2 Chromosome Segregation Laboratory, The Francis Crick Institute, London NW1 1AT, UK

## Abstract

Understanding how DNA replication forks stall and restart and how the DNA replication checkpoint prevents irreversible fork collapse in molecular detail are crucial for understanding how cells maintain stable genomes and how they prevent the genetic instability that drives cancer. Here, we describe the reconstitution of fork stalling and restart with purified budding yeast proteins. After nucleotide depletion, leading-strand DNA synthesis quickly stops but CMG helicase continues to unwind, and Okazaki fragments continue to initiate on the lagging strand. Incomplete Okazaki fragments sequester PCNA, RFC, and DNA polymerases **δ** and **ε**, which prevents normal DNA synthesis restart and exposes nascent DNA to nuclease attack. The DNA replication checkpoint restrains fork progression, which limits this sequestration, protecting stalled forks from collapse and ensuring restart.

## Graphical abstract

**Graphical abstract:**
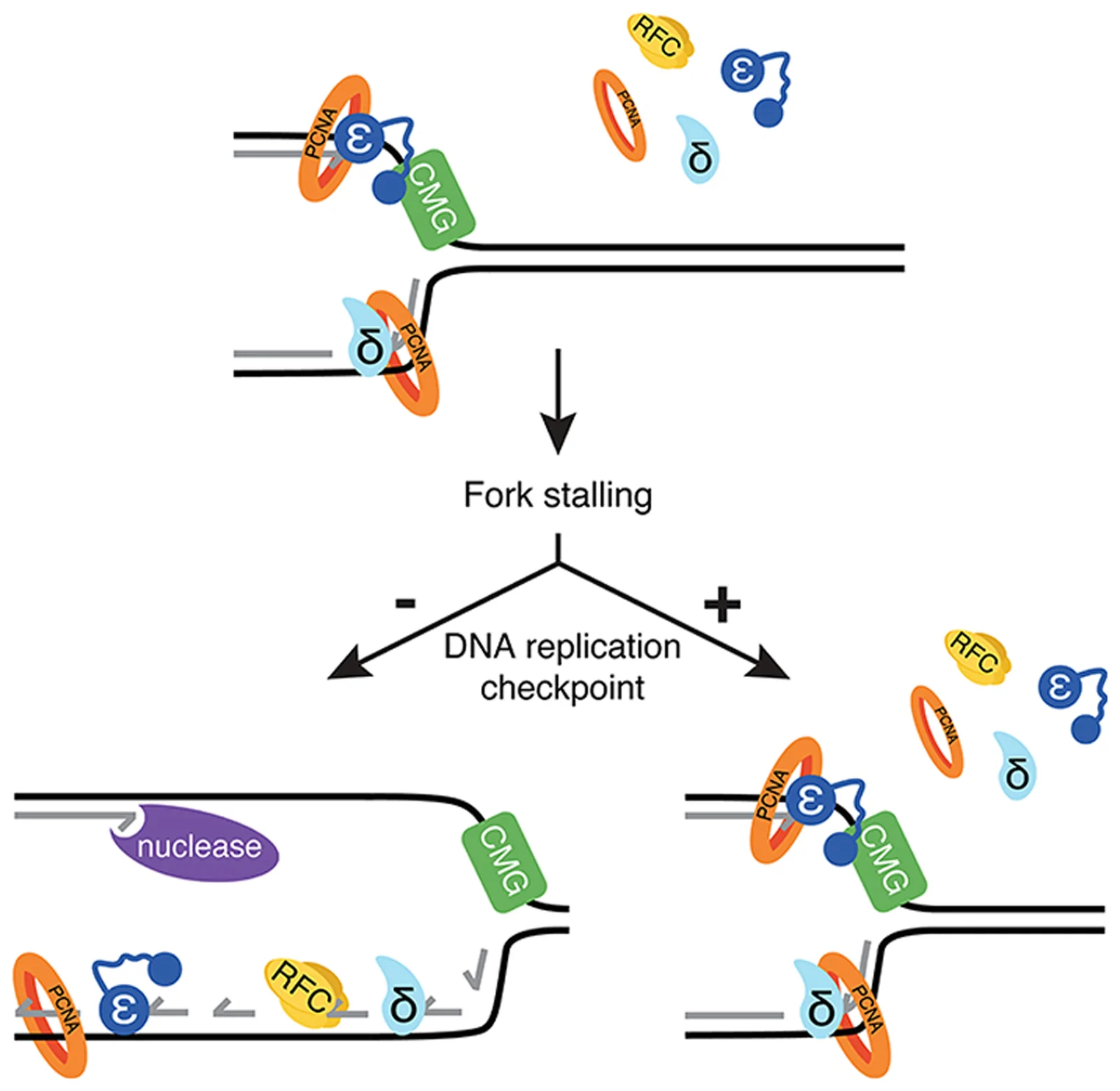

## Introduction

Accurate and efficient DNA replication is crucial for genome maintenance. During chromosomal DNA replication, DNA unwinding by the CMG (Cdc45, MCM, GINS) DNA helicase must be coupled with ongoing DNA synthesis.^1–3^ Because of the anti-parallel nature of the DNA strands, DNA synthesis occurs continuously on the leading strand and discontinuously as Okazaki fragments (OkFs) on the lagging strand. OkFs are initiated by DNA polymerase α-primase (Polα): the DNA primase generates 7- to 10-nt RNA primers that are extended as DNA ~20 nt by the DNA polymerase.^4^ Replication factor C 1–5 (RFC) loads the ring-shaped proliferating cell nuclear antigen (PCNA) sliding clamp processivity factor on the 3′ DNA end generated by Polα, promoting completion of OkF synthesis by DNA polymerase δ (Polδ). Displacement of the 5′ end of the previous OkF by Polδ generates a flap, which is cleaved by endonucleases, primarily the Flap structure-specific endonuclease 1 (Fen1), and the two OkFs are ligated together by Ligase 1 (Lig1). Leading strands of the bidirectional replisomes are generated from OkFs; the leftward leading strand comes from the first OkF made by the rightward traveling replisome and vice versa.^5–8^ When Polδ from these first OkFs reaches the CMG, DNA polymerase ε (Polε), which associates with CMG and is required for its formation, normally takes over for the duration of leading-strand synthesis. Polε associates with dual processivity factors CMG helicase and PCNA to perform bulk leading-strand synthesis.^5–8^ We refer to the proteins required for OkF elongation (PCNA/RFC/Polδ), together with Polε, as the processive DNA synthesis machinery (PrSM), that together promote fast, processive, and accurate DNA synthesis of leading and lagging strands.

DNA replication forks can stall as a result of nucleotide depletion or at DNA lesions from endogenous or exogenous sources and many pathways and proteins contribute to maintaining genome stability under such conditions.^9–12^ Among these, DNA replication checkpoint pathway, which is activated by fork stalling and promotes a coordinated cellular response to ensure cell survival,^13–17^ is particularly important. Fork stalling in the absence of the checkpoint results in replication fork collapse, accumulation of single-stranded (ssDNA), DNA damage, and cell death.^18–25^ Restoration of a functional checkpoint after fork stalling does not restore cell viability, suggesting that the checkpoint prevents a catastrophic, irreversible event associated with stalled forks.^19,21^ We currently do not fully understand how stalled replication forks restart or how the DNA replication checkpoint prevents irreversible fork collapse. In this paper, we use DNA replication with purified proteins to characterize fork stalling, restart, and the mechanism of fork stabilization by the DNA replication checkpoint.

## Results

### DNA replication fork stalling and restart *in vitro*

**Figure 1:**
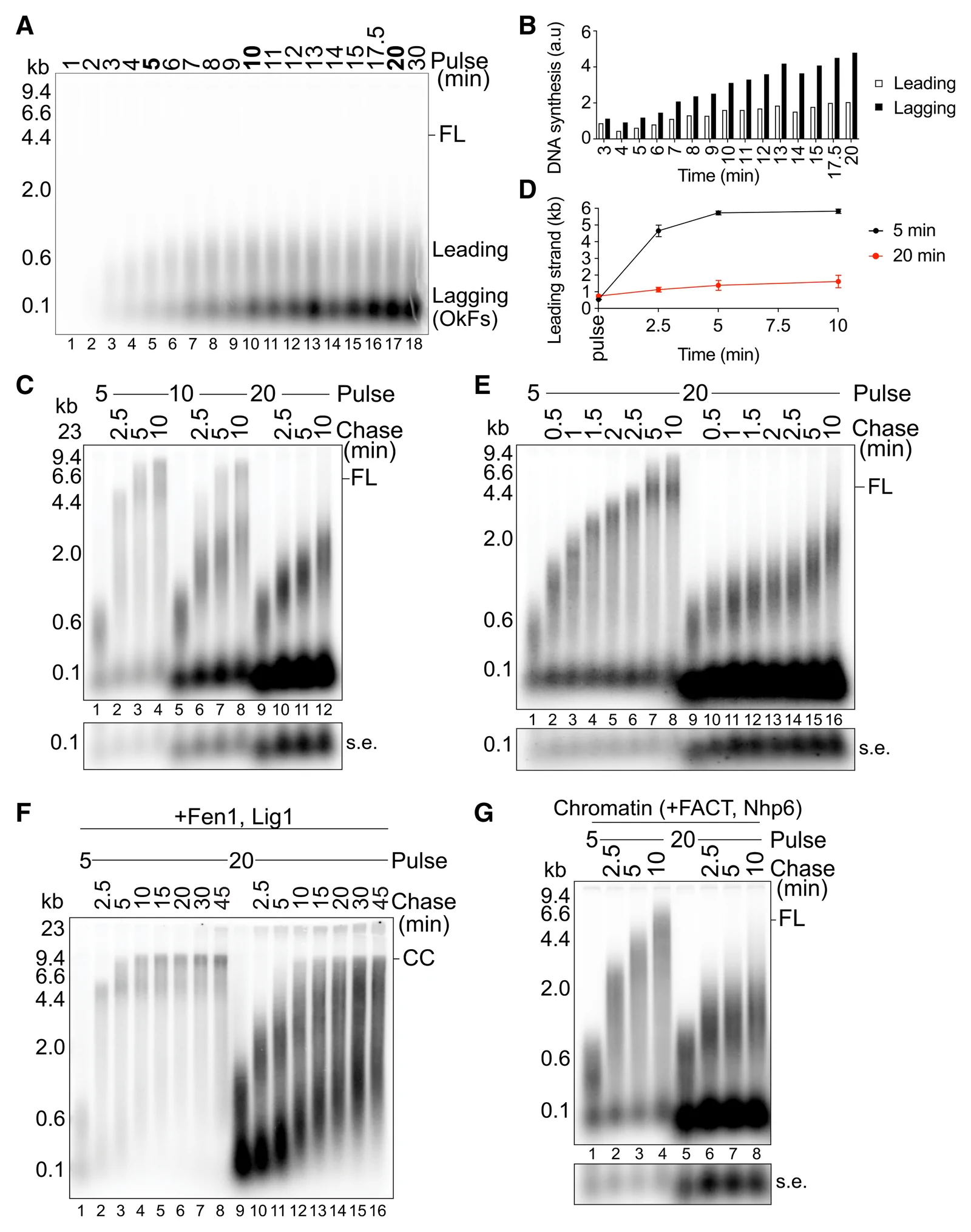
DNA replication fork stalling and restart *in vitro* **(A)** Time course of DNA replication reaction at 1 μM (low) dNTPs with exo^−^ Polε. **(B)** Quantitation of leading and lagging-strand signal intensities from (A). **(C)** Pulse-chase DNA replication reactions with WT Polε. 1 μM (low) dNTPs pulse for 5, 10, and 20 min chased each with 600 μM unlabeled dNTPs for 2.5, 5, and 10 min. Unless specified, all subsequent pulse-chase reactions followed this same experimental strategy and were done with WT Polε. **(D)** Quantitation of leading-strand replication products in kilobases (kb) for triplicate experiments performed as in (C). Error bars represent the standard deviation from three independent experiments. **(E)** Pulse-chase DNA replication reactions. 5- and 20-min low dNTP pulses chased each at 0.5 min intervals up to 2.5 min, plus 5 and 10 min. **(F)** Pulse-chase DNA replication reaction always in the presence of Fen1 and Lig1. **(G)** Pulse-chase DNA replication reactions on a chromatin template in the presence of the histone chaperones FACT and Nhp6. 5- and 20-min low dNTP pulses chased each for 2.5, 5, and 10 min. FL represents full leading-strand length. s.e. represents a shorter exposure of the Okazaki fragments (OkFs). kb, kilobases; min, minutes. See also Figure S1.

We exploited the reconstitution of DNA replication with purified budding yeast proteins^5,26^ on a circular 10.6 kb DNA template containing a single origin of replication^5^ to study the nature of DNA replication fork stalling and restart. Replication of this template at our standard deoxynucleoside triphosphate (dNTP) concentration (80 μM each dNTP) in the absence of Fen1 or ligase results in production of leading-strand products of approximately half the plasmid length (~5.3 kb), and similar levels of DNA synthesis as discontinuous OkFs in the lagging strand (~0.2–0.4 kb) within 10 min (Figure S1A). To induce DNA replication fork stalling, we performed DNA replication at several reduced dNTP concentrations (Figure S1B). At 1 μM total dNTP concentration (the “low dNTP concentration” throughout the manuscript), leading-strand DNA synthesis is initially slow (~0.12 kb/min) and then stops after 6–8 min (Figures 1A and S1C). OkFs initiate but remain short (~100 nt); they primarily comprise DNA not RNA because they have incorporated the radioactive deoxynucleotide α^32^P-dCTP and are resistant to alkaline hydrolysis during the processing of samples. OkFs continue to initiate and accumulate even after leading-strand synthesis stops, resulting in 2–3× more incorporation in the lagging strand than in the leading strand after 30 min (Figures 1A and 1B). Therefore, leading-strand synthesis stops, but OkFs continue to initiate and accumulate, suggesting that CMG continues to unwind DNA. Previous work has shown that DNA unwinding continues *in vitro* after DNA synthesis has stalled in limiting dNTPs^27^ and after leading-strand synthesis is blocked by a template lesion.^28^ Our results are also consistent with work *in vivo* using a strand-specific sequencing approach (eSPAN) that showed lagging-strand DNA synthesis continues beyond the end of stalled nascent leading strands in budding yeast *rad53* and *mec1* DNA replication checkpoint mutants.^29^

We added a high concentration of unlabeled dNTPs (chase) after different times at low dNTP concentration to promote and visualize DNA synthesis restart from stalled forks. After 5 min at low dNTP concentration, the amount of leading and lagging-strand synthesis is roughly equivalent and synthesis is still occurring slowly (Figure 1A, lane 5); dNTP addition at this point induced rapid leading-strand synthesis, reaching near full length by 2.5 min (Figures 1C and 1D). However, after 20 min at low dNTP concentration, when leading-strand synthesis has been stalled for >10 min and excess initiated OkFs have accumulated (Figure 1A, lane 17), addition of dNTPs led to leading-strand synthesis restart that was very slow and farfrom complete after 10 min (Figures 1C and 1D) and even after 60 min (Figure S1D). After 10 min at low dNTP concentration, two distinct populations of restart were observed, one fast and one slow (Figure 1C, lanes 6–8). This indicates that the difference in restart between 5 and 20 min is not a progressive slowing of restarting forks, but likely due to the acquisition of a discrete mode of slow DNA synthesis after extended fork stalling. Addition of the dNTP chase also induced the extension of the OkFs, extension that was apparently similar after 5, 10, and 20 min at low dNTP concentration (see shorter exposure [“s.e.”] in Figure 1C). Similar results were obtained when replication products were resolved on formaldehyde/formamide denaturing gels without alkaline treatment (Figure S1E), indicating that the apparent aberrant leading-strand restart is not due to alkaline hydrolysis of the nascent DNA at positions of incorporated ribonucleotides, which might have been favored after extended incubation at low dNTP concentration. Leading-strand restart speed after 5 min at low dNTP concentration is comparable to unperturbed replication (Figures 1E and S1F; Yeeles et al.^5^) and was unaffected by omission of Polδ (Figure S1G), indicating that Polε is sufficient for leading-strand synthesis during short stalling and restart. Restart after 20 min was roughly five times slower than after 5 min (0.25 kb/min vs 1.2 kb/min) (Figures 1E and S1F). Excess OkF initiation after 20 min and aberrant restart were still seen when we used a catalytically inactive Polε mutant (ΔCAT-Polε) and omitted Polδ from reactions (Figure S1H), indicating that DNA synthesis during the aberrant restart is catalyzed primarily by Polα.

Inclusion of Fen1 and Lig1, enzymes that promote OkF maturation and ligation, during stalling and restart, led to production of a completely replicated closed circular (CC) plasmid after restart of the 5 min incubation (Figure 1F). However, it did not restore normal leading-strand synthesis restart after 20 min at low dNTP concentration and most replication products never reached completion (CC). Though there was some ligation of OkFs evident during restart, this was only partial even after 45 min. Therefore, the presence of OkF maturation machinery during stalling did not suppress aberrant restart. Note that during the low dNTP pulse, no ligation was observed with Fen1 and Lig1 at 5 min at low dNTP concentration indicating that little or no OkF completion and maturation occurred even from the beginning of the reaction. Addition of Fen1 (without Lig1) to our reactions did not change the size of the OkFs generated during the low dNTP pulse (Figure S1I), consistent with OkFs being incomplete and lacking 5′ flaps.

Replication at low dNTP concentration of a chromatinized DNA template in the presence of the histone chaperone FACT^30^ was very similar to naked DNA (Figure 1G): leading-strand synthesis stopped at a similar point and excess, short OkFs continued to accumulate over time. Therefore, the core DNA replication machinery can promote continued unwinding through chromatin even when leading-strand DNA synthesis is inhibited. Similar to naked DNA, addition of dNTPs after 5 min at low dNTP concentration promoted efficient leading-strand synthesis restart whereas restart was aberrant after 20 min, indicating that the presence of chromatin does not rescue defects in replication fork restart. It is unknown whether histones from parental nucleosomes are re-deposited on the unreplicated, ssDNA behind the fork and whether any of the histone-binding domains in the replisome (e.g., Mcm2^31,32^ or Polε^33,34^) are important for this replication.

### The DNA replication checkpoint protects stalled forks

**Figure 2:**
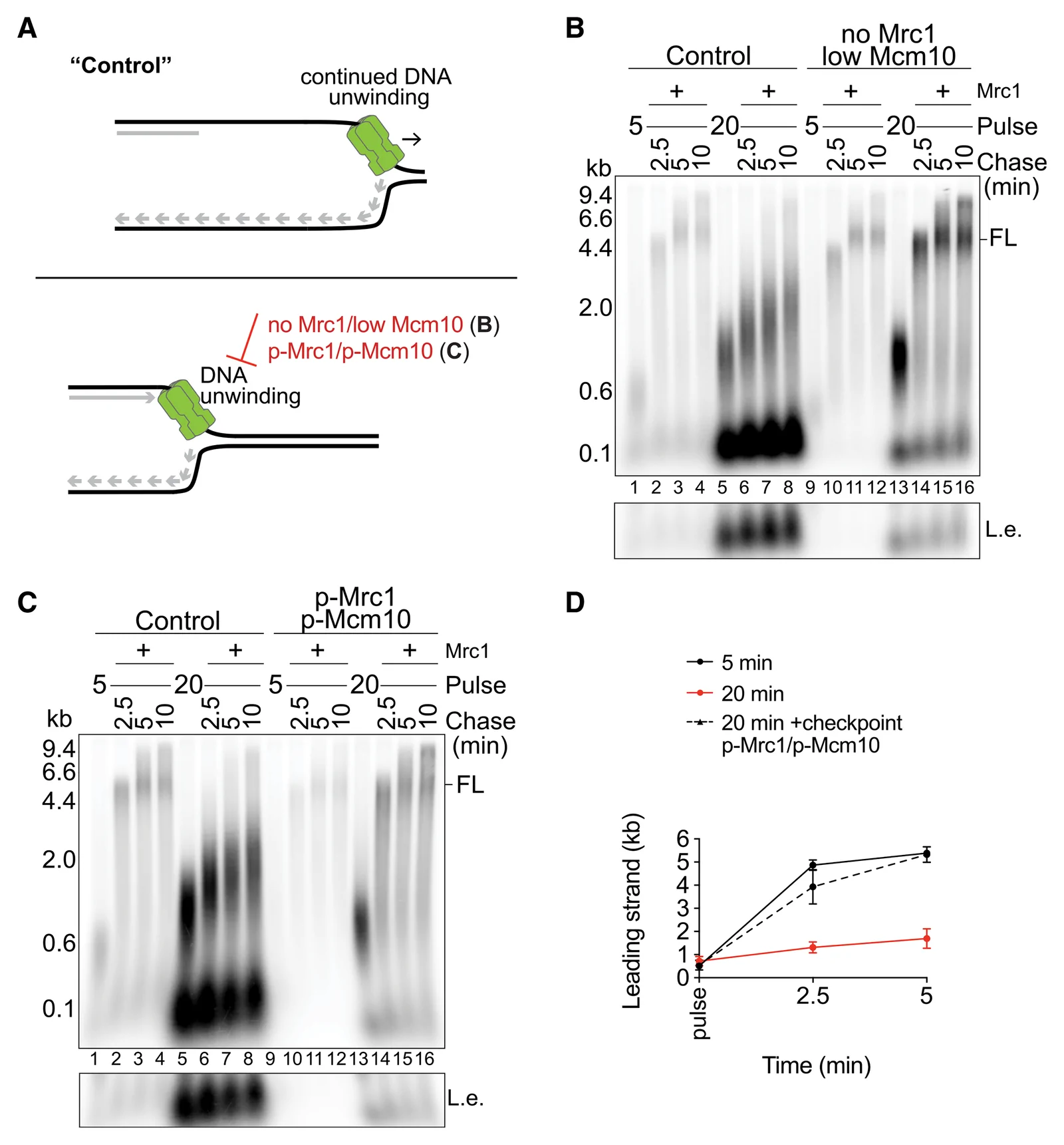
The DNA replication checkpoint protects stalled forks **(A)** Rationale scheme showing the strategies used to inhibit DNA unwinding during fork stalling in (B) and (C). **(B)** Pulse-chase DNA replication reactions. Reactions contained 20 nM Mrc1 and Mcm10 (Control) or no Mrc1 and 5 nM (low) Mcm10. 30 s before chasing for 2.5, 5, and 10 min, 20 nM Mrc1 were added to all pulses. **(C)** Pulse-chase DNA replication reactions. Reactions contained Control (unphosphorylated) or Rad53-phosphorylated 20 nM Mrc1 and Mcm10 (p-Mrc1/p-Mcm10). 30 s before chasing for 2.5, 5, and 10 min, 60 nM unphosphorylated Mrc1 was added to all pulses. **(D)** Quantitation of leading-strand replication products in kilobases (kb) for triplicate experiments performed as in (C). Error bars represent the standard deviation from three independent experiments. FL represents full leading-strand length. s.e. represents a shorter exposure of the Okazaki fragments (OkFs). kb, kilobases; min, minutes. See also Figure S2.

The mediator of the replication checkpoint 1 (Mrc1) and Mcm10 stimulate DNA unwinding by the CMG helicase and promote fast DNA replication.^5,35–37^ Omission of Mrc1 and reduction of Mcm10 concentration (Mcm10 cannot be omitted because it is required for initiation), slows replication fork progression^36–39^ and this prevented accumulation of OkFs at later times and allowed normal restart after 20 min at low dNTP concentration (Figures 2A and 2B). However, omission of Mrc1 in the presence of high Mcm10 concentration only partially reduced OkF accumulation and aberrant restart (Figure S2A), consistent with the idea that both Mrc1 and Mcm10 can accelerate CMG, which contributes to OkF accumulation and aberrant restart.

The DNA replication checkpoint kinase Rad53 slows replication forks by phosphorylating Mrc1 and Mcm10, which inactivates their ability to stimulate DNA unwinding,^35^ and, *in vivo*, deletion of *MRC1* prevents uncoupling between leading- and lagging-strand synthesis observed by eSPAN in *rad53* and *mec1* mutants.^40^ Addition of Rad53-phosphorylated Mrc1 and Mcm10 during fork stalling followed by unphosphorylated Mrc1 and Mcm10 during restart (Figure 2A), which mimics events during fork stalling and restart *in vivo*, also limited OkF generation during fork stalling and rescued restart after 20 min (Figures 2C, 2D, S2B, and S2C). Addition of Rad53-phosphorylated Mrc1 and unphosphorylated Mcm10 only partially reduced OkF accumulation and aberrant restart (Figure S2D). Therefore, the Rad53 checkpoint kinase can protect stalled DNA replication forks at least in part by preventing unrestrained DNA unwinding through Mrc1 and Mcm10 phosphorylation, consistent with previous data.^29,35–37,40^

### Excess OkF initiation prevents restart

**Figure 3:**
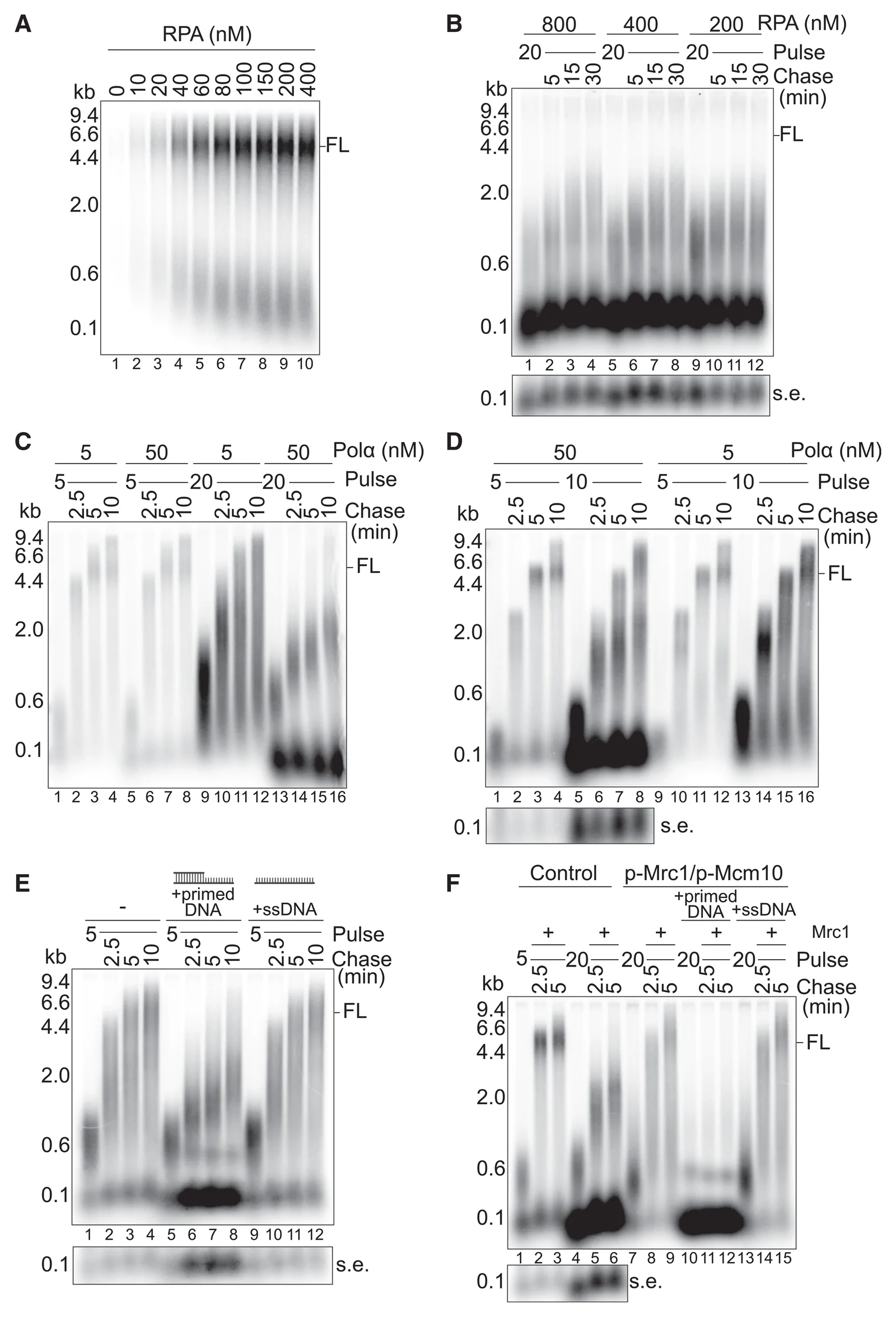
Excess OkF initiation prevents restart **(A)** DNA replication for 7.5 min with 80 μM dNTPs at increasing RPA concentrations. **(B)** Pulse-chase DNA replication reactions. 20 min pulses with 800, 400, or 200 nM RPA, chased each for 5, 15, and 30 min. **(C)** Pulse-chase DNA replication. 5- and 20-min pulses with 5 (low) or 50 nM Polα, chased each for 2.5, 5, and 10 min. (D) Pulse-chase DNA replication reactions. 5- and 10-min low dNTP pulses with 50 nM (standard) or 5 nM (low) Polα, chased each for 2.5, 5, and 10 min. **(E)** (E) Pulse-chase DNA replication with 40 nM exo^−^ Polα, chased each for 2.5, 5, and10 min.Pulse-chase DNA replication with 40 nM exo^−^ Polε. Buffer, 100 nM competitor primed DNA or ssDNA were added 4 min into the 5 min pulse and chased each for 2.5, 5 and 10 min. **(F)** Pulse-chase DNA replication reactions containing 20 nM Mrc1 and Mcm10 (Control) or Rad53-phosphorylated 20 nM p-Mrc1/p-Mcm10. Buffer, 100 nM competitor primed DNA or ssDNA were added at 5 min into the 20 min pulse. 30 s before chasing for 2.5, 5, and 10 min, 60 nM unphosphorylated Mrc1 was added to all pulses. FL represents full leading-strand length. s.e. represents a shorter exposure of the Okazaki fragments (OkFs). kb, kilobases; min, minutes. See also Figure S3.

How does unrestrained fork progression prevent normal restart after 20 min of stalling? Generation of excess ssDNA could deplete soluble Replication protein A (RPA) and affect restart, as has been postulated as a cause of fork collapse in human cells lacking a functional checkpoint.^41^ While 80 nM RPA is sufficient for maximal levels of DNA replication in the absence of stalling (Figure 3A) even ten times more RPA (800 nM RPA) did not rescue fork restart after either 20 or 10 min at low dNTP concentration (Figures 3B and S3A), arguing that RPA depletion is unlikely to be the cause of the aberrant restart observed upon fork stalling in our system.

We next asked whether continued OkF initiation and accumulation during extended CMG unwinding (Figures 1A and 1B) was responsible for the aberrant restart. The frequency of OkF initiation on naked DNA templates can be modulated by changing Polα concentration.^5^ Reducing Polα concentration during low dNTP treatment partially rescued the restart defect after 20 min (Figure 3C, compare lanes 14–16 with lanes 10–12) and completely rescued restart after 10 min at low dNTP concentration (Figure 3D, compare lanes 14–16 with lanes 6–8). Further reduction in Polα level resulted in reduced overall replication, presumably because of defects in establishing leading-strand as well as lagging-strand replication, and was therefore not interpretable.

As an alternative approach, we asked whether addition of competitor DNA mimicking either ssDNA or a short incomplete OkF could prevent normal restart after 5 min at low dNTP concentration (Figure S3B). Addition of a 61 nt ssDNA oligonucleotide (competitor ssDNA) had no effect on restart after 5 min at low dNTP concentration, while the same oligonucleotide with an anealed 30 nt complement (competitor primed DNA) blocked normal restart after 5 min at low dNTP concentration (Figures 3E and S3C) resulting in restart very similar to that seen after 20 min. Addition of the same OkF-mimic also caused aberrant restart after 20 min with Rad53-phosphorylated Mrc1 and Mcm10 (Figure 3F, lanes 11–12). Taken together, these results indicate that the continued accumulation of OkFs rather than ssDNA is the cause of the aberrant restart seen after 20 min at low dNTP concentration and that the checkpoint preserves restart by preventing the accumulation of excess OkFs.

### Depletion of PrSM factors contributes to fork stalling and causes aberrant restart

**Figure 4:**
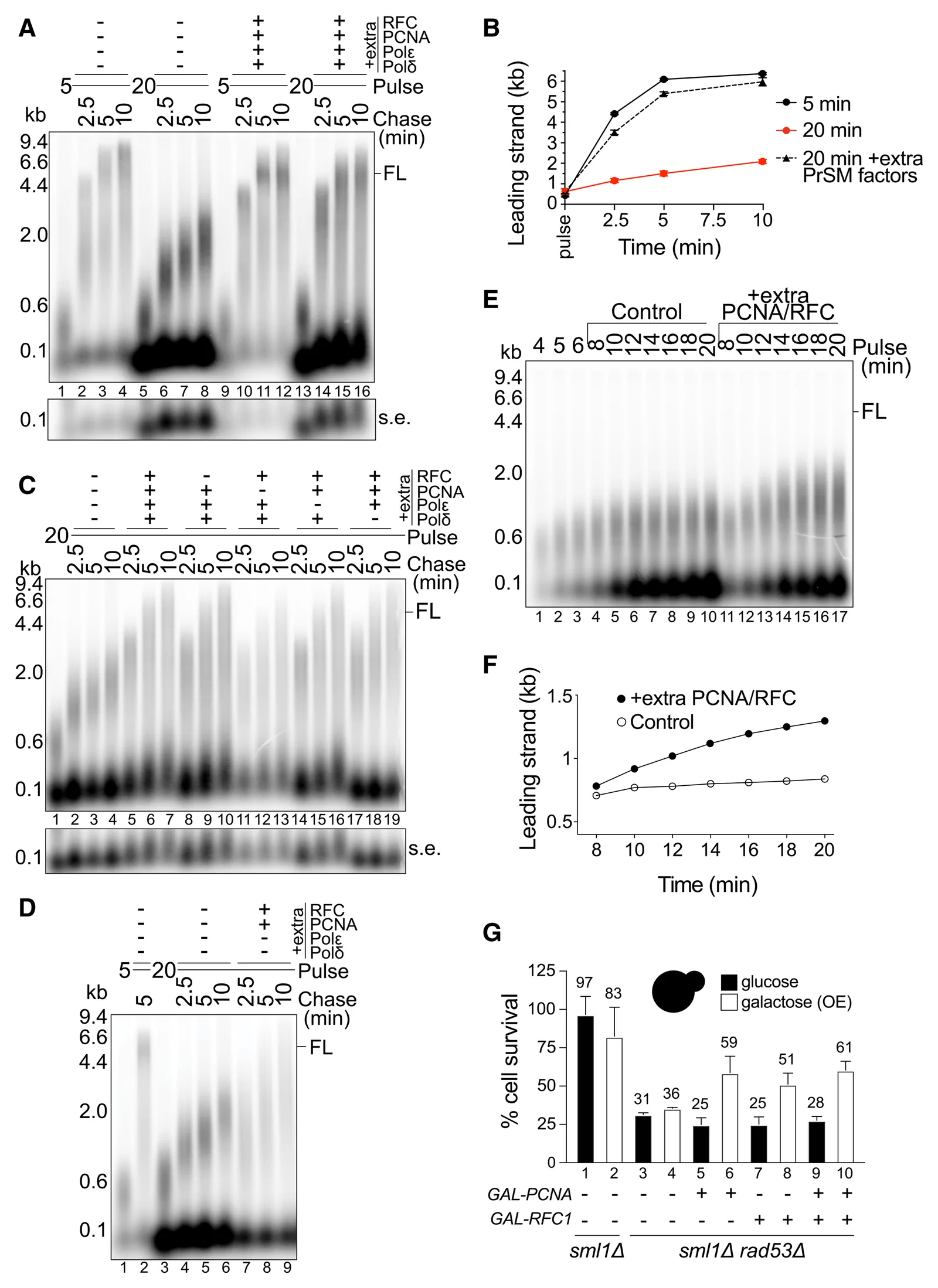
Depletion of PrSM factors contributes to fork stalling and causes aberrant restart **(A)** Pulse-chase DNA replications. 5- and 20-min pulses. 30 s before chasing, the buffers (–) or 250 nM PCNA, 125 nM RFC, 100 nM WT Polε, and 10 nM Polδ were added to each pulse. **(B)** Quantitation of leading-strand replication products in kilobases (kb) for experiments performed as in (A). Error bars represent the standard deviation from three independent experiments. **(C)** As in (A), with addition of extra individual PrSM factor DROP-OUT combinations. **(D)** As in (A), different PrSM factor combinations. **(E)** Time course of DNA replication reaction at low dNTP concentrations with WT Polε. Samples were taken at 4, 5, and 6 min before reaction was divided in two. At 7 min, either the buffers (control) or the “extra PCNA/RFC” mix containing 250 nM PCNA and 125 nM RFC were added to each reaction. Time course samples were then taken from each reaction every 2 min for 20 min. **(F)** Quantitation of leading-strand replication products in kb from (E). **(G)** Cell viability assay. Lanes 1–2: percentage of *sml1*Δ cells surviving after 1 h in 0.01% MMS-containing yeast peptone (YP)-glucose (black) and YP-galactose (white), relative to survival in α-factor. Lanes 3–10: percentage survival in MMS of *sml1*Δ*rad53*Δ cells overexpressing none, PCNA, Rfc1, or PCNA + Rfc1, relative to their survival in α-factor and to survival of *sml1*Δ cells in MMS. Average % survival shown above bars. Error bars represent standard deviation. FL represents full leading-strand length. s.e. represents a shorter exposure of the Okazaki fragments (OkFs). kb, kilobases. min, minutes. See also Figures S4 and S6.

Excess incomplete OkFs could sequester any or all PrSM factors and, consistent with this, more PCNA was loaded onto DNA after 20 min than 5 min at low dNTP concentration (Figures S4A–S4C). Moreover, addition of extra PCNA, RFC, Polε, and Polδ together rescued restart from forks stalled for 20 min at low dNTP concentration even in the presence of excess OkFs (Figures 4A and 4B). Individual dropouts showed that all four PrSM factors contributed to efficient restart after 20 min (Figure 4C), indicating that each PrSM factor is likely depleted to some extent. PCNA and RFC by themselves improved restart (Figure 4D) and dropout of either PCNA or RFC led to reduced restart (Figure 4C). Although dropout of Polε had a smaller effect than dropout of Polδ, there was a very substantial reduction in rescue when both polymerases were omitted indicating that, in the absence of extra Polδ, extra Polε can support restart (Figures 4C and 4D). After shorter time in low dNTPs (10 min), individual dropouts showed that PCNA and Polε contributed to efficient restart while extra RFC and Polδ had a very minor contribution (Figures S4D and S4E). Consistent with this, addition of extra PCNA together with Polε was sufficient to suppress aberrant restart after 10 min in low dNTPs while all four PrSM factors were required after 20 min (Figure S4F). These results suggest that PCNA and Polε are depleted by OkFs before RFC and Polδ *in vitro*.

DNA synthesis stopped in the leading strand while lagging-strand DNA synthesis continued (Figure 1A), indicating that leading-strand stalling occurred despite the continued presence of dNTPs. During the low dNTP incubation, we found that addition of extra PCNA and RFC promoted further leading-strand synthesis without additional dNTPs (Figures 4E and 4F). Therefore, the concentration of PCNA and RFC that is required and sufficient to sustain leading-strand synthesis at the beginning of the reaction at low dNTP concentration, becomes insufficient as DNA replication proceeds at low dNTP concentration, indicating that replication at low dNTP concentration leads to early depletion of PrSM factors and contribute to fork stalling. Depletion of PCNA has relatively mild effects on leading-strand synthesis during replication at high dNTP concentrations^5^; however, omission of PCNA from the beginning of the reaction at low dNTP concentration profoundly affected leading-strand DNA synthesis (Figure S4G; Yeeles et al.^5^). This residual synthesis is catalysed by Polα since similar synthesis is seen when PCNA and Polδ are both omitted in the presence of either wild-type (WT) Polε or ΔCAT-Polε (Figure S4H). Taken together, these experiments indicate that PrSM depletion contributes to fork stalling at low dNTP concentration in addition to being critical for restart.

Mrc1 phosphomimicking mutants that reduce DNA replication fork speed have been shown to partially suppress the DNA damage sensitivity of a *rad53* mutant *in vivo*.^35^ We wanted to determine whether excess PrSM factors could also suppress the effects of fork stalling in a checkpoint-deficient background. To avoid documented cell lethality associated with chronic overexpression of PCNA,^42^ we designed an experimental strategy to induce an acute overexpression of PCNA and/or Rfc1 from a galactose-inducible promoter together with entry into S phase in the presence of methyl methanesulfonate (MMS). Under these conditions, the sensitivity of a *rad53* mutant to MMS was significantly decreased by expression of Rfc1 and PCNA individually or in combination (Figures 4G, S4I, and S4J), consistent with the idea that PrSM depletion contributes to cell lethality in the absence of a functional DNA replication checkpoint.

### PrSM factors protect nascent DNA from nuclease attack

**Figure 5:**
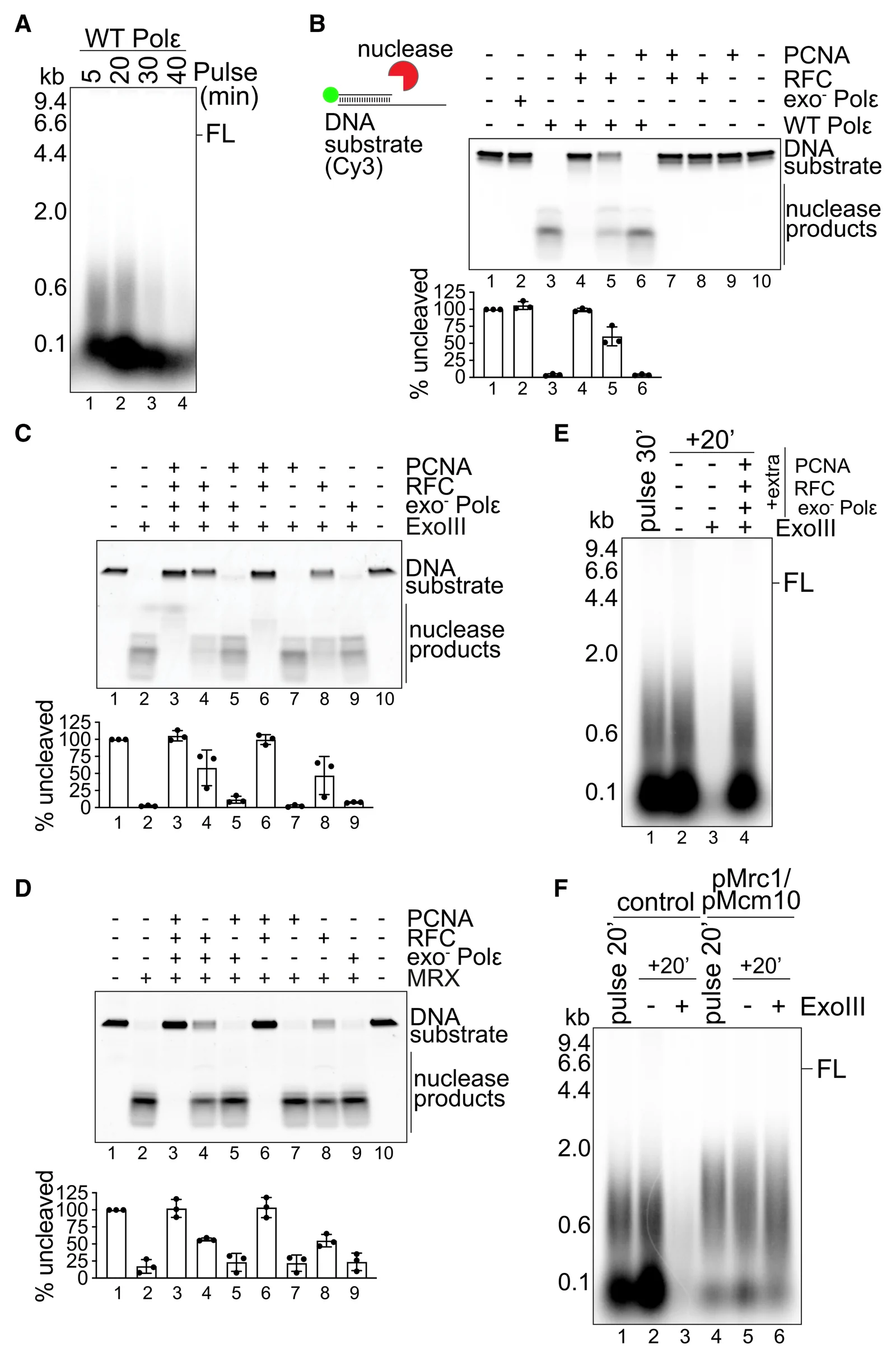
PrSM factors protect nascent DNA from nuclease attack (A) Time course of DNA replication reaction in low dNTPs with WT Polε. (B) Nuclease reactions (5 min) with 10 nM exonuclease mutant (exo^−^) or WT Polε in the absence or presence of 125 nM RFC with or without 250 nM PCNA separated in TBE-PAGE and imaged for Cy3 fluorescence. Bar graph below the gel image shows quantitation of percentage uncleaved DNA substrate. Error bars represent the standard deviation from three independent experiments. (C) Nuclease reactions (5 min) with 2U of ExoIII nuclease (NEB) in the presence of different PrSM factor combinations containing 250 nM PCNA, 125 nM RFC, 100 nM exo^−^ Polε, or their buffers. Bar graph below the gel image shows quantitationof percentage uncleaved DNA substrate. Error bars represent the standard deviation from three independent experiments. (D) As in (C), but with 100 nM MRX nuclease for 45 min. Bar graph below the gel image shows quantitation of percentage uncleaved DNA substrate. Error bars represent the standard deviation from three independent experiments. (E) DNA replication reaction in low dNTPs with exo^−^ Polε for 30 min (pulse, lane 1). Pulse sample is then divided (lanes 2–4) and incubated for 20 min at 30°C with buffer or 20U ExoIII nuclease alone or in the presence of extra 250 nM PCNA, 125 nM RFC, and 100 nM exo^−^ Polε. (F) DNA replication reaction in low dNTPs with exo^−^ Polε for 20 min in the presence of unphosphorylated (pulse, lane 1) or Rad53-phosphorylated Mrc1 and Mcm10 (pulse, lane 4). Pulses are then divided (lanes 2–3 and 5–6) and incubated for 20 min with buffer or 20U ExoIII nuclease. FL represents full leading-strand length. kb, kilobases. min, minutes. See also Figure S5.

DNA synthesis at low dNTP concentration progressed with similar dynamics using WT or exonuclease mutant (exo^−^) Polε up to 20 min, and restart was similar with both polymerases (Figure S5A). However, the signal intensity and leading- and lagging-strand lengths were reduced at later times in reactions containing WT but not exo^−^ Polε (Figures 5A, S5A, and S5B), indicating that, at later times at low dNTP concentration, the exonuclease of Polε was degrading nascent DNA.

This suggested that depletion of PrSM factors may make DNA ends available for nucleolytic attack. To test this, we initially used a primed DNA model substrate (Figure S5C). The exonuclease of Polε degrades the substrate effectively, and RFC with or without PCNA provides protection from DNA degradation by Polε (Figure 5B). We next asked whether PrSM factors could protect DNA from other 3′ to 5′ exonucleases, the bacterial ExoIII and the yeast Mre11-Rad50-Xrs2 (MRX) complex (Figures S5D and S5E). Figures 5C and 5D show that all PrSM factors contribute to maximum protection from both nucleases. Dropouts of PCNA or RFC resulted in reduced protection (lanes 4 and 5). RFC provided protection on its own (lane 8). Exo^−^ Polε provided only very weak protection in the absence of PCNA and RFC (lane 9) but in their presence contributed to maximum protection (Figure S5F).

We then asked whether PrSM factors protect nascent DNA from degradation by nucleases during replication. Addition of the ExoIII nuclease after 30 min at low dNTP concentration resulted in degradation of leading and lagging nascent DNA products, which was prevented by addition of extra PrSM factors (Figure 5E). Rad53-phosphorylation of Mrc1 and Mcm10, which resulted in reduced OkF accumulation, also prevented nucleolytic attack of nascent DNA by ExoIII after 20 min even without addition of excess PrSM factors (Figure 5F). Therefore, PrSM depletion, in addition to affecting DNA synthesis restart, exposes nascent DNA ends, making them susceptible to nucleolytic attack, and the DNA replication checkpoint prevents this by limiting OkF accumulation. Of the four PrSM factors, RFC is especially good at protecting exposed DNA ends from nuclease attack.

## Discussion

**Figure 6:**
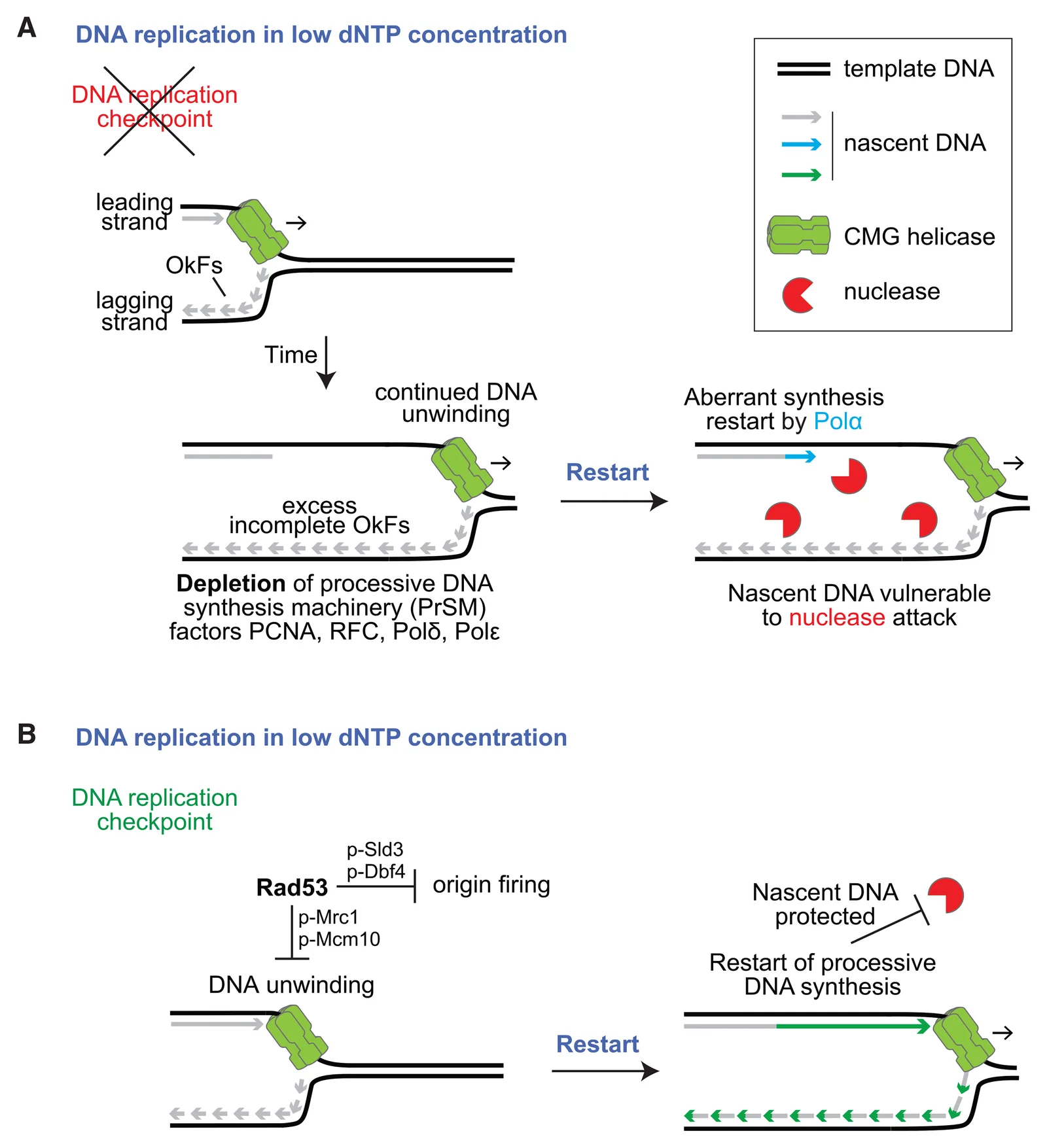
Mechanisms of checkpoint-dependent fork stabilization and restart Inhibition of DNA synthesis at low dNTP concentration causes fork stalling. When fork stalling occurs in the absence of the checkpoint (A), DNA unwinding continues and allows excess generation of incomplete OkFs, which sequester and deplete the PrSM factors. PrSM depletion results in aberrant restart by Polα and exposes nascent DNA to nuclease attack. Instead, when fork stalling occurs in the presence of the checkpoint (B), the Rad53 DNA replication checkpoint kinase prevents excess OkF generation by phosphorylating the replication proteins Mrc1, Mcm10, Sld3, and Dbf4 to inhibit DNA unwinding and origin firing respectively. By preventing accumulation of excess incomplete OkFs, the checkpoint prevents PrSM depletion, which allows processive DNA synthesis restart and protects nascent DNA from nuclease attack.

From our work using a defined, reconstituted DNA replication system a picture emerges of (1) what happens when DNA replication forks stall in response to nucleotide depletion, (2) how forks restart, and (3) how the DNA replication checkpoint protects stalled forks (Figure 6). Leading-strand synthesis at 1 μM dNTP stalls after ~5 min and ~0.6 kb of synthesis but OkF synthesis continues for at least another 15 min resulting in ~3× more incorporation. Based on the amount of synthesis (3× ~0.6 = ~1.8 kb) and the size of the OkFs (~100 nt), after 20 min at low dNTP concentration, there are approximately 18 OkFs for every leading strand. From the start, these OkFs are incomplete, since even at the 5 min time point, addition of dNTPs led to an increase in their length and inclusion of Fen1 and Lig1 did not lead to OkF ligation (Figure 1F). CMG unwinds DNA at a rate of ~0.14 kb/min in the absence of DNA synthesis and the presence of Mrc1^35^; therefore, not more than 2.7 kb of DNA would be unwound in 20 min; consequently, the 18 ~100 nt long OkFs are initiated roughly every 150 nt or less and are, therefore, more densely packed along the lagging strand than at 80 μM dNTPs, where the OkFs are ~300 nt (Figure S1A). Consistent with this, OkFs only increase in size by about 50 nt even after normal restart (see “s.e.” in Figures 1C and 1E). Therefore, OkF initiation after uncoupling from leading-strand synthesis occurs frequently along the template at low dNTP concentration. Extension of the OkFs after addition of the dNTP chase indicates that the 3′ end of OkFs are annealed to the lagging-strand template, and resistance to shortening by Fen1 (Figure S1I) indicates that the 5′ ends are also template-annealed and not flapped. Therefore, OkFs generated during dNTP depletion are annealed to the lagging-strand template, unflapped, and densely packed, and they cover most of the lagging-strand template.

Our results provide information about how DNA synthesis restarts after stalling. After a short (5 min) time at low dNTP concentration, leading-strand synthesis resumes normally even in the absence of Polδ (Figure S1G) suggesting that Polε remains engaged with the 3′ end of the leading strand even as leading-strand synthesis is slowing. We note that although Polε does not require PCNA for DNA synthesis in high dNTPs,^5^ at low dNTP concentration PCNA loading by RFC is essential for leading-strand DNA synthesis and restart by Polε (Figures 4E, S4G, and S4H; Yeeles et al.^5^). The overall rate of leading-strand synthesis at 80 μM dNTPs is increased by PCNA^5^ suggesting transient stalling or uncoupling might occur without PCNA even at normal dNTP concentrations. Lagging-strand synthesis by Polδ also appeared to restart normally after 5 min at low dNTP concentration as we saw near complete ligation of OkFs after adding dNTPs (Figure 1F).

After extended time (10–20 min) at low dNTP concentration, normal restart no longer occurs after dNTP addition. Instead, leading-strand synthesis rate after restart is very slow. Polα is sufficient for this synthesis rate, and even after extended incubation, lagging strands are not completely ligated (Figure 1F) indicating that lagging-strand synthesis restart is also aberrant. This restart resembles restart in human cells after aphidicolin arrest with checkpoint inhibitors described in the accompanying manuscript^43^ in that the rate of replication after restart is very slow and dependent upon Polα. Our results suggest that RPA depletion is not the cause of this phenomenon, consistent with work in the accompanying manuscript showing that aberrant fork restart precedes RPA exhaustion. Low levels of RPA (Figure 3A) reduce the frequency of OkF initiation without affecting the leading-strand synthesis rate, consistent with previous results using a more minimal replication system.^26^ At very low RPA levels, the amount but not the length of the leading strand is diminished (Figure 3A), suggesting that there is a defect in generating the OkF that seeds leading-strand synthesis, but once initiated, leading-strand synthesis is minimally affected by RPA depletion. In the accompanying manuscript,^43^ we show that RPA exhaustion contributes to DNA damage primarily at late times. Thus, RPA depletion may inhibit OkF synthesis at late times, contributing to more ssDNA and further RPA depletion. Aberrant restart is due to the sequestration of PrSM factors by excess OkFs, which prevents resumption of processive DNA synthesis (Figure 6). In contrast to leading-strand synthesis restart after 5 min at low dNTP concentration, for which Polε is sufficient, Polδ appears to be more important than Polε in restart after 20 min (Figures 4C and S1G). We suggest that this is because Polδ synthesizes DNA rapidly and processively with PCNA outside the context of CMG and thus is the ideal polymerase to “recouple” leading-strand synthesis to CMG,^5^ in much the same way it establishes the leading strand from an OkF during the early stages of replication^5,7,8^ and how it recouples leading-strand synthesis to CMG after translesion synthesis.^44^

Our model is supported by observations *in vivo* using strand-specific chromatin immunoprecipitation sequencing (ChIP-seq) methods showing that the DNA replication checkpoint prevents PCNA accumulation on lagging strands after hydroxyurea (HU) treatment^29^ and uncoupling of leading and lagging-strand synthesis^45^; moreover, these phenotypes are suppressed by Mrc1 deletion.^40^ Although this was attributed to accumulation of toxic ssDNA,^46^ we propose that this is instead due to accumulation of excess OkFs. Our results are also consistent with the fact that Polδ mutants show synthetic lethality with the checkpoint kinases Rad53 and Mec1 in yeast^47^ and even transient depletion of Polδ causes lethality of Mec1 mutant yeast cells.^48^ Similarly, tumor cells with compromised Polδ activity were shown to have higher vulnerability to cancer therapies using checkpoint inhibitors.^49^

We propose that, by preventing PrSM depletion, the check-point protects nascent DNA ends from enzymatic attack by exonucleases and potentially other enzymes such as helicases. PrSM factors protect 3′ DNA ends from nuclease attack, both from heterologous nucleases such as ExoIII and potentially relevant nucleases such as Mre11 and the proofreading exonuclease of Polε (Figure 5), the latter of which has been implicated in fork collapse in yeast in the absence of a functional check-point.^50^ Fork reversal is not seen in WT yeast cells in HU but has been observed in HU-treated *rad53* mutants^51^ suggesting that helicases promoting fork reversal may also gain access to nascent DNA after PrSM depletion. The physiological significance of this is unclear since budding yeast lack many of the enzymes required for fork reversal in mammalian cells including SMARCAL1 and ZRANB3.

The requirements for protection and restart are not identical; RFC by itself is sufficient for some protection but not for restart, and thus its depletion may have the most profound consequences *in vivo*, as argued in the accompanying manuscript.^43^ A recent report^52^ indicates that RFC can bind to 5′ as well as 3′ DNA ends, which could also protect nascent DNA from attack by 5′ to 3′ nucleases like Exo1.^53,54^ Regardless, PrSM factors may also indirectly protect DNA ends by promoting OkF completion and ligation. The DNA replication checkpoint modulates the enzymatic activity of several nucleases directly,^50,53,55,56^ which also contributes to protection of nascent DNA ends.

We do not know how much of the irreversible loss of viability upon fork stalling in the absence of the checkpoint is due to enzymatic attack of nascent DNA facilitated by PrSM depletion and how much is due to the irreversible loss of processive leading and lagging-strand synthesis after PrSM depletion in yeast. Loss of the Exo1 nuclease prevented degradation of stalled forks and improved cell viability in certain backgrounds^21,51,54^ and the exonuclease activity of Polε has been implicated in cell death in checkpoint mutants.^50^ In our reconstituted system, loss of processive DNA synthesis after PrSM depletion was irreversible unless new PrSM factors were added to the reaction. Irreversibility was still observed when we added Elg1-Rfc2-5, the PCNA unloader (Figures S6A and S6B) even after pre-phosphorylation by Rad53^29^ (Figures S6C and S6D). This is not very surprising for three reasons: first, excess OkF initiation depletes all four PrSM factors, not just PCNA. Second, after unloading PCNA, there still remains an excess of 3′ ends from incomplete OkFs competing with the 3′ end of the leading strand, and third, Elg1-Rfc2-5 was proposed to unload PCNA after OkF maturation,^57^ but maturation does not occur at low dNTP concentration after addition of Lig1 and Fen1 (Figure 1F), presumably because they cannot act on incomplete OkFs and they require interaction with PCNA, and so may also be compromised after PrSM depletion. Further work is needed to understand how PCNA loading and unloading is regulated in unperturbed and stalled forks.

Given these uncertainties, it appears that prevention of PrSM depletion is the best strategy for survival, and our results indicate that the DNA replication checkpoint plays a crucial role in preventing PrSM depletion by slowing unwinding from existing forks through phosphorylation and inhibition of Mrc1 and Mcm10. Prevention of continued origin firing through Rad53-phosphorylation of Sld3 and Dbf4 likely also contributes to preventing excessive OkF synthesis and PrSM depletion.^58,59^

### Limitations of the study

Our *in vitro* reconstitution of DNA replication stalling and restart in response to nucleotide depletion has allowed us to identify excess generation of incomplete OkFs and PrSM factor depletion as source of fork collapse. However, additional proteins present *in vivo* that were not included in our reconstituted experiments might also modulate this process. For example, nucleases and helicases present in cells could regulate stalled forks and change the extent of OkF accumulation. Also, the existence of other DNA polymerases, such as the translesion DNA polymerases, or the PCNA unloading pathway could change how and when PrSM factors become depleted. With our reconstituted approach, we also have described how the checkpoint kinase Rad53, by inhibiting DNA unwinding, prevents PrSM depletion and protects stalled forks. However, the checkpoint has other functions in cells including regulation of origin firing, dNTP production and gene expression, all of which could potentially contribute to prevent PrSM depletion and protect stalled forks.

## Star★Methods

### Key Resources Table

**Table:** 

REAGENT or RESOURCE	SOURCE	IDENTIFIER
Antibodies		
anti-PCNA [5E6/2]	The Francis Crick cell services facility	AB_3323859
anti-Mcm6 (rabbit polyclonal)	custom-made	N/A
anti-Flag [M2]-HRP conjugated	Sigma-Aldrich	AB_439702
anti-Rad53 (rabbit polyclonal)	Abcam (ab104232)	AB_2687603
anti-PGK1 [22C5D8]	Abcam (ab113687)	AB_10861977
Bacterial and virus strains		
BL21-Codon Plus (DE3) - RIL competent cells	Agilent	Agilent part number 230245
Rosetta™ 2 (DE3) pLysS Singles™ competent Cells	Merck	Merck 71401
T7 Express *lysY* competent cells	New England Biolabs	NEB C3010I
Chemicals, peptides, and recombinant proteins		
dNTP set	Thermo Fisher Scientific	10297018
NTP set	Thermo Fisher Scientific	R0481
[alpha-P32]dCTP	Hartmann Analytic	SRP-205/250
NP-40 Surfact-Amps™ Detergent Solution	Thermo Fisher Scientific	85124
TWEEN® 20	Sigma	P1379
cOmplete™, Mini, EDTA-free protease inhibitor cocktail	Sigma	11873580001
Biotin-16-dUTP	Jena Bioscience (2B Scientific)	NU-803-BIO16-S
MagStrep “type 3” Strep-Tactin beads	Iba-lifesciences (Fisher)	2-1613-002
10x Buffer BXT	Iba-lifesciences (Fisher)	2-1042-025
Streptavidin	Thermo Fisher Scientific	434301
BioRad Protein Assay Dye Reagent Concentrate	BioRad	500-0006
Quick Coomassie stain	Generon (Clinisciences Ltd)	NB-45-00078-1L
Phusion™ High-Fidelity DNA Polymerase	NEB	M0530L
UltraPure Phenol:Chloroform:Isoamylalcohol	Thermo Fisher Scientific	15593031
SYBR™ Gold Nucleic Acid Gel Stain (10,000X Concentrate in DMSO)	Thermo Fisher Scientific	S11494
Novex TBE Running buffer	Thermo Fisher Scientific	LC6675
Novex TBE gels, 20%	Thermo Fisher Scientific	EC63152
Ficoll® solution	Sigma	F5415
Methyl methanesulfonate (MMS), 5g	Thermo Fisher Scientific	H55120.06 (15401278)
Nocodazole	Sigma	M1404
TCA solution	Sigma	T0699
3X FLAG peptide	Chemical Biology STP at the Francis Crick Institute	N/A
RNase A, DNase and protease-free (10 mg/mL)	Thermo Fisher Scientific	EN0531
TEV Protease	NEB	P8112S
Exonuclease III	NEB	M0206S
Proteinase K	Merck	1.07393.0010
Recombinant proteins		
Budding yeast recombinant proteins	This study	See Table S3 for details of protein expression and purification strategies
ORC	Frigola et al.^60^	N/A
Cdc6	Frigola et al.^60^	N/A
MCM-Cdt1	Coster et al.^61^	N/A
DDK	On et al.^62^	N/A
Dpb11	Yeeles et al.^26^	N/A
Pol ε	Yeeles et al.^26^	N/A
exo- Polε	Goswami et al.^63^	N/A
ΔCAT Polε	Yeeles et al.^5^	N/A
GINS	Yeeles et al.^26^	N/A
Cdc45	Yeeles et al.^26^	N/A
S-CDK	Yeeles et al.^5^	N/A
Sld3/Sld7	Yeeles et al.^26^	N/A
Sld2	Casas-Delucchi et al.^64^	N/A
RPA	This study	N/A
Ctf4	Yeeles et al.^26^	N/A
TopI	Yeeles et al.^5^	N/A
TopII	Yeeles et al.^26^	N/A
Polδ	Yeeles et al.^5^	N/A
Rfc1-5 (RFC)	Yeeles et al.^5^	N/A
PCNA	Yeeles et al.^5^	N/A
Mrc1 (3xFlag)	McClure and Diffley^35^	N/A
Csm3/Tof1	Yeeles et al.^5^	N/A
Polα	Yeeles et al.^26^	N/A
Mcm10	Yeeles et al.^26^	N/A
Fen1	Posse et al.^65^	N/A
Cdc9	Posse et al.^65^	N/A
FACT	Kurat et al.^30^	N/A
Nhp6	Kurat et al.^30^	N/A
Rad53	McClure and Diffley^35^	N/A
MRX	This study	N/A
Elg1-Rfc2-5	This study	N/A
Deposited data		
Original gel and western blot images for figures	This study	Mendeley data: https://doi.org/10.17632/n9dbmtkds7.1
Experimental models: Organisms/strains		
*S. cerevisiae* strains	This study	See Table S1 for details of strains generated in this study.
yBC107 (MRX purification)	This study	N/A
yBC155 (Elg1-Rfc2-5 purification)	This study	N/A
yBC139(*sm/f*)	This study	N/A
yBC320 *(rad53)*	This study	N/A
yBC296 *(rad53* + Overexpression PCNA)	This study	N/A
yBC297 *(rad53* + Overexpression Rfc1)	This study	N/A
yBC298 *(rad53* + Overexpression PCNA/Rfc1)	This study	N/A
Oligonucleotides		
Nuclease substrate Cy3-30: */5Cy3/GGGT* *GAACCTGCAGGTGGGCAAAGATGTCC*	IDT	N/A
Nuclease substrate 60: *ACGCTGCCGAA* *TTCTACCAGTGCCTTGCTAGGACATCT* *TTGCCCACCTGCAGGTTCACCC*	IDT	N/A
Competitor primed DNA Biotin-61: */5Biotin/GACGCTGCCGAATTCTA CCAGTGCCTTGCTAG GACATCT TTGCCCACCTGCAGGTTCACCC*	IDT	N/A
Competitor primed DNA Biotin-30: */5Biotin/GGGTGAACCTGCAGGTG GGCAAAGATGTCC*	IDT	N/A
Competitor ssDNA Biotin-61-Biotin: */5Biotin/GACGCTGCCGAATTCTAC CAGTGCCTTGCTAG GACATCTTT GCCCACCTGCAGGTTCACCC/3Biotin/*	IDT	N/A
Recombinant DNA		
DNA plasmids	This study	See Table S2 for details of plasmids generated in this study.
pBC42 (Rad50 of MRX purification)	This study	N/A
pBC43 (Mre11 and Xrs2 of MRX purification)	This study	N/A
pBC52 (Elg1 of Elg1-Rfc2-5 purification)	This study	N/A
pBC65 (GAL-PCNA for in vivo experiments)	This study	N/A
pBC66 (GAL-RFC1 for in vivo experiments)	This study	N/A
Software and algorithms		
ImageJ (v1.53t)	National Institute of Health	https://imagej.net/ij/
Photoshop	Adobe	https://www.adobe.com/uk/products/photoshop.html
Illustrator	Adobe	https://www.adobe.com/uk/products/illustrator.html
Prism (v10.2)	GraphPad	https://www.graphpad.com/features
FlowJo (v10.8.2)	Becton Dickinson & Company (BD)	https://www.flowjo.com/flowjo/download
EndNote (v20.6)	Clarivate	https://endnote.com/downloads/
Other		
HiTrap Blue High-Performance column	GE Healthcare (Sigma)	GE17-0413-01
Bio-Scale Mini CHT Type I Cartridge	BioRad	#7324322
MonoQ 5/50 GL	GE Healthcare (Sigma)	17-5166-01
Superdex 200 Increase 10/300 GL	GE Healthcare	28990944
Superose® 6 Increase 3.2/300	VWR	GE29-0915-98
Hi Load 16/600 superdex 200 column	VWR	28-9893-35
HiTrap Heparin HP	GE Healthcare (Sigma)	17-0406-01
HiTrap DEAE FF	VWR	17-5055-01
HiTrap SP FF	Merck	17-5157-01
Amicon Ultra 15 ml, 100 kDa	Merck	UFC910024
D-Tube Dialyzer Mini, MWCO 6-8kDa	Merck	71504-3
Illustra G50 columns	GE Healthcare (Sigma)	GE27-5330-02

### Experimental Model and Study Participant Details

#### Yeast strains and growth conditions

To express recombinant proteins and to perform the *in vivo* experiments in Figure 4G, this study used budding yeast *S. cerevisiae* strains constructed in the background W303. Cells were grown in yeast peptone (YP) medium containing 2% glucose (YPD), 2% raffinose, or 2% raffinose + 2% galactose as carbon source at 30°C.

#### Bacteria

To express recombinant proteins, this study also used *E. coli* competent cells of the following backgrounds: BL21-Codon Plus (DE3) - RIL, Rosetta™(DE3) pLysS, and T7 Express LysY competent cells. Cells were grown in Lysogeny broth (LB) medium containing the correct antibiotics at 37°C.

### Method Details

#### Plasmids and yeast strains

Yeast strains generated in this study are listed in Table S1. Full sequence maps of the plasmids generated in this study can be found in Table S2.

To perform cell viability assays, strains were derived from diploid W303 strain containing SML1 and RAD53 gene deletions and transformed with plasmids pBC65 (Gal-PCNA) and pBC66 (Gal-3xFlag-Rfc1) and integrated after linearization with StuI and BsgI restriction enzymes into the URA and TRP loci, respectively.

To express recombinant MRX complex, yBC107 strain was constructed in the background yeast strain yJF1^60^ by sequentially integrating plasmid pBC42 (Rad50) linearised with NheI into the HIS3 locus, and then plasmid pBC43 (Mre11 + Xrs2-TEV-3xFlag) linearised with StuI into URA3 locus.

To express recombinant Elg1-Rfc2-5 complex, yBC155 strain was constructed in the background yeast strain yAE36, previously used in the construction of Rfc1-5 expressing strain yAE41,^5^ by integrating plasmid pBC52 (Elg1-3xFlag) linearised with NheI into HIS3 locus.

Strains for all other recombinant protein expression are as described^5,26^ except for Dpb11 that was expressed in yeast strain yVP8,^35^ and Sld2 and RPA that were expressed in *E.coli* from plasmids pGC441^64^ and pJM126 (Addgene) respectively.

#### Protein expression and purification

A summary of protein purification strategies used in this study can be found in Table S3. Proteins were expressed and purified as in^5,26,30,35,60–62,64,65^ with the modifications detailed here. S-CDK purification included a MonoQ column prior to gel filtration. Protein was loaded and washed with 10 column volumes (CV) of 150 mM potassium acetate buffer and eluted with 20CV linear gradient 150-1000 mM potassium acetate before loading to Superdex 200. Csm3/Tof1 was purified excluding the MonoQ column. exo^−^ Polε^63^ was purified like WT Polε except elution from heparin column was done with a linear gradient of 400-1500 mM potassium acetate. ΔCATPolε was purified as described.^5^ Rad53 was purified as described,^35^ using storage buffer containing 25 mM HEPES-KOH pH 7.6, 300 mM NaCl, 10% glycerol, 0.02% NP-40 and 1 mM DTT. Sld2 was expressed in BL21-Codon Plus (DE3) - RIL competent cells from plasmid pGC441.^64^ FACT and Nhp6 were purified as described.^30^ Elg1-Rfc2-5 was expressed in budding yeast and purified like the Rfc1-5 complex (see Tables S1–S3 for details).

Untagged RPA was purified as described^66^ with the following modifications. BL21-Codon Plus (DE3) - RIL competent cells were transformed with plasmid pJM126 and grown overnight at 37°C without shaking in LB medium supplemented with 100 μg/ml ampicillin and 50 μg/ml chloramphenicol. In the morning, cells around OD 0.1-0.2 were shaken until OD 0.5 before adding 0.4 mM IPTG for 2h at 37°C. From here, all steps were performed on ice or at 4°C. Cells were lysed in RPA buffer (25 mM HEPES pH 7.5, 10% glycerol, 1 mM EDTA, 0.02% NP-40, 1 mM DTT) with 0.5 M NaCl (0.5M NaCl Buffer) supplemented with cOmplete™, EDTA-free Protease Inhibitor Cocktail (Sigma) doing 2 rounds of freeze-thaw cycles and sonication in ice-water for 5 min in cycles of 5 sec ON, 10 sec OFF at 35%. Lysate was first clarified at 45 krpm for 45 min at 4°C and then passed through a 0.22 uM filter. The clarified supernatant was applied to a 5 ml HiTrap® Blue High-Performance column connected to an AKTA Pure, and protein eluted sequentially with 1CV 0.5M NaCl Buffer, 1.5CV of 0.8M NaCl Buffer, 1.5CV of 0.5M NaSCN Buffer and 2CV 1.5M NaSCN Buffer. Pooled peak fractions were then loaded onto a 5 ml Bio-Scale Mini CHT Type I Cartridge (Biorad) in 10 mM NaH_2_PO_4_ Buffer and eluted sequentially with 2CV 40 mM NaH_2_PO_4_ Buffer, 120 mM NaH_2_PO_4_ Buffer and 500 mM NaH_2_PO_4_ Buffer. Most protein eluted in the 40 mM NaH_2_PO_4_ Buffer. Pooled peak fractions were dialyzed in 150 mM NaCl Buffer and loaded onto a 1 ml Mono Q column, washed with 20CV 150 mM NaCl Buffer, and eluted in a 20CV linear gradient from 150-1000 mM NaCl (RPA elutes ~250 mM NaCl). Peak fractions were pooled and concentrated before loading onto a Superdex200 in 150 mM NaCl Buffer, after which peak fractions were pooled, concentrated, aliquoted and stored at -80°C.

MRX was expressed in budding yeast (see Tables S1 and S2 for yeast expression strain and plasmid details). 8L cells were grown at 30°C to OD 1 in YP + 2% raffinose and protein expression was induced by addition of galactose to 2% for 2h. Cells were lysed with a Freezer/Mill in Buffer MRX (25 mM HEPES-KOH pH 7.6, 10% Glycerol, 1 mM EDTA, 0.02% NP-40-S and 1 mM DTT) with 500 mM NaCl (Buffer MRX 500) and protease inhibitors. All subsequent steps were conducted at 4°C. Lysate was cleared at 45,000 rpm for 45 min at 4°C. Cleared lysate and 2 ml Flag bead slurry were incubated 2h at 4°C before the resin was collected, and subsequently washed with 50 ml Buffer MRX 300, incubated/washed 10 min in Buffer MRX 300 with 10 mM Magnesium acetate and 0.55 mg/ml ATP and washed again with 50 ml Buffer MRX 300. MRX was eluted in 2 subsequent steps containing 0.5 mg/ml and 0.25 mg/ml Flag peptide in 5 ml Buffer MRX 300. The eluate was then slowly diluted 3-fold by addition of Buffer MRX (no salt) before loading onto a 1 ml Heparin in Buffer MRX 100, washed with 10CV and eluted with linear gradient 100-1000 mM NaCl. Pooled peak fractions were concentrated to 0.4 ml and separated through a Superose6 gel filtration column in Buffer MRX 200. MRX containing fractions were pooled and concentrated to ~ 0.5 mg/ml, aliquoted and stored at -80°C.

### DNA replication reaction with purified proteins

Replication reactions were performed as described in the original publication.^5,65^ For every replication reaction, MCMs were loaded for 20 min at 30°C and 1250 rpm using 40 nM ORC, 40 nM Cdc6, 60 nM MCM-Cdt1, 4 nM 10.6kb ARS1-containing template DNA and 5 mM ATP in replication buffer, adjusted to final concentrations of 25 mM HEPES-KOH pH 7.6, 100 mM potassium glutamate, 10 mM magnesium acetate, 0.02% NP-40-S and 2 mM DTT (5 μl loading reactions). After 20 min of MCM loading, 50 nM DDK (in 5 μl) was added and incubated for 15 min. Then, on ice, a protein mix was added to give final concentrations of 40 nM Dpb11, 20 nM Polε, 20 nM GINS, 80 nM Cdc45, 20 nM CDK, 25 nM Sld3/7, 50 nM Sld2, 400 nM RPA, 20 nM Ctf4, 10 nM TopoI, 10 nM TopoII, 1 nM Polδ (unless specified), 25 nM PCNA, 30 nM Rfc1-5 (RFC), 20 nM Mrc1, 20 nM Csm3/Tof1, 50 nM Polαand 20 nM Mcm10 (10 μl replication reaction). In Figure 1F, 40 nM Fen1 and 40 nM Cdc9 (Lig1) were added. Finally, reactions were started by bringing the volume up to the total 10 μl with a ‘nucleotide’ mix containing 200 μM of each NTP (CTP, GTP, UTP), 1 μM (low) or 80 μM (control=high) dNTPs, and 10 nM (low) or 33 nM (control=high) α^32^P-dCTP in replication buffer adjusted to increase the final salt concentration to 225 mM potassium glutamate. Unless specified, all DNA replication reactions were done with WT Polε. pulse-chase reactions wereinitiated with 5, 10 or 20 min pulses containing 1 μM (low) dNTP and 10 nM α^32^P-dCTP, and then each pulse was chased with 600 μM non-radioactive dNTPs for the specified times. ‘dNTP’ concentrations reflect the concentration of each dNTP (dCTP, dGTP, dTTP and dATP) in a mix. Reactions were stopped by the addition of 85 mM EDTA in ice, cleared from free nucleotides over an Illustra MicroSpin G-50 column, and denatured in sample buffer at final concentration of 2% sucrose, 0.02% BPB, 60 mM NaOH, 10 mM EDTA, separated on 0.8% alkaline agarose gels/running buffer containing 30 mM NaOH and 2 mM EDTA at approximately 1 volt/ cm for 14-17h at room temperature, fixed in cold 5% trichloroacetic acid 30 min, dried on Whatman paper, exposed to phosphor screens, and scanned using a Typhoon phosphor imager.

For formaldehyde/formamide denaturing of DNA replication samples in Figure S1E, samples cleared in G-50 column were incubated in 0.3M NaCl for 2h at 55°C before denaturing DNA with final 9% formaldehyde and 50% formamide at 95°C for 5 min. Sample buffer was added containing final 20% glycerol, 1 mM EDTA pH 8.0 and 0.02% BPB/Xylene cyanole and then run in 0.8% agarose gels containing 6.66% formaldehyde and 1x MOPS-NaOH buffer pH 7 (20 mM MOPS free acid, 5 mM Na-Acetate anhydrous pH 5, 1 mM EDTA pH 8) with 1x MOPS-NaOH buffer pH 7 running buffer. Running conditions and gel processing were done as above.

For DNA replication on chromatin in Figure 1G, 50 μg of purified histone octamers were mixed with 50 μg of the 10.6 kb DNA template to a final volume of 200 μL in chromatin buffer (25 mM HEPES-KOH pH 7.6, 1 mM EDTA) containing 1 M NaCl. The histone-DNA mix was loaded into a D-TubeTM Dialyser Mini (Merck Millipore) and dialysed at 4°C against 0.5 L chromatin buffer of decreasing salt concentrations (1 M NaCl for 3 h, 0.75 M NaCl overnight, 0.5 M NaCl for 5 h, 0.0025 M NaCl overnight). After the final dialysis step, the dialysate is applied to a Superose 6 Increase 3.2/300 column (Cytiva) equilibrated in 25 mM HEPES-KOH pH 7.6, 150 mM NaCl. Peak fractions containing reconstituted chromatin were pooled and assessed by micrococcal nuclease digestion. Chromatin was dialysed against storage buffer (25 mM HEPES-KOH pH 7.6, 5 mM NaCl, 0.1 mM EDTA) and stored at 4°C.

For preparation of competitor DNAs used in Figures 3E and 3F, the oligonucleotides (see Figure S3B and key resources table for details) were annealed to a final concentration of 5 μM each in 20 mM Tris–HCl pH 7.5, 5 mM magnesium acetate and 100 mM NaCl (100 μl reaction) by heating at 95°C for 2 min and letting cool down to 25°C. Competitor primed DNA was aliquoted and kept at -80°C.0.5 μM competitor primed DNA or competitor ssDNA oligo was then incubated at with 2.5 μM Streptavidin (Invitrogen) in replication buffer (20 μl reactions) for 10 min in ice before adding to DNA replication reactions at final concentrations of 100 nM.

#### Rad53 kinase reaction

For kinase reactions in Figures 2C, S2D and 3F, Mrc1 and/or Mcm10 were incubated with Rad53 protein or buffer at 1:1:2 (Mrc1: Mcm10:Rad53) or 1:1 (Mrc1:Rad53) molar ratio in the presence of 5 mM ATP in replication buffer for 30 min prior to adding to 10 μl DNA replication reactions at final concentrations of 20 nM Mrc1 and 20 nM Mcm10.

For kinase reactions in Figure S6D, Elg1-Rfc2-5 and Rad53 or their buffers were incubated at 1:1 (Elg1-Rfc2-5:Rad53) molar ratio in the presence of 5 mM ATP in replication buffer for 30 min prior to adding to 10 μl DNA replication reactions at final concentration of 30 nM Elg1-Rfc2-5.

#### Pull down of nascent DNA

DNA replication reaction (x10 reactions/condition, total 100 μl each) was performed as above but containing 100 nM RFC, 400 nM PCNA, and 1 μM dATP/dCTP/dGTP and (1:1) 7.5 μM dTTP and 7.5 μM Biotin-dUTP for 5 and 20 min. 600 μM dTTP was added at 5 min into the 20 min reaction to prevent/chase further incorporation of Biotin-dUTP to nascent DNA. Reactions were stopped by the addition of 85 mM EDTA on ice and cleared from free nucleotides over an Illustra MicroSpin G-50 column. At this point, 10% of volume was collected as “total” sample (5% for western blot and 5% for alkaline agarose electrophoresis). MagStrep “type 3” Strep-Tactin beads were pre-equilibrated in pull down buffer (25 mM HEPES-KOH pH 7.6, 50 mM NaCl, 2 mM EDTA, 0.02% NP40-S and 2 mM DTT) and incubated with remaining sample for 30 min at 25°C and 1250 rpm. Beads were then bound to magnet, 10% of unbound volume was collected as “flow through” sample (and divided as above), and beads were washed 3 times with 100 μl pull down buffer. DNA was then eluted from beads incubating 15 min at 25°C and 1250 rpm in 20 μl elution buffer (25 mM Tris-Cl pH 7.5, 150 mM NaCl, 1 mM EDTA and 50 mM Biotin). Pull down sample was divided 80% for western blot and 20% for alkaline agarose electrophoresis. Samples for alkaline agarose gel electrophoresis were processed as above. Samples for western blot were denatured in SDS-DTT sample buffer, boiled, and separated on 10% TGX gel (BioRad) before blotting to nitrocellulose membrane. Membrane was blotted sequentially with anti-PCNA ([5E6/2], mouse monoclonal) and anti-Mcm6 (custom-made, rabbit polyclonal).

#### Nuclease reaction

For DNA substrate preparation, oligonucleotides were annealed as for competitor DNA (see above) but to a final concentration of 0.5 μM each. See key resources table for oligonucleotide details.

Nuclease reactions (10 μl) in Figures 5 and S5 were performed at 30°C and 1250 rpm in 100 mM K-glutamate DNA replication buffer. 10 nM annealed DNA substrate and 2.5 mM ATP were incubated with PCNA and RFC (or their buffers) for 5 min at 30°C before addition of the corresponding nuclease and, in Figures 5C, 5D and S5F, also addition of the buffer or exo^−^ Polε. Nuclease reactions were incubated at 30°C for 5 min (Polε), 5 min (ExoIII) or 45 min (MRX). Nuclease reactions with MRX contained 2 mM MnOAc. Reactions were then stopped by the addition of 5 μl 3x STOP buffer to have final concentrations of 30 mM EDTA, 0.5% SDS, 6.5% Ficoll and 0.3 mg/ml Proteinase K, and incubated at 30°C for 10 min before separating (dark) in a 20% native TBE-PAGE (Invitrogen) in 1xTBE running buffer at 150V for 75 min at room temperature, and Cy3 fluorescence was visualised with Amersham Imager 800, GE lifesciences.

#### Cell viability assay

Cells were grown overnight at 30°C in YP + 2% raffinose, diluted in the morning in YP + 2% raffinose to OD 0.1 and grown to OD 0.3 before addition of 20 μg/ml α-factor for 1h. At this point, cells were maintained in α-factor for 1-2 more hours, but culture was divided in two and 2% glucose or 2% galactose was added to each half. A sample of G1-synchronised cells was taken, and cells were washed and released into YP + 2% galactose or 2% galactose + 0.01% Methyl Methanesulfonate (MMS) and 5 μg/ml nocodazole for 1h at 30°C before taking sample of MMS condition. To evaluate cell viability, 100 μl of cells were diluted (1:5000-1:20,000) on YP + 2% glucose and plated in YP + 2% glucose agar plates. Colonies were left to grow for 2-3 days at 30°C and number of colonies/plate/condition were counted. For each strain and condition, we also collected samples for western blot and flow cytometry (see below).

#### Western blot

For protein expression analysis, 1 ml cell culture from viability experiment in Figure 4G was extracted in 85% TCA and lysed for 40 sec at 6 m/s using a Fastprep. Extracts were then processed and separated on 4-15% Tris-Glycine SDS-PAGE, transferred to nitrocellulose, and immunoblotted with anti-PCNA ([5E6/2], mouse monoclonal), anti-Flag (Rfc1) ([M2], mouse monoclonal), anti-Rad53 ([Abcam ab104232], rabbit polyclonal) and anti-PGK ([Abcam 22C5D8], mouse monoclonal).

#### Flow cytometry

For flow cytometric analysis, 200 μl cell culture from viability experiment in Figure 4G was fixed in 70% ethanol for 30 min and then treated overnight with 1 mg/ml RNAse A at 30 °C in 50 mM sodium citrate. Cells were stained with 4 μg/ml propidium iodide in 50 mM sodium citrate and analysed using a FACSCalibur (BD) and FlowJo Software.

### Quantification and Statistical Analysis

DNA replication quantifications were performed using ImageJ software following signal image linearisation with ‘linearise gel data’ plug-in. Distance in the gel was calibrated to base pair length using the λ DNA-HindIII Digest ladder (NEB) with an exponential fit.

Quantification of DNA synthesis in leading and lagging strands in Figure 1B was done by plotting lane signal intensity and calculating the area under the curve for each strand using ImageJ.

Quantification of leading DNA replication length were done using Graph-pad Prism by first fitting leading DNA synthesis intensity to a non-linear gaussian curve and calculating the mean (representing the maximum bulk DNA synthesis for each lane). To calculate DNA synthesis speed the mean of bulk DNA synthesis of each lane was fit to a simple linear regression.

Quantification of nuclease activity in Figure 5 was done by plotting signal intensity of individual lanes in ImageJ.

### Statistical Analysis

Graphpad Prism software was used for all statistical analyses. Statistical methods are described in the figure legends as appropriate.

## Supplementary Material

Supplemental information can be found online at https://doi.org/10.1016/j.molcel.2025.06.001.

Supplementary Information

## Highlights

- Incomplete Okazaki fragments (OkFs) accumulate during dNTP depletion
- Excess OkFs sequester and deplete PCNA, RFC, and DNA Polymerases **ε** and **δ**
- This depletion prevents replication restart and deprotects 3′ ends of nascent DNA
- The checkpoint prevents sequestration and protects forks by limiting DNA unwinding.

## In brief

Canal et al. used biochemical reconstitution of DNA replication to show that continued Okazaki fragment synthesis after nucleotide exhaustion depletes PCNA, RFC, and DNA polymerases **ε** and **δ**, deprotecting replication forks and impairing restart. By limiting Okazaki fragment generation and factor depletion, the replication checkpoint protects forks, allows restart, and promotes survival.

## Resource Availability

### Lead contact

Further information and requests for resources and reagents should be directed to and will be fulfilled by the lead contact, John F.X. Diffley (john.diffley@crick.ac.uk).

### Materials availability

All unique strains and plasmids generated in this study will be made available upon request to the lead contact but may require a completed Materials Transfer Agreement.

This study did not generate new unique reagents.

### Data and code availability

- Original data images have been deposited at Mendeley and are publicly available as of the date of publication at https://doi.org/10.17632/n9dbmtkds7.1.
- This paper does not report original code.
- Any additional information required to reanalyze the data reported in this paper is available from the lead contact upon request.
