# Supplementary material for: The DNA replication checkpoint limits Okazaki fragment accumulation to protect and restart stalled forks
Source: Mol Cell. Author manuscript; Available in PMC 2026 Sep 1. (PMC7619431; doi:10.1016/j.molcel.2025.06.001)
Supplement: Supplementary file 1 [file EMS217827-supplement-Supplemental_Information.pdf]

Molecular Cell, Volume *85*

Supplemental information

The DNA replication checkpoint limits Okazaki fragment accumulation to protect and restart stalled forks

Berta Canal, Agostina P. Bertolin, Giselle C. Lee, Lucy S. Drury, Masashi Minamino, and John F.X. Diffley

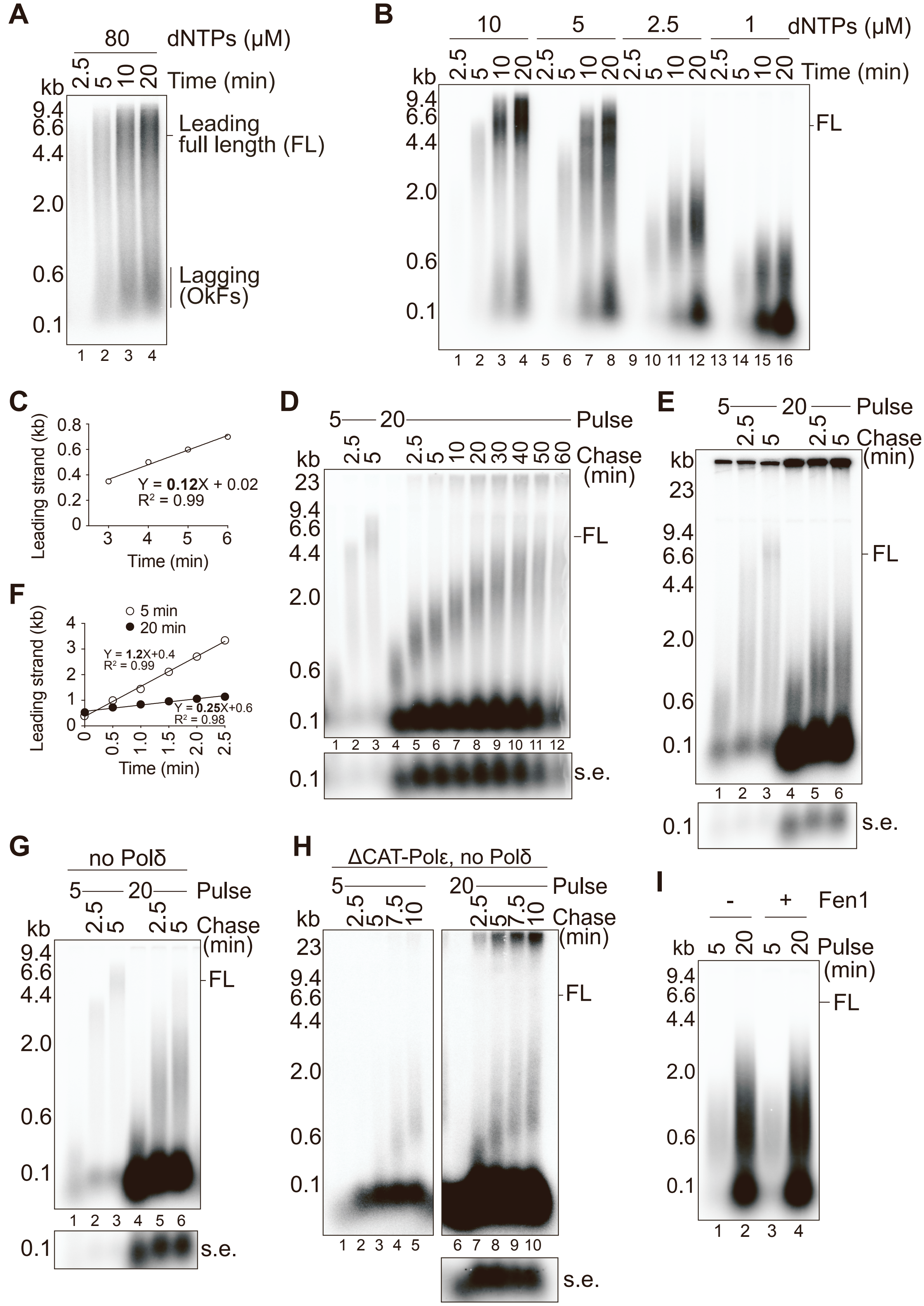

Figure S1. DNA replication fork stalling and restart in vitro, related to Figure 1.

(A) Time course of DNA replication reaction with 80 μM each dNTP.

(B) DNA replication reaction for 2.5, 5, 10 and 20 min in the presence of 10, 5, 2.5 or 1 μM each

dNTP. We chose 1 μM dNTP as our 'low dNTP' concentration as it still allows to visualise and distinguish leading and lagging strands, while leading strand is short enough to evaluate restart.
(C) Quantification of bulk leading strand DNA synthesis speed from 3 to 6 min in **Figure 1A**. Calculated speed of leading strand synthesis at low dNTP concentration is 0.12 kb/min.
(D) Pulse-chase DNA replication reactions. 5 and 20 min low dNTP pulses chased for 2.5 and 5 min, and 20 min pulse further chased every 10 min up to 60 min.
(E) Pulse-chase DNA replication reactions denatured with formaldehyde/formamide (see STAR Methods for details).
(F) Quantification of bulk leading strand DNA synthesis restart after 5 and 20 min low dNTP pulses from **Figure 1E**. Calculated speed of leading strand restart is 1.2 kb/min after 5 min and 0.25 kb/min after 20 min.
(G) Pulse-chase DNA replication reactions omitting Polδ from all reactions.
(H) Pulse-chase DNA replication reactions where Polε was substituted with the non-catalytic mutant (ΔCAT) Polε, and Polδ was omitted. Polα is the only active polymerase in these reactions. Both gel images in this figure were cropped from a common gel and are comparable since they represent the same exposure of the common gel image.
(I) DNA replication at low dNTP concentration for 5 or 20 min in the absence or presence of 200 nM Fen1 nuclease.
(FL) represents Full leading strand Length. (s.e.) represents a shorter exposure of the Okazaki fragments (OkFs).

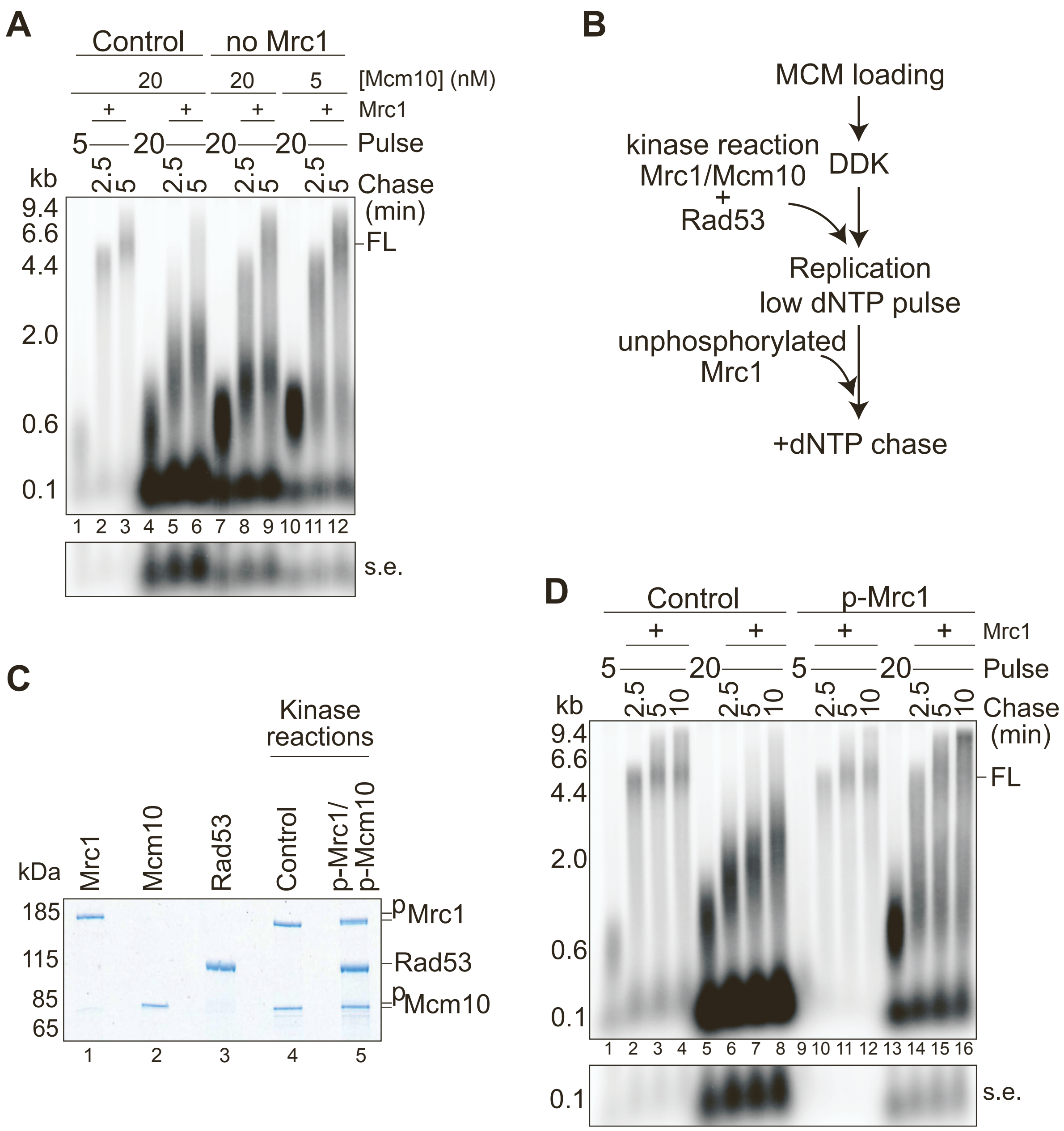

Figure S2. The DNA replication checkpoint protects stalled forks, related to Figure 2.

(A) Pulse-chase DNA replication reactions. Reactions contained 20 nM Mrc1 and Mcm10 (Control) or no Mrc1 and 20nM (standard) or 5nM (low) Mcm10. 30 sec before chasing for 2.5, 5 and 10 min, 20 nM Mrc1 were added to all pulses.
(B) Schematic of the experimental steps in **Figure 2C**. Kinase reactions with Mrc1, Mcm10 and buffer or kinase Rad53 for 30 min were added to DNA replication reactions at low dNTP concentration (pulse) for 5 or 20 min. 30 seconds before adding dNTP chase, unphosphorylated Mrc1 was added to all pulses.
(C) SDS-PAGE and Coomassie staining of individual purified proteins next to 5 µl of the kinase reactions used in **Figure 2C** (Control and p-Mrc1/p-Mcm10).
(D) Pulse-chase DNA replication reactions. Reactions contained Control (unphosphorylated) or Rad53-phosphorylated 20 nM Mrc1 (p-Mrc1). 30 sec before chasing for 2.5, 5 and 10 min, unphosphorylated Mrc1 was added to all pulses.
(FL) represents Full leading strand Length. (s.e.) represents a shorter exposure of the OkFs.

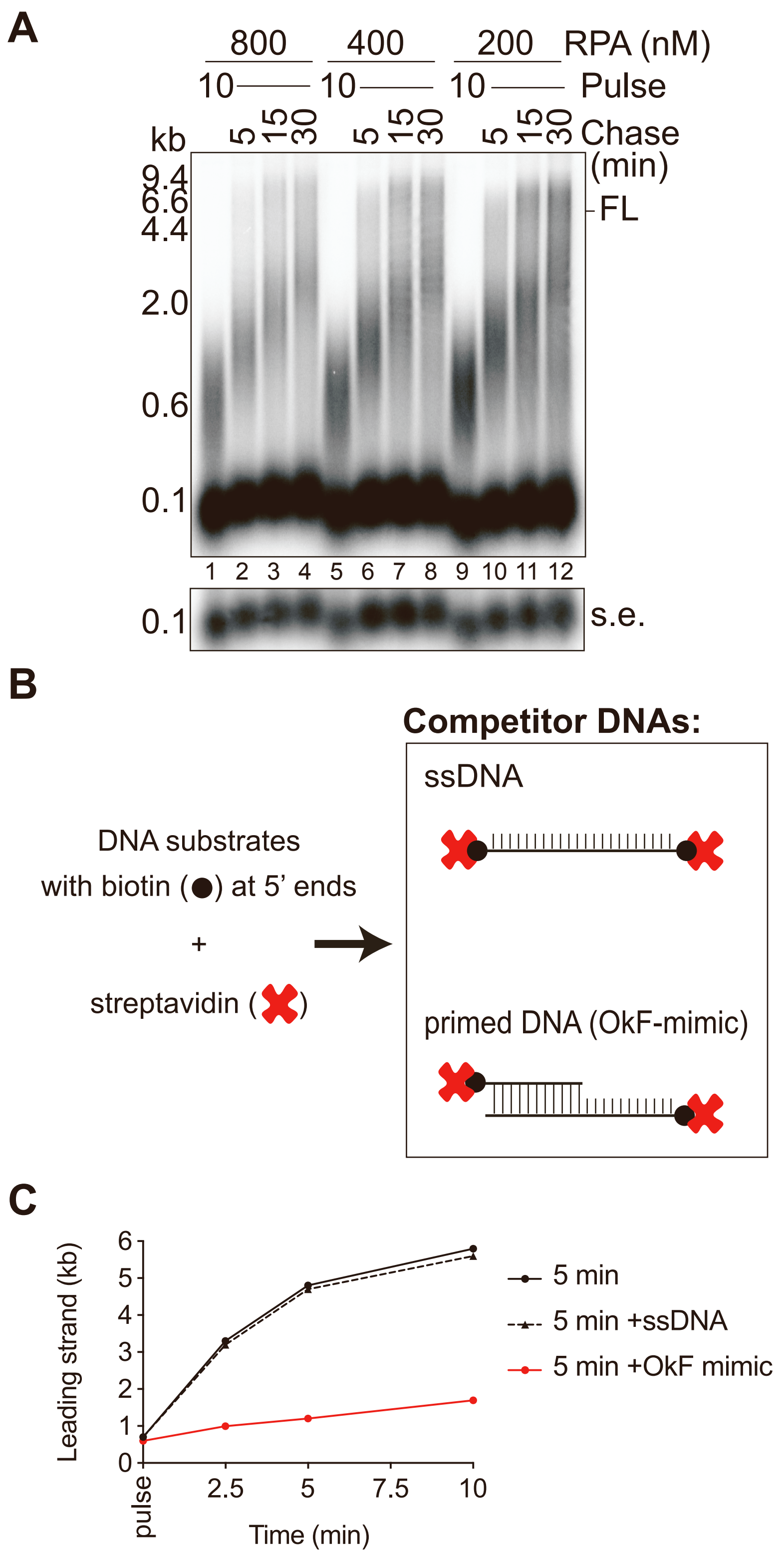

Figure S3. Excess OkF initiation prevents restart, related to Figure 3.

(A) Pulse-chase DNA replication reactions. 10 min low dNTP pulses with 800, 400 or 200 nM RPA, chased each for 5, 15 and 30 min.
(B) Scheme of the competitor ssDNA and competitor primed DNA. DNA oligonucleotides labelled with biotin were incubated with streptavidin to block the ends.
(C) Quantitation of bulk leading strand replication products of experiment in **Figure 3E,** in kilobases (kb).
(FL) represents Full leading strand Length. (s.e.) represents a shorter exposure of the OkFs.

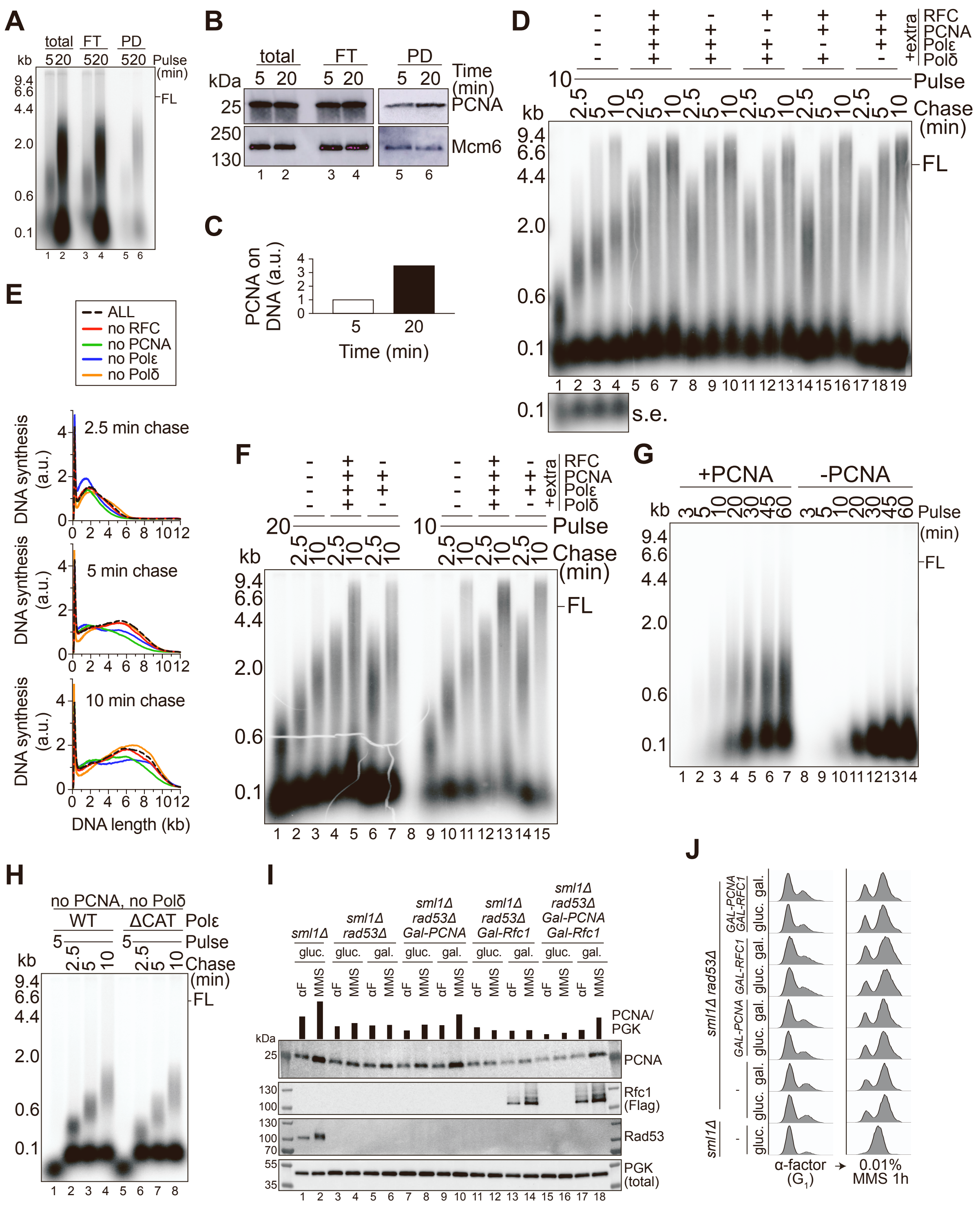

Figure S4. Depletion of PrSM factors contributes to fork stalling and causes aberrant restart, related to Figure 4.

(A) DNA replication in low dNTPs and biotinylated dUTP and immunoprecipitation of replicated DNA. DNA replication in total, flow through (FT), and pull down (PD) samples of experiment in **Figure S4C**.
(B) Western blot of PCNA and Mcm6 proteins in total, FT, and PD samples of the immunoprecipitation of replicated DNA in **Figure S4C**.
(C) Quantification of PCNA co-immunoprecipitated with replicated DNA for 20 min at low dNTPs. Graph represents immunoprecipitated PCNA levels at 20 min relative to levels at 5 min, each normalised to the Mcm6 levels representing the total loading control.

(D) Pulse-chase DNA replications. 10 min pulse. 30 sec before chasing, extra individual PrSM factor DROP-OUT combinations were added as in **Figure 4C**.
(E) Lane profiles of the data in (**D**) are shown. Graphs represent amount of DNA synthesis (arbitrary units) vs length of restart (kilobases) at 2.5, 5 and 10 min chase.
(F) Pulse-chase DNA replications. 20 and 10 min pulses. 30 sec before chasing, buffers, 250 nM PCNA, 125 nM RFC, 100 nM Polε and 10 nM Polδ or only 250 nM PCNA and 100 nM Polε were added.
(G) DNA replication at low dNTP concentration in the presence or absence of 25 nM PCNA.
(H) Pulse-chase DNA replication reactions with WT or catalytically dead ΔCAT-Polε. PCNA and Polδ were omitted. In lanes 5 to 8, Polα is the only active DNA polymerase present in the reaction.
(I) Western blot of whole cell protein extracts from experiment in **Figure 4G**. Shown are PCNA, Rfc1 (Flag), Rad53 and PGK (total).
(J) Flow cytometry analysis of samples from experiment in **Figure 4G**. Each exponentially growing strain was synchronised in α-factor (left column) and then released for 1h into medium containing 0.01% MMS (right column) in the specified yeast extract peptone (YP) media supplemented with 2% glucose (gluc.) or galactose (gal.).
(FL) represents Full leading strand Length. (s.e.) represents a shorter exposure of the OkFs.

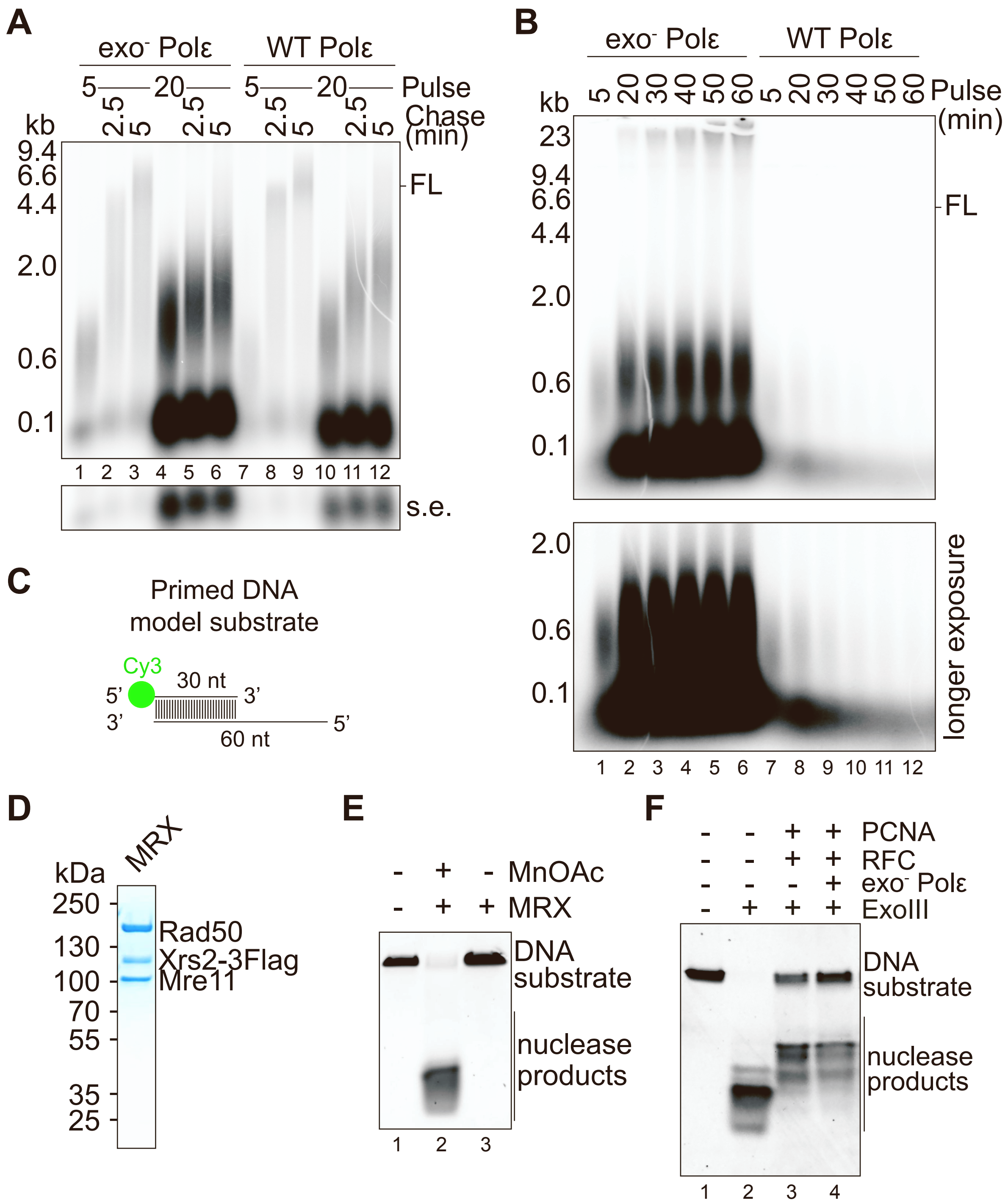

Figure S5. PrSM factors protect nascent DNA from nuclease attack, related to Figure 5.

(A) Pulse-chase DNA replication reactions. 5 and 20 min low dNTP pulses chased each for 2.5 and 5 min, containing either exonuclease mutant (exo$^-$) or WT Polε.
(B) Time course of DNA replication reactions up to 60 min always in low dNTP concentrations containing either exo$^-$ or WT Polε.
(C) Scheme of the primed DNA model substrate used in nuclease reactions. 30 nt oligonucleotide labelled with fluorescent Cy3 at 5' is annealed to a 60 nt oligonucleotide creating a 3' ds/ssDNA junction.
(D) Purified recombinant budding yeast Mre11, Rad50, Xrs2 (MRX) complex analysed by SDS–PAGE with Coomassie staining.

(E) Nuclease reactions with MRX nuclease in the presence or absence of 2 mM manganese acetate (MnOAc) to confirm specificity of nuclease activity of purified recombinant MRX complex separated in TBE-PAGE and imaged for Cy3 fluorescence.
(F) Nuclease reactions performed for 15 min with 2U ExoIII nuclease in the absence or presence of 250 nM PCNA, 125 nM RFC and 100 nM exo^{-} Polε separated in TBE-PAGE and imaged for Cy3 fluorescence. Longer reaction times unravel a role for exo^{-} Polε also in protection of DNA ends from nucleolytic attack.
(FL) represents Full leading strand Length. (s.e.) represents a shorter exposure of the OkFs.

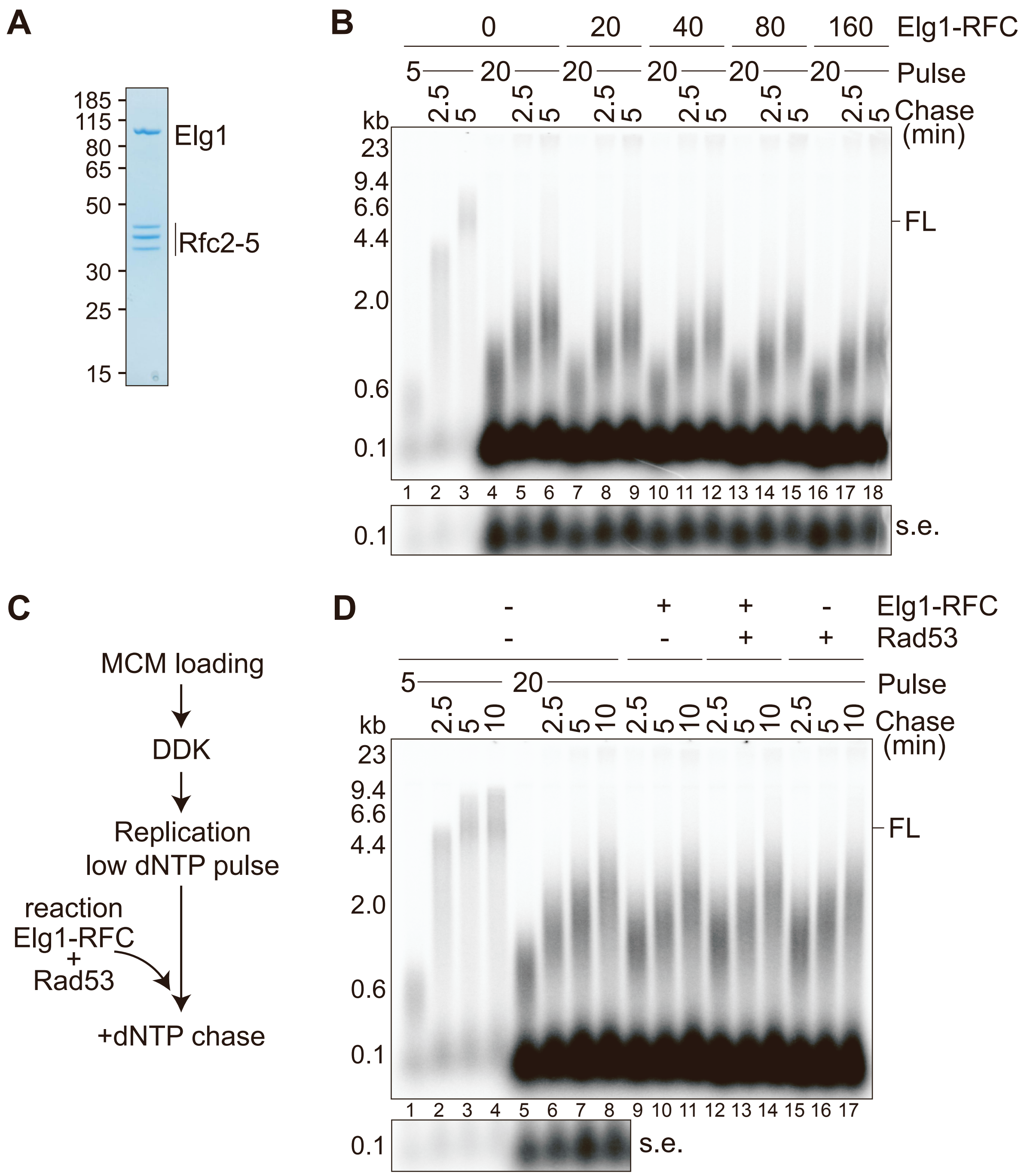

Figure S6. Elg1-Rfc2-5 is not sufficient to suppress PrSM depletion, related to Figure 4.

(A) Purified recombinant budding yeast Elg1-Rfc2-5 complex by SDS–PAGE with Coomassie staining.
(B) Pulse-chase DNA replication reactions. 5 or 20 min low dNTP pulses with 0, 20, 40, 80, 160 nM Elg1-Rfc2-5, chased each for 2.5 and 5 min.
(C) Schematic of the experimental steps in (**D**).
(D) Pulse-chase DNA replication reactions. 5 or 20 min low dNTP pulses. Kinase reactions containing combinations of Elg1-Rfc2-5 and Rad53 or their buffers (see Rad53 kinase reaction in STAR Methods for details) were added to each pulse 30 sec before chasing with dNTPs for 2.5, 5 and 10 min.
(FL) represents Full leading strand Length. (s.e.) represents a shorter exposure of the OkFs.

Supplemental Tables S1 – S3

Table S1. Yeast strains generated in this study, related to STAR Methods.	
**Name**	**Genotype**
yBC107 (expression MRX)	*yJF1 + pBC42 (NheI)::HIS3 + pBC43 (StuI)::URA3*
yBC155 (expression Elg1-Rfc2-5)	*MATa ade2-1 ura3-1 his3-11,15 trp1-1 leu2-3,112 can1-100*<br>*bar1::Hyg*<br>*pep4::KanMX*<br>*ura3::URA3 pRS306-RFC2+CBP-RFC3*<br>*trp1::TRP1 pRS304-RFC4 + RFC5*<br>*his3::HIS3 pRS303-ELG1-3xFlag (pBC52 (NheI)::HIS3)*
yBC319 (sml1)	*W303-1a sml1Δ::KanMX*
yBC320 (rad53)	*W303-1a sml1Δ::KanMX rad53Δ::LEU2*
yBC296 (rad53 OE PCNA)	*W303-1a sml1Δ::KanMX rad53Δ::LEU2 + pBC65 (StuI)::URA3*
yBC297 (rad53 OE Rfc1)	*W303-1a sml1Δ::KanMX rad53Δ::LEU2 + pBC66 (BsgI)::TRP1*
yBC298 (rad53 OE PCNA/Rfc1)	*W303-1a sml1Δ::KanMX rad53Δ::LEU2 + pBC65 (StuI)::URA3 + pBC66 (BsgI)::TRP1*

Table S2. Plasmids generated in this study, related to STAR Methods.

1. pBC42 (Plasmid pRS303 containing Rad50 gene codon optimised for expression in *S. cerevisiae* under the control of the GAL1,10 promoter. (Gal1,10-Rad50, Gal4 / pRS303))

Atatctctaaattcgcgcgtttcggtgatgacggtgaaaacctctgacacatgcagctcccggagacggtcacagcttgtctgtaagcggatgccgggagcagacaagcccgtcagggcgcgtcagcgggtgttggcgggtgtcggggctggcttaactatgcggcatcagagcagattgt
actgagagtgcaccatatcgacggttttaagagctggtgagcgctaggagtcactgccaggtatcgtttgaacacggcattagtcagggaagtcataacacagtcctttcccgcaattttctttttctattactcttggcctcctctagtacactctatatttttttatgcctcggtaatgatttcattttttttttcc
acctagcggatgactcttttttttttcttagcgattggcattatcacataatgaattatacattatataaagtaatgtgatttcttcgaagaatatactaaaaaatgagcaggcaagataaacgaaggcaaagatgacagagcagaaagccctagtaaagcgtattacaaatgaaaccaagattca
gattgcgatctctttaaagggtggtcccctagcgatagagcactcgatcttcccagaaaaagaggcagaagcagtagcagaacaggccacacaatcgcaagtgattaacgtccacacaggtatagggtttctggaccatatgatacatgctctggccaagcattccggctggtcgctaatcg
ttgagtgcattggtgacttacacatagacgaccatcacaccactgaagactgcgggattgctctcggtcaagcttttaaagaggccctactggcgcgtggagtaaaaaggtttggatcaggatttgcgcctttggatgaggcactttccagagcggtggtagatctttcgaacaggccgtacgca
gttgtcgaactggtttgcaaagggagaaagtaggagatctctcttgcgagatgatcccgcattttcttgaaagctttgcagaggctagcagaattaccctccacgttgattgtctgcgaggcaagaatgatcatcaccgtagtgagagtgcgttcaaggctcttgcggttgccataagagaagcc
acctcgcccaatggtaccaacgatgttccctccaccaaaggtgttcttatgtagtgacaccgattatttaaagctgcagcatacgatatatatacatgtgtatatatgtatacctatgaatgtcagtaagtatgtatacgaacagtatgatactgaagatgacaaggtaatgcatcattctatacgtgtc
attctgaacgaggcgcgctttccttttttcttttttgcttttttcttttttttctcttgaactcgacggatcatatgcggtgtgaaataccgcacagatgcgtaaggagaaaataccgcatcaggaaattgtaaacgttaatattttgttaaaattcgcgttaaatttttgttaaatcagctcattttttaaccaataggc
cgaaatcggcaaaatcccttataaatcaaaagaatagaccgagatagggttgagtgttgttccagtttggaacaagagtccactattaaagaacgtggactccaacgtcaaagggcgaaaaaccgtctatcagggcgatggcccactacgtgaaccatcaccctaatcaagttttttggggt
cgaggtgccgtaaagcactaaatcggaaccctaaagggagcccccgatttagagcttgacggggaaagccggcgaacgtggcgagaaaggaagggaagaaagcgaaaggagcgggcgctagggcgctggcaagtgtagcggtcacgctgcgcgtaaccaccacacccgccg
cgcttaatgcgccgctacagggcgcgtcgcgccattcgccattcaggctgcgcaactgttgggaagggcgatcggtgcgggcctcttcgctattacgccagctggcgaaggggggatgtgctgcaaggcgattaagttgggtaacgccagggttttcccagtcacgacgttgtaaaacgac
ggccagtgaattgtaatacgactcactatagggcgaattggagctccaccgcggtggcggccgcGCATAAAGGCATTAAAAGAGGAGCGAATTTTTTTTTAATAAAAATCTTAATAATCATTAAAAGATAAATAATAGTCTATATATAC
GTATATAAATAAAAAATATTCAAAAAATAAAATAAACTATTATTTTAGCGTAAAGGATGGGGAAAGAGAAAAGAAAAAAATTGATCTATCGATTTCAATTCAATTCAATTTAGTAAGTGACTCTGTTGATG
TCGACCCATTCAATTTGGGACTTTTGTCTATCATCTCTCTTGACCTTGAAGAAGTGATCAGTGAAAGCAGCAGCGTTCATGTGACCCAAGAACTTTTCGTCGTGAGTAATGACAATCAATTGGAAGTT
CTTTTGGTGTCTTCTCATGTTGATAATGTTGTGCAAAGACTTAGCCAAAGATTCGATGTTTTCTTCGTCCAAGTTAGTGGTTGGTTCGTCCAAAGCAATGACACCACAGTTAGCACCGAAGGTTTCAG
ACAAAGCCAATCTAATGATAATAGAAGCCAAGACCTTTTGACCAGCAGAACATCTACCTCTCATATCCAATTCGACATCTTGCTTGTACATGACAACTCTGTAGTTGTAAGACTTACCCTTGACAGTA
GAAGAAACTTCATCGGATCTAATCTTAATAGTGTCAATGTCAGTACCAGAGTAAGTTCTCTTCCACAATTCGTCGATGATTCTGTTGATGTCTTGCATCTTCAAACCGTGGTACTTCATGATAGCAGAA
TCCAAAGCCTTAGAGTAGACGTCAATATCGTCAGTGACGAAAGATCTGGTTTGCAATTCAACCCATTCCTTGTGGTAGTTCTTTTCGATGTCCTTGTAGTCAGTTCTCAATTGGTGAGTCAAGGAGTC
AATTTGGTTTTGCAATTGCTTCATTTCACCCAACTTACCAGCGTTTTCAGAGGACAACTTTTCGAATCTAGTTCTCAATCTCAAAGATTCTTCTTGGTACTTGTCTCTTTCAGCTTCAGCGTTTTGAACG
TCCAATCTAGAGATTTCGGATTCAATGTGTTGCAATTGAGACTTCAATTCAATCAATTCCAAGTTTTGCTTCAAGTTCTTTTCTTCGTTGTTAGAGTCAGCCAACTTTCTCTTTTCTTCGTTGACTTCGTT
AGACTTCAAGTCCAATTGTTCCTTCAATTCCAACATTTGAGCCTTGTTCAATTCCAATTCCTTGATAGTGGTTTGCAATTCATCGAAACCCTTAGCTTCGAAATCAACAACTTCGTTGTAAATAGTTTGG
AATCTGTCGATCAATCTGTTGATGTCAGCAACGGTCTTTTGCTTGTTTCTGACTTGAATATCTCTTTCGTTCTTAACCTTGTCCAAGACGGATTGAGCTTCATCCTTCTTGTTCTTCAAGGAGATGATT
CTAGCTTCCAATTCCTTAACTCTAGAGTCGATATCGTTGATGTTTTCTCTCTTGGATCTGATAGAGTCATCGATGTTTTGCTTTTGGGTCAAGGAAGATTCAATTTCGGAAACAGTCAATTCCTTTTCCT
TAATCAAGTTGATCATTCTGGAGTTTTCTCTAACCTTTTCGTCCTTTTCCATTTGCAAATCAGAAATGGTCTTTCTCAATTCTCTCAAAGAATCGTTCATCTTTCTTTGTTGGTCTCTCAATTCGTCGACG
GTTTGGATACCGTCTTCAGAAGTGTTGTAGATGGACAATTCTTCGGAAATAGTCTTGGAGGAGTTTTCCAAGTCCTTCAATTCCTTTTCCAAGTAGGTGAACTTTTCGATCAATGGTCTAATTTCAGAT
TCAGCCAATTCCTTTTCGTCCTTCAACTTGGTGGAGTCGACTTCCAATTCATCCAACTTGGACTTAGAAGTCTTGGTTTCTTCCTTAGCCTTTTCCAAACACTTTTGAGAGTTGTCGATCTTTTCGTTG
ATGGAGTTCAAAGTAATAATGTGCTTTTCCAACAATCTCAAGGAGTGCAAGTATTCCTTTTCGTTTTGGACAGTATCCTTCATCAAAGTCTTTTCGAAGTTAGCGTCAGTCTTAGTCTTCAATTCTTGCAAC
AACTTGGACTTGAAAGATTCGTTTTCGAACTTTCTAGAACACAAGTAACAACAAGAATCTCTTTCAGCAATTTCCAAAGCCTTTCTGTTGAATTCCAAAGTAGTTTGGTGCATCTTCAAGTTTTCCAAA
GCGGTCTTGTAGGACAATTCGGTTTCTTCCAAAACGTCGTTGTATTCATCAATGGTACAGTCTTCTGGCAAGTTTTCGGACAACAATTGAATAACCTTTCTTCCTTAGACTTTGGTTATCTTGCAAATCC
TTTTCGATGGTGTTCAAGTTGTACAAAGCGTTGGTGTATCTTCTGTCCAATTCGTGCATCTTCTTGTTGTTAATAGCAATGTTCTTTTGCATGTTAATGAACAACTTTTGGAAGTCCATTTCCAAGTCAG
CTCTTTGGAATTCTTGGGTCAATGGGAAAACTTGTCTGATTCTAGAGTCGTTTTGCAACTTTTCAGTGATCTTTTGCAATTCATCCAACTTAGTGTTGATAGACTTCTTGATCAAACCCAACTTAGCGT
ACAAGTCAGCTTGTTGGTTAGTCTTCATAATTCTATCTTGGAACTTTTCGATTTGGTTTTCCAAAATGATCATTTCGTTGTTCTTTTCTTCGATCTTTTGGTTCAACTTTGGAATAATGTTTTCGGATTCC
CAGGATTGCAACTTTTCCTTGTAAGTCTTCAAGTTTTCCAATTCGTGGTTCAAAGAGTCTTGAGTGGACAAAGACTTGAAGGACTTCAACTTTTCAGCCAATTCTTCAGAGTCGTGAATCAACTTAGAT
CTATCCTTCTTGTTGTATTCCAAGTTTTGAGAATCAACGGTGATGGACTTAATCAAGTCAGACAAGTTAGTTTCCTTCAATTGGATGTCCTTAGCGAATTGGTCGATAGTGTCGGTCAAGTCTTGAGA
AATGAAAGCCTTGAATTGGGACATTTCGTGGTTAACTTGAGCCATATCAGAGTTTTCAATGTTAGACAAACCTTGGAACTTGTGTTGGAAAGCTTCCTTCAAAGAGGACAAGTGGTTTCTGTTCTTTT
CGTAGGTTTCCTTACCAGCTTCCAATTCAACTTCACCTTGTCTTCTAATCAAAGAGTTAGACAAGGATTGCAAAGAAGATTGTCTATCCTTCAAAGAAGAGATATCGGTTTCCAAGTCTCTCAATTGGTTGTTCT
TATCCATCAAGACCTTAGAGAAGTTAGCCAACAAGTTTTGCAAGTCTGGCTTAGACAAATCCAAGATATCGATGGAGTTGGACAATCTCTTGACTTGATCGGAGATAGACAACTTGGTGTTCTTCAAG
TTTTCGACCTTAGACAAAATCTTTTGGAAGTCTTGGTTAGACTTGAACAACTTGTCAGACTTTTCAGTAATTTCGTTCAATTGAGATTCGATTTCAGAGACTTCTTCGTTGTATTGGTCGATCTTAGTTT
GCAATTGGTGAATGTTCAACTTCATAGCCTTAGATCTGTCCTTATCCAACTTCAAGTGTTCAACGGATTGCTTCAACAACTTGATATCGACAGACATGTCCTTCTTAATAGACTTCAAGTTGTCCAAAG
CCTTGGTGAACTTCATAGCTTGGAAGATTCGTCGAACTTCTTCTTCAAGTTAGATGGTCAGACAATGGCCACAAAGAGTCTTCTTGGTGACAGAAGATAACGTATTCCAAGATAGCCTTTGGGACA
CCCAAGTACAATGGAACTTGAGCGTCCAATTCCAAGGATCTAGTGGACAAGGTAGATCTATCACCAGAGTTGTTAATAGCGACCAATTGACCTTCCAAAGTCTTGAAAGTGGTAGTAGTCTTCTTCAT
CAACAATTGAATGTTTCTGGTGACAATCATGTTCAAACCGTTAGCAGAAGTGAAAGCCAACTTGACTTGAGCTCTAATATCCTTTTCACCAGTAATCTTTGGGTCGTGGATGAAGACACCACCCTTGG
AGTTTGGTGGCAAGTCACCAGTAGTAGCGTACTTCAAACATTCAATGATAGTAGTCTTACCAGAACCGTTCATACCAACAATCAAGGTCAATGGCTTACCGAATTCAATAGTTTCTCTATCGTTGGAA
TCGAAGGATCTAATACCTTGAATAGACAACTTGTAAATAGCAGACATTGTTTTATACACCGGTGTATAGTTTTTTCTCCTTGACGTTAAAGTATAGAGGTATATTAACAATTTTTTGTTGATACTTTTATG
ACATTTGAATAAGAAGTAATACAAACTGAAAATGTTGAAAGTATTAGTTAAAGTGGTTATGCAGCTTTTCCATTTATATATCTGTTAATAGATCAAAAATCATCGCTTCGCTGATTAATTACCCCAGAAA
TAAGGCTAAAAAACTAATCGCATTATCATCCTATGGTTGTTAATTTGATTCGTTAATTTGAAGGTTTGTGGGGCCAGGTTACTGCCAATTTTTCCTCTTCATAACCATAAAAGCTAGTATTGTAGAATCT
TTATTGTTCGGAGCAGTGCGGCGCGAGGCACATCTGCGTTTCAGGAACGCGACCGGTGAAGACGAGGACGCACGGAGGAGAGTCTTCCGTCGGAGGGCTGTCGCCCGCTCGGCGGCTTCTAAT
CCGTACTTCAATATAGCAATGAGCAGTTAAGCGTATTACTGAAAGTTCCAAAGAGAAGGTTTTTTTAGGCTAAGATAATGGGGCTCTTTACATTTCCACAACATATAAGTAAGATTAGATATGGATATG
TATATGGTGGTAATGCCATGTAATATGATTATTAAACTTCTTTGCGTCCATCCAAAAAAAAAGTAAGAATTTTTGAAAATTCAATATAAGGCGCGCCATGAAGCTACTGTCTTCTATCGAACAAGCATG
CGATATTTGCCGACTTAAAAAGCTCAAGTGCTCCAAAGAAAAACCGAAGTGCGCCAAGTGTCTGAAGAACAACTGGGAGTGTCGCTACTCTCCCAAAACCAAAAGGTCTCCGCTGACTAGGGCACA
TCTGACAGAAGTGGAATCAAGGCTAGAAAGACTGGAACAGCTATTTCTACTGATTTTTCCTCGAGAAGACCTTGACATGATTTTGAAAATGGATTCTTTACAGGATATAAAAGCATTGTTAACAGGATT
ATTTGTACAAGATAATGTGAATAAAGATGCCGTCACAGATAGATTGGCTTCAGTGGAGACTGATATGCCTCTAACATTGAGACAGCATAGAATAAGTGCGACATCATCATCGGAAGAGAGTAGTAAC
AAAGGTCAAAGACAGTTGACTGTATCGATTGACTCGGCAGCTCATCATGATAACTCCACAATTCCGTTGGATTTTATGCCCAGGGATGCTCTTCATGGATTTGATTGGTCTGAAGAGGATGACATGT
CGGATGGCTTGCCCTTCCTGAAAACGGACCCCAACAATAATGGGTTCTTTGGCGACGGTTCTCTCTTATGTATTCTTCGATCTATTGGCTTTAAACCGGAAAATTACACGAACTCTAACGTTAACAGG
CTCCCGACCATGATTACGGATAGATACACGTTGGCTTCTAGATCCACAACATCCCGTTTACTTCAAAGTTATCTCAATAATTTTCACCCTCACTGCCCTATCGTGCACTCACCGACGCTAATGATGTT
GTATAATAACCAGATTGAAATCGCGTCGAAGGATCAATGGCAAATCCTTTTTAACTGCATATTAGCCATTGGAGCCTGGTGTATAGAGGGGGAATCTACTGATATAGATGTTTTTTACTATCAAAATG*
CTAAATCTCATTTGACGAGCAAGGTCTTCGAGTCAGGTTCCATAATTTTGGTGACAGCCCTACATCTTCTGTCGCGATATACACAGTGGAGGCAGAAAACAAATACTAGCTATAATTTTCACAGCTTT
TCCATAAGAATGGCCATATCATTGGGCTTGAATAGGGACCTCCCCTCGTCCTTCAGTGATAGCAGCATTCTGGAACAAAGACGCCGAATTTGGTGGTCTGTCTACTCTTGGGAGATCCAATTGTCCC
TGCTTTATGGTCGATCCATCCAGCTTTCTCAGAATACAATCTCCTTCCCTTCTTCTGTCGACGATGTGCAGCGTACCACAACAGGTCCCACCATATATCATGGCATCATTGAAACAGCAAGGCTCTTA
CAAGTTTTCACAAAAATCTATGAACTAGACAAAACAGTAACTGCAGAAAAAAGTCCTATATGTGCAAAAAAATGCTTGATGATTTGTAATGAGATTGAGGAGGTTTCGAGACAGGCACCAAAGTTTTTA
CAAATGGATATTTCCACCACCGCTCTAACCAATTTGTTGAAGGAACACCCTTGGCTATCCTTTACAAGATTCGAACTGAAGTGGAAACAGTTGTCTCTTATCATTTATGTATTAAGAGATTTTTTCACT
AATTTTACCCAGAAAAAGTCACAACTAGAACAGGATCAAAATGATCATCAAAGTTATGAAGTTAAACGATGCTCCATCATGTTAAGCGATGCAGCACAAAGAACTGTTATGTCTGTAAGTAGCTATAT
GGACAATCATAATGTCACCCCATATTTTGCCTGGAATTGTTCTTATTACTTGTTCAATGCAGTCCTAGTACCCATAAAGACTCTACTCTCAAACTCAAAATCGAATGCTGAGAATAACGAGACCGCACA
ATTATTACAACAAATTAACACTGTTCTGATGCTATTAAAAAAACTGGCCACTTTTAAAATCCAGACTTGTGAAAAATACATTCAAGTACTGGAAGAGGTATGTGCGCCGTTTCTGTTATCACAGTGTGC
AATCCCATTACCGCATATCAGTTATAACAATAGTAATGGTAGCGCCATTAAAAATATTGTCGGTTCTGCAACTATCGCCCAATACCCTACTCTTCCGGAGGAAAATGTCAACAATATCAGTGTTAAATA
TGTTTCTCCTGGCTCAGTAGGGCCTTCACCTGTGCCATTGAAATCAGGAGCAAGTTTCAGTGATCTAGTCAAGCTGTTATCTAACCGTCCACCCTCTCGTAACTCTCCAGTGACAATACCAAGAAGC
ACACCTTCGCATCGCTCAGTCACGCCTTTTCTAGGGCAACAGCAACAGCTGCAATCATTAGTGCCACTGACCCCGTCTGCTTTGTTTGGTGGCGCCAATTTTAATCAAAGTGGGAATATTGCTGATA
GCTCATTGTCCTTCACTTTCACTAACAGTAGCAACGGTCCGAACCTCATAACAACTCAAACAAATTCTCAAGCGCTTTCACAACCAATTGCCTCCTCTAACGTTCATGATAACTTCATGAATAATGAAA
TCACGGCTAGTAAAATTGATGATGGTAATAATTCAAAACCACTGTCACCTGGTTGGACGGACCAAACTGCGTATAACGCGTTTGGAATCACTACAGGGATGTTTAATACCACTACAATGGATGATGTA
TATAACTATCTATTCGATGATGAAGATACCCCACCAAACCCAAAAAAAGAGTAAAGCcccggctgcaggaattcgatatcaagcttatcgataccgtcgacctcgaggggggggcccggtacccagcttttgttccctttagtgagggttaattccgagcttg
gcgtaatcatggtcatagctgtttcctgtgtgaaattgttatccgctcacaattccacacaacataggagccggaagcataaagtgtaaagcctggggtgcctaatgagtgaggtaactcacattaattgcgttgcgctcactgcccgctttccagtcgggaaacctgtcgtgccagctgcattaat
gaatcggccaacgcgcggggagaggcggtttgcgtattgggcgctcttccgcttcctcgctcactgactcgctgcgctcggtcgttcggctgcggcgagcggtatcagctcactcaaaggcggtaatacggttatccacagaatcaggggataacgcaggaaagaacatgtgagcaaaag
gccagcaaaaggccaggaaccgtaaaaaggccgcgttgctggcgtttttccataggctcggcccccctgacgagcatcacaaaaatcgacgctcaagtcagaggtggcgaaacccgacaggactataaagataccaggcgtttccccctggaagctccctcgtgcgctctcctgttccg
accctgccgcttaccggatacctgtccgcctttctcccttcgggaagcgtggcgctttctcaatgctcacgctgtaggtatctcagttcggtgtaggtcgttcgctccaagctgggctgtgtgcacgaaccccccgttcagcccgaccgctgcgccttatccggtaactatcgtcttgagtccaacccg
gtaagacacgacttatcgccactggcagcagccactggtaacaggattagcagagcgaggtatgtaggcggtgctacagagttcttgaagtggtggcctaactacggctacactagaaggacagtatttggtatctgcgctctgctgaagccagttaccttcggaaaaagagttggtagctctt
gatccggcaaacaaaccaccgctggtagcggtggtttttttgtttgcaagcagcagattacgcgcagaaaaaaaggatctcaagaagatcctttgatcttttctacggggtctgacgctcagtggaacgaaaactcacgttaagggattttggtcatgagattatcaaaaaggatcttcacctag
atccttttaaattaaaaatgaagttttaaatcaatctaaagtatatatgagtaaacttggtctgacagttaccaatgcttaatcagtgaggcacctatctcagcgatctgtctatttcgttcatccatagttgcctgactgccccgtcgtgtagataactacgatacgggagggcttaccatctggccccagt
gctgcaatgataccgcgagacccacgctcaccggctccagatttatcagcaataaaccagccagccggaagggccgagcgcagaagtggtcctgcaactttatccgcctccatccagtctattaattgttgccgggaagctagagtaagtagttcgccagttaatagtttgcgcaacgttgttg
ccattgctacaggcatcgtggtgtcacgctcgtcgtttggtatggcttcattcagctccggttcccaacgatcaaggcgagttacatgatcccccatgttgtgaaaaaaagcggttagctccttcggtcctccgatcgttgtcagaagtaagttggccgcagtgttatcactcatggttatggcagcact
gcataattctcttactgtcatgccatccgtaagatgcttttctgtgactggtgagtactcaaccaagtcattctgagaatagtgtatgcggcgaccgagttgctcttgcccggcgtcaatacgggataataccgcgccacatagcagaactttaaaagtgctcatcattggaaaacgttcttcggggc
gaaaactctcaaggatcttaccgctgttgagatccagttcgatgtaacccactcgtgcacccaactgatcttcagcatcttttactttcaccagcgtttctgggtgagcaaaaacaggaaggcaaaatgccgcaaaaaagggaataagggcgacacggaaatgttgaatactcatactcttcctt
tttcaatattattgaagcatttatcagggttattgtctcatgagcggatacatatttgaatgtatttagaaaaataaacaaataggggttccgcgcacatttccccgaaaagtgccacctgacgtcttattatcatgacattaacctataaaaataggcgtatcacgaggccctttcgtc

2. pBC43 (Plasmid pRS306 containing Mre11 and Xrs2-TEV-3xFlag genes codon optimised for expression in *S. cerevisiae* under the GAL1,10 promoter. (Gal1,10-Mre11, Xrs2-TEV-3xFlag / pRS306))

gacgaaagggcctcgtgatacgcctatttttataggttaatgtcatgataataatggtttcttagacgtcaggtggcacttttcggggaaatgtgcgcggaacccctatttgtttatttttctaaatacattcaaatatgtatccgctcatgagacaataaccctgataaatgcttcaataatattgaaaaa
ggaagagtatgagtattcaacatttccgtgtcgcccttattcccttttttgcggcattttgccttcctgtttttgctcacccagaaacgctggtgaaagtaaaagatgctgaagatcagttgggtgcacgagtgggttacatcgaactggatctcaacagcggtaagatccttgagagttttcgccccga
agaacgttttccaatgatgagcacttttaaagttctgctatgtggcgcggtattatcccgtattgacgccgggcaagagcaactcggtcgccgcatacactattctcagaatgacttggttgagtactcaccagtcacagaaaagcatcttacggatggcatgacagtaagagaattatgcagtgc
tgccataaccatgagtgataacactgcggccaacttacttctgacaacgatcggaggaccgaaggagctaaccgcttttttcacaacatgggggatcatgtaactcgccttgatcgttgggaaccggagctgaatgaagccataccaaacgacgagcgtgacaccacgatgcctgtagca
atggcaacaacgttgcgcaaactattaactggcgaactacttactctagcttcccggcaacaattaatagactggatggaggcggataaagttgcaggaccacttctgcgctcggcccttccggctggctggtttattgctgataaatctggagccggtgagcgtgggtctcgcggtatcattgca
gcactggggccagatggtaagccctcccgtatcgtagttatctacacgacgggcagtcaggcaactatggatgaacgaaatagacagatcgctgagataggtgcctcactgattaagcattggtaactgtcagaccaagtttactcatatatactttagattgatttaaaacttcatttttaatttaa
aaggatctaggtgaagatcctttttgataatctcatgaccaaaatcccttaacgtgagttttcgttccactgagcgtcagaccccgtagaaaagatcaaaggatcttcttgagatcctttttttctgcgcgtaatctgctgcttgcaaacaaaaaaaccaccgctaccagcggtggtttgtttgccggat
caagagctaccaactctttttccgaaggtaactggcttcagcagagcgcagataccaaatactgtccttctagtgtagccgtagttaggccaccacttcaagaactctgtagcaccgcctacatacctcgctctgctaatcctgttaccagtggctgctgccagtggcgataagtcgtgtcttaccg
ggttggactcaagacgatagttaccggataaggcgcagcggtcgggctgaacggggggttcgtgcacacagcccagcttggagcgaacgacctacaccgaactgagatacctacagcgtgagcattgagaaagcgccacgcttcccgaagggagaaaggcggacaggtatccggt
aagcggcagggtcggaacaggagagcgcacgagggagcttccagggggaaacgcctggtatctttatagtcctgtcgggtttcgccacctctgacttgagcgtcgatttttgtgatgctcgtcaggggggcggagcctatggaaaaacgccagcaacgcggcctttttacggttcctggcctttt
tgctggccttttgctcacatgttctttcctgcgttatcccctgattctgtggataaccgtattaccgcctttgagtgagctgataccgctcgccgcagccgaacgaccgagcgcagcgagtcagtgagcgaggaagcggaagagcgcccaatacgcaaaccgcctctccccgcgcgttggccga
ttcattaatgcagctggcacgacaggtttcccgactggaaagcgggcagtgagcgcaacgcaattaatgtgagttacctcactcattaggcaccccaggctttacactttatgcttccggctcctatgttgtgtggaattgtgagcggataacaatttcacacaggaaacagctatgaccatgatta
cgccaagctcggaattaaccctcactaaagggaacaaaagctgggtaccgggccccccctcgagGCATAAAGGCATTAAAAGAGGAGCGAATTTTTTTTTAATAAAAATCTTAATAATCATTAAAAGATAAATAATAGTCTATATAT
ACGTATATAAATAAAAAATATTCAAAAAATAAAATAAACTATTATTTTAGCGTAAAGGATGGGGAAAGAGAAAAGAAAAAAATTGATCTATCGATTTCAATTCAATTCAATTTACTTTCTCTTCTTAGCCA
ACAAGGAACCCAAAATGTCAGTCTTTGGAGTTCTGGAAGCTCTACCCTTACCTCTTCTAGAAGCAGTCTTAGTAGAAGCAGCTGGTCTAGTCTTGGTCTTAGTCTTTCTACCGTTCAACAAACCGTAA
GAAGCGTCTTCTTCATCGGTAGAGACCATGATGATGTCGTTTTCATCGATATCAACATCATCTTGAGCGTCGTTGTTATCACCCAATTCGTTTTCAGCATCAGAAATAACAGTGGAATCGGAGAAAGC
TGGAATCTTCTTCTTGGTAGCGGTTCTGACTCTCTTTGGCTTAGAAGTTGGCTTAGAGGATTCAGCTTGAGAAATGTGAGTGATTCTGGATTCGTTATCGACGTGGGATTGAGTAATGTCTGGGGAA
CCGGTTCTGACTTCTCTGTTAGAAGATCTGAAGGAGTCCAAACCGTTACCGTTGAAAGCGAAGTTAGTTTCGTCGTTTTCCTTTGGTGGAGTTGGTCTGACGGAGTTAGCTCTCTTGACTTGCTTAAT
CAAAGCCTTCATTTCTTCAGCGTCATCAGTTCTCAAGAATTCTTCGTTGGTAGACAAGATACCAACTTCGTTGGAGATTTCGTGAGAAATGAATTCCTTCAAAGCGGTCTTTTCGTCCTTATCAACGAA
CTTCTTGACAGCTTCGTTCAAACCAACTTCTGGCAACAAGGACAATTGCATCTTGTTCAACAAGTCGTTAACCAAGGTTTGGACTTCCAATTCACCACCGGATTCAGAGAACAACTTTTCAACATCTC
TATCGGAGATAGAAGTACCGTTGATACCAGACTTCTTAGATCTGGTGACTGGAGATCTCTTCTTGTAGAATTGGACAACGTTGTTACCGTTAGCAACTCTACCGACGAATCTGTTAGAGAATCTTCTT
GGGTTTTCAACTTGGTAGTCGATTGGAGATTGGGTGTTAGATGGAGCGGAGTAATCGACTCTCAATCTGATCAATGGCTTTGGCAATTCAGCGACCATGTCACCTTCACCGTCATCAGCCAACTTTT
GCTTGGTTTCTTCGTTAGCGTCTCTAATCATTTCTTCAACTTGTTCAATCAAGTACTTAGAAGTAGCGTCCTTGTCGTGTGGTCTCAAGTGTGGAACATCTTGCAAAGAATGGACTTCATCTTGAAAG
TTCTAATAGTTTCCAATGGAATTGGGGTCATCTTTGGAGCTTCACCGTACTTAATATCCAAAATGAAAACGTACTTTGGTTGAGCTTCAGCTTCACACAAGGAAGTAGCAACAGAAGAACCTGGTTGC

AAAACGTCGAAGTTCTTAATTGGGTTGTGAACCAAGTTTGGAATACATTCGTGTTCGTGACCCCAGATGACCATGTCCAAGAAGTCTGGCAAGAATTGTTCTGGCAAGAAAGCAGTGTTAGTGTGAC
CAGTGTGGTTTTGGTGGACACACATCAAGTTGAACCATTCACCTTCTCTCATGGTTGGGACTTCGAAAGTAACACCACCATCCTTGAAAGTTCTGAACAATCTTTCATCTCTAACAGCAGCCAAACCG
TACAAAGCCAACTTAGTAGAACCCTTTTGGAACAACAATGGGACAACCTTGATCTTGTCGGATTCAATGACCTTACCGAAGTGGTTAATCAAACCGGTAGCGTGCAAAATGTCCATTGGACACAACA
AGGAATCACCAGAAGCGTCGTCGTGGTTACCGGAGATACCGAAGACTGGGATAGAGATGTTGAAGTTTGGATCTTCGTAGTTGACGTTAGTGAATTCGTCGTAGTGGAAAACTTGAGATGGATCGG
ACAACAATTCCAATTCACATGGCTTGTCACCCATACAACACAATCTCAAGGTCTTCAAGACTTGGTACAAGGACTTCTTGGATGGCTTGTTAACGTGGAACAAATCACCAGATTGAACGACCATATCA
ACGTTGTTGTTCTTAGCCAACATCATAACTTCGTGGAAAGTCTTCCAAGAGTCATCACCGGTAATTGGGTCGTTTTCGTTGTAACCGACGTGGTTGTCGGTAGTGATCAAGATTCTAATGGTATCTGG
GTCTGGGTAATCCATTGTTTTATAGGCGCGCCTTATATTGAATTTTCAAAAATTCTTACTTTTTTTTTGGATGGACGCAAAGAAGTTTAATAATCATATTACATGGCATTACCACCATATACATATCCATA
TACATATCCATATCTAATCTTACTTATATGTTGTGGAAATGTAAAGAGCCCCATTATCTTAGCCTAAAAAAACCTTCTCTTTGGAACTTTCAGTAATACGCTTAACTGCTCATTGCTATATTGAAGTACG
GATTAGAAGCCGCCGAGCGGGTGACAGCCCTCCGAAGGAAGACTCTCCTCCGTGCGTCCTCGTCTTCACCGGTCGCGTTCCTGAAACGCAGATGTGCCTCGCGCCGCACTGCTCCGAACAATAA
AGATTCTACAATACTAGCTTTTATGGTTATGAAGAGGAAAAATTGGCAGTAACCTGGCCCCACAAACCTTCAAATGAACGAATCAAATTAACAACCATAGGATGATAATGCGATTAGTTTTTTAGCCTT
ATTTCTGGGGTAATTAATCAGCGAAGCGATGATTTTTGATCTATTAACAGATATATAAATGCAAAAACTGCATAACCACTTTAACTAATACTTTCAACATTTTCGGTTTGTATTACTTCTTATTCAAATGT
AATAAAAGTATCAACAAAAAATTGTTAATATACCTCTATACTTTAACGTCAAGGAGAAAAAACTATACACCGGTGTATAAAACAATGTGGGTCGTTAGATACCAAAACACCTTGGAAGACGGTTCCATT
TCTTTCATTTCTTGTTGTTTGCAAGCTTTCAAGACCTACTCCATCGGTAGATCTTCTAAGAACCCATTGATTATCAAGAACGATAAGTCTATTTCTAGACAACACATTACCTTCAAGTGGGAAATCAACA
ACTCTTCTGACTTGAAGCACTCTTCTTTGTGTTTGGTTAACAAGGGTAAGTTGACTTCCTTGAACAAGAAGTTCATGAAGGTCGGTGAAACTTTCACCATCAACGCTTCTGATGTCTTGAAGTCTACC
ATTATTGAATTGGGTACCACTCCAATCAGAATTGAATTCGAATGGATTAACGAAGTTTGGAACATCCCACCACACTTGACTCAATTCAGAACCATGTTGTCCGAATACGGTATTTCTACCGAAATCTCT
ATTAACGATATCCCAGCTAACTTGATGATTTCTGATTACCCAAAGTCTGAAGACAACTCTATTAGAGAATTGTACGCTTTGGTCTCTACTATCCCAATGAAGAAGTCTAGATTCTTGATGGAATTGTGT
AACACTTTGTTGCCAACTTCCAAGACTAACTTGAAGTTCGACGAAATGTGGAACGATATGATTTCCAACCCAGAATACAACGTTTTCGATTTCGATCCAAACATTTTGTTGTCTAAGTTCATGAGATTG
AACAACATTAGAGTCTTGACCACTATCAAGTCTGAACCAAGATTGTCTTCTTTGTTGAGAACCTTCAACATTAACTTGTTCGCTTTCGACAACATCGACTCTTTGTACAAGTACGTTGACTCTTTGGAA
GCTTCTACTGAATACTTGATCTTGACCACTACTGATAAGAAGGAAAACGGTAAGATTTTGTGTACTATCAAGACTATGTTGACCTCCATTATCGATGGTACTTTGTCTGCTGTTATTAACATGAAGGGT
GCTTCTTCCAGAACTTTGGACAACGGTAAGTTCGACCAAATTTCCGAAGGTATGTCTACTATTTTGAAGACCTCTAGAGCTCCAGAAGTTGAAGCTTCTCCAGTTGTCTCTAAGAAGAGAAAGTTGAA
CAGAAGAAGAGTTTTGCCATTGGACTCTTTGGACTTCTTCGCTGGTGGTTTGTCCACTAAGACTTTGTCTGAAAACAGATCTTTGACTGACGCTAAGAGATTGAACTGTGGTGCTGAATCTAAGACC
GTCATCTCCTCCCCAAACATCGCTGAAGCTGATGAAAAGCACGCTCCATTCTTGCAAAACGCTTTGAAGCCAACCGAAGACATCGGTAAGAAGTCCGGTCACTCTTCTCCAGGTGCTATCATTGTTT
CTTCTCCAAACTTGGGTACCGTCAACACCTCCGAAGATTCTTTGGATAAGTCTTTGCAATCCCACAAGTTGCCACAACCATCTTTGCCAGAAGTTGCTGGTATTGGTTCTCAAACCATTTCTTCCAAC
TCTGCTGACTACGAAACTGCTGCTGTCAACTCCATGGACGACGCTGAAGTCACTAAGAACTTCAGAGTCAACCACCACCAAAACATCGAACAACCATCTAAGAACATCAGAAAGTTGTCCAACTACT
CTAGAGAAATTTCTTCTCCATTGCAAGAAAACTGTAAGTCCCCAGTTAAGGAATTGTCCATCAAGGAAAAGTCTGGTACCCCACACGCTTTCGTTGAAGCTATTCAAGAAACCAAGAACAGAGAAGTT
AAGAGAGTCAAGTCTACTATTGTCGAATTGAAGGACGAAGAATTGTCCGAAGAAGCTATCAACCAATTGAAGAACTTGGCTATCGTTGAACCATCTAACAACTTGTTGAGAAAGTCCTTCGACTCCG
AAGGTAACAAGACTTCTAGAACTACTGAAAAGTGGGAAAACTCCTTGATGGAACCAGAATGGCACAAGAGAAAGAACTTCAAGACCTTCGTTAAGGTCAGACCAAAGTCCAAGGCTCACAAGGAAG
AAGGTAAGAACAACACCCAATCTTCTGACTTCATTAGAAACGCTGCTTTCTTGATCACCAGAAACTACGTCCCATTGAAGAAGTACTCCAAGAAGGATACCACCACTAAGTGGGGTACCGAAGAAAA
CGAAGACATGTTCGCTTTGACCGAAATGGAAAGATTCGGTTCCAACACCTTCATGTCCGATAACATTAACTCCAACACCATCCAAAAGAGATCCCAAGCTTTGAACAGATTCACTAACGAAGATTCCT
CTAACGAAGGTGAAGAAGACTCTTTCTCCTTCTCCAGATGTTCTGGTACCGCTGCTTCTGTTCAACCATTGAAGAACAAGATCTTCATTACTGACGAAGATGATTTGGGTGACATTGACGACAAGTCC
GATAGATTGAACCACAGAGAAAACAACAGAAACTTGTTCGTCGTCAAGGAAATGAACTTGAGACCAAACTTGTCTGAAGAATGTTCCAAGCAATCCAGACACTCCAGATCCGCTACCTCTAGATCCA
GAGGTTCTTTCGGTGCTTCTAACAACGGTGACGGTGACGACGACGACGATGACGGTCCAAAGTTCACCTTCAAGAGAAGAAAGGGTGAAAACTTGTACTTCCAAGGTGAAGACTACAAGGACCAC
GACGGTGATTACAAGGACCACGACATTGACTACAAGGACGACGACGACAAGTAAATTGAATTGAATTGAAATCGATAGATCAATTTTTTTCTTTTCTCTTTCCCCATCCTTTACGCTAAAATAATAGTT
TATTTTATTTTTTGAATATTTTTTATTTATATACGTATATATAGACTATTATTTATCTTTTAATGATTATTAAGATTTTTATTAAAAAAAAATTCGCTCCTCTTTTAATGCCTTTATGCGCGGCCGCcaccgcggt
ggagctccaattcgccctatagtgagtcgtattacaattcactggccgtcgttttacaacgtcgtgactgggaaaaccctggcgttacccaacttaatcgccttgcagcacatccccctttcgccagctggcgtaatagcgaagaggcccgcaccgatcgcccttcccaacagttgcgcagcct
gaatggcgaatggcgcgacgcgccctgtagcggcgcattaagcgcggcgggtgtggtggttacgcgcagcgtgaccgctacacttgccagcgccctagcgcccgctcctttcgctttcttcccttcctttctcgccacgttcgccggctttccccgtcaagctctaaatcgggggctccctttagg
gttccgatttagtgctttacggcacctcgaccccaaaaaacttgattagggtgatggttcacgtagtgggccatcgccctgatagacggtttttcgccctttgacgttggagtccacgttctttaatagtggactcttgttccaaactggaacaacactcaaccctatctcggtctattcttttgatttataag
ggattttgccgatttcggcctattggttaaaaaatgagctgatttaacaaaaatttaacgcgaattttaacaaaatattaacgtttacaatttcctgatgcggtattttctccttacgcatctgtgcggtatttcacaccgcagggtaataactgatataattaaattgaagctctaatttgtgagtttagtatac
atgcatttacttataatacagtttttagttttgctggccgcatcttctcaaatatgcttcccagcctgcttttctgtaacgttcaccctctaccttagcatcccttccctttgcaaatagtcctcttccaacaataataatgtcagatcctgtagagaccacatcatccacggttctatactgttgacccaatgcgt
ctcccttgtcatctaaacccacaccgggtgtcataatcaaccaatcgtaaccttcatctcttccacccatgtctctttgagcaataaagccgataacaaaatctttgtcgctcttcgcaatgtcaacagtacccttagtatattctccagtagatagggagcccttgcatgacaattctgctaacatcaa
aaggcctctaggttcctttgttacttcttctgccgcctgcttcaaaccgctaacaatacctgggcccaccacaccgtgtgcattcgtaatgtctgcccattctgctattctgtatacacccgcagagtactgcaatttgactgtattaccaatgtcagcaaattttctgtcttcgaagagtaaaaaattgtact
tggcggataatgcctttagcggcttaactgtgccctccatggaaaaatcagtcaagatatccacatgtgtttttagtaaacaaattttgggacctaatgcttcaactaactccagtaattccttggtggtacgaacatccaatgaagcacacaagtttgtttgcttttcgtgcatgatattaaatagcttgg
cagcaacaggactaggatgagtagcagcacgttccttatatgtagctttcgacatgatttatcttcgtttcctgcaggtttttgttctgtgcagttgggttaagaatactgggcaatttcatgtttcttcaacactacatatgcgtatatataccaatctaagtctgtgctccttccttcgttcttccttctgttcgga
gattaccgaatcaaaaaaatttcaaagaaaccgaaatcaaaaaaaagaataaaaaaaaaaatgatgaattgaattgaaaagcgtggtgcactctcagtacaatctgctctgatgccgcatagttaagccagccccgacacccgccaacacccgctgacgcgccctgacgggcttgtctgc
tcccggcatccgcttacagacaagctgtgaccgtctccgggagctgcatgtgtcagaggttttcaccgtcatcaccgaaacgcgcga

3. pBC52 (Plasmid pRS303 containing Elg1 gene codon optimised for expression in *S. cerevisiae* under the control of the GAL1,10 promoter. (Gal1,10-Elg1-TEV-3xFlag, Gal4 / pRS303))

gacgaaagggcctcgtgatacgcctatttttataggttaatgtcatgataataatggtttcttagacgtcaggtggcacttttcggggaaatgtgcgcggaacccctatttgtttatttttctaaatacattcaaatatgtatccgctcatgagacaataaccctgataaatgcttcaataatattgaaaaaggaagagt
atgagtattcaacatttccgtgtcgcccttattcccttttttgcggcattttgccttcctgtttttgctcacccagaaacgctggtgaaagtaaaagatgctgaagatcagttgggtgcacgagtgggttacatcgaactggatctcaacagcggtaagatccttgagagttttcgccccgaagaacgtttt
ccaatgatgagcacttttaaagttctgctatgtggcgcggtattatcccgtattgacgccgggcaagagcaactcggtcgccgcatacactattctcagaatgacttggttgagtactcaccagtcacagaaaagcatcttacggatggcatgacagtaagagaattatgcagtgctgccataac
catgagtgataacactgcggccaacttacttctgacaacgatcggaggaccgaaggagctaaccgcttttttcacaacatgggggatcatgtaactcgccttgatcgttgggaaccggagctgaatgaagccataccaaacgacgagcgtgacaccacgatgcctgtagcaatggcaac
aacgttgcgcaaactattaactggcgaactacttactctagcttcccggcaacaattaatagactggatggaggcggataaagttgcaggaccacttctgcgctcggcccttccggctggctggtttattgctgataaatctggagccggtgagcgtgggtctcgcggtatcattgcagcactggg
gccagatggtaagccctcccgtatcgtagttatctacacgacgggcagtcaggcaactatggatgaacgaaatagacagatcgctgagataggtgcctcactgattaagcattggtaactgtcagaccaagtttactcatatatactttagattgatttaaaacttcatttttaatttaaaaggatcta
ggtgaagatcctttttgataatctcatgaccaaaatcccttaacgtgagttttcgttccactgagcgtcagaccccgtagaaaagatcaaaggatcttcttgagatcctttttttctgcgcgtaatctgctgcttgcaaacaaaaaaaccaccgctaccagcggtggtttgtttgccggatcaagagcta
ccaactctttttccgaaggtaactggcttcagcagagcgcagataccaaatactgtccttctagtgtagccgtagttaggccaccacttcaagaactctgtagcaccgcctacatacctcgctctgctaatcctgttaccagtggctgctgccagtggcgataagtcgtgtcttaccgggttggactc
aagacgatagttaccggataaggcgcagcggtcgggctgaacggggggttcgtgcacacagcccagcttggagcgaacgacctacaccgaactgagatacctacagcgtgagcattgagaaagcgccacgcttcccgaagggagaaaggcggacaggtatccggtaagcggcag
ggtcggaacaggagagcgcacgagggagcttccagggggaaacgcctggtatctttatagtcctgtcgggtttcgccacctctgacttgagcgtcgatttttgtgatgctcgtcaggggggcggagcctatggaaaaacgccagcaacgcggcctttttacggttcctggccttttgctggcctttt
gctcacatgttctttcctgcgttatcccctgattctgtggataaccgtattaccgcctttgagtgagctgataccgctcgccgcagccgaacgaccgagcgcagcgagtcagtgagcgaggaagcggaagagcgcccaatacgcaaaccgcctctccccgcgcgttggccgattcattaatgc
agctggcacgacaggtttcccgactggaaagcgggcagtgagcgcaacgcaattaatgtgagttacctcactcattaggcaccccaggctttacactttatgcttccggctcctatgttgtgtggaattgtgagcggataacaatttcacacaggaaacagctatgaccatgattacgccaagct
cggaattaaccctcactaaagggaacaaaagctgggtaccgggccccccctcgaggtcgacggtatcgataagcttgatatcgaattcctgcagcccgggGCTTTACTCTTTTTTTGGGTTTGGTGGGGTATCTTCATCATCGAATAGATAGTTATAT
ACATCATCCATTGTAGTGGTATTAAACATCCCTGTAGTGATTCCAAACGCGTTATACGCAGTTTGGTCCGTCCAACCAGGTGACAGTGGTTTTGAATTATTACCATCATCAATTTTACTAGCCGTGATT
TCATTATTCATGAAGTTATCATGAACGTTAGAGGAGGCAATTGGTTGTGAAAGCGCTTGAGAATTTGTTTGAGTTGTTATGAGGTTCGGACCGTTGCTACTGTTAGTGAAAGTGAAGGACAATGAGCT
ATCAGCAATATTCCCACTTTGATTAAAATTGGCGCCACCAAACAAAGCAGACGGGGTCAGTGGCACTAATGATTGCAGCTGTTGCTGTTGCCCTAGAAAAGGCGTGACTGAGCGATGCGAAGGTGT
GCTTCTTGGTATTGTCACTGGAGAGTTACGAGAGGGTGGACGGTTAGATAACAGCTTGACTAGATCACTGAAACTTGCTCCTGATTTCAATGGCACAGGTGAAGGCCCTACTGAGCCAGGAGAAAC
ATATTTAACACTGATATTGTTGACATTTTCCTCCGGAAGAGTAGGGTATTGGGCGATAGTTGCAGAACCGACAATATTTTTAATGGCGCTACCATTACTATTGTTATAACTGATATGCGGTAATGGGAT
TGCACACTGTGATAACAGAAACGGCGCACATACCTCTTCCAGTACTTGAATGTATTTTTCACAAGTCTGGATTTTAAAAGTGGCCAGTTTTTTTAATAGCATCAGAACAGTGTTAATTTGTTGTAATAA
TTGTGCGGTCTCGTTATTCTCAGCATTCGATTTTGAGTTTGAGAGTAGAGTCTTTATGGGTACTAGGACTGCATTGAACAAGTAATAAGAACAATTCCAGGCAAAATATGGGGTGACATTATGATTGT
CCATATAGCTACTTACAGACATAACAGTTCTTTGTGCTGCATCGCTTAACATGATGGAGCATCGTTTAACTTCATAACTTTGATGATCATTTTGATCCTGTTCTAGTTGTGACTTTTTCTGGGTAAAATT
AGTGAAAAAATCTCTTAATACATAAATGATAAGAGACAACTGTTTCCACTTCAGTTCGAATCTTGTAAAGGATAGCCAAGGGTGTTCCTTCAACAAATTGGTTAGAGCGGTGGTGGAAATATCCATTT
GTAAAAACTTTGGTGCCTGTCTCGAAACCTCCTCAATCTCATTACAAATCATCAAGCATTTTTTTGCACATATAGGACTTTTTTCTGCAGTTACTGTTTTGTCTAGTTCATAGATTTTTGTGAAAACTTGT
AAGAGCCTTGCTGTTTCAATGATGCCATGATATATGGTGGGACCTGTTGTGGTACGCTGCACATCGTCGACAGAAGAAGGGAAGGAGATTGTATTCTGAGAAAGCTGGATGGATCGACCATAAAGC
AGGGACAATTGGATCTCCCAAGAGTAGACAGACCACCAAATTCGGCGTCTTTGTTCCAGAATGCTGCTATCACTGAAGGACGAGGGGAGGTCCCTATTCAAGCCCAATGATATGGCCATTCTTATG
GAAAAGCTGTGAAAATTATAGCTAGTATTTGTTTTCTGCCTCCACTGTGTATATCGCGACAGAAGATGTAGGGCTGTCACCAAAATTATGGAACCTGACTCGAAGACCTTGCTCGTCAAATGAGATTT
AGCATTTTGATAGTAAAAAACATCTATATCAGTAGATTCCCCCTCTATACACCAGGCTCCAATGGCTAATATGCAGTTAAAAAGGATTTGCCATTGATCCTTCGACGCGATTTCAATCTGGTTATTATA
CAACATCATTAGCGTCGGTGAGTGCACGATAGGGCAGTAGGGGTGAAAATTATTGAGATAACTTTGAAGTAAACGGGATGTTGTGGATCTAGAAGCCAACGTGTATCTATCCGTAATCATGGTCGG
GAGCCTGTTAACGTTAGAGTTCGTGTAATTTTCCGGTTTAAAGCCAATAGATCGAAGAATACATAAGAGAGAACCGTCGCCAAAGAACCCATTATTGTTGGGGTCCGTTTTCAGGAAGGGCAAGCCA
TCCGACATGTCATCCTCTTCAGACCAATCAAATCCATGAAGAGCATCCCTGGGCATAAAATCCAACGGAATTGTGGAGTTATCATGATGAGCTGCCGAGTCAATCGATACAGTCAACTGTCTTTGAC
CTTTGTTACTACTCTCTTCCGATGATGATGTCGCACTTATTCTATGCTGTCTCAATGTTAGAGGCATATCAGTCTCCACTGAAGCCAATCTATCTGTGACGGCATCTTTATTCACATTATCTTGTACAA
ATAATCCTGTTAACAATGCTTTTATATCCTGTAAAGAATCCATTTTCAAAATCATGTCAAGGTCTTCTCGAGGAAAAATCAGTAGAAATAGCTGTTCCAGTCTTTCTAGCCTTGATTCCACTTCTGTCAG
ATGTGCCCTAGTCAGCGGAGACCTTTTGGTTTTGGGAGAGTAGCGACACTCCCAGTTGTTCTTCAGACACTTGGCGCACTTCGGTTTTTCTTTGGAGCACTTGAGCTTTTTAAGTCGGCAAATATCG
CATGCTTGTTCGATAGAAGACAGTAGCTTCATGGCGCGCCTTATATTGAATTTTCAAAAATTCTTACTTTTTTTTTGGATGGACGCAAAGAAGTTTAATAATCATATTACATGGCATTACCACCATATAC
ATATCCATATCTAATCTTACTTATATGTTGTGGAAATGTAAAGAGCCCCATTATCTTAGCCTAAAAAAACCTTCTCTTTGGAACTTTCAGTAATACGCTTAACTGCTCATTGCTATATTGAAGTACGGAT
TAGAAGCCGCCGAGCGGGCGACAGCCCTCCGACGGAAGACTCTCCTCCGTGCGTCCTCGTCTTCACCGGTCGCGTTCCTGAAACGCAGATGTGCCTCGCGCCGCACTGCTCCGAACAATAAAGA
TTCTACAATACTAGCTTTTATGGTTATGAAGAGGAAAAATTGGCAGTAACCTGGCCCCACAAACCTTCAAATTAACGAATCAAATTAACAACCATAGGATGATAATGCGATTAGTTTTTTAGCCTTATTT
CTGGGGTAATTAATCAGCGAAGCGATGATTTTTGATCTATTAACAGATATATAAATGGAAAAGCTGCATAACCACTTTAACTAATACTTTCAACATTTTCAGTTTGTATTACTTCTTATTCAAATGTCATA
AAAGTATCAACAAAAAATTGTTAATATACCTCTATACTTTAACGTCAAGGAGAAAAAACTATACACCGGTGACGTTAATTAACACCGGTGTATAAAACAATGAAGAGACACGTCTCTTTGTCTGATATT
TTGACTGGTAACAAGAGAAAGGTTAGAAGACAAGATGCTTTGCAAATTACCATTGACGACGAAAACGACACCGAATCTGGTACTTTCGATGCTAGAACCGCTAAGCACGACGATTCCTCCGTCATCT
TCTTGAACCACTCTGTTGTCAAGCCAATTGAAGCTGTCTCTACCAACCACAAGTCCGCTAAGGAATTCTTGATGACTAAGAGAACTAAGGAAAAGTGTGATGACGATGATGACGATTTGATTGTCATT
TCTGACAAGTCCCCAAAGTCTGAAACCAACTGTTCCAAGATCGCTTTGTCCCAAGAACACGAAGACGACATCTCTATCATCTCCACTTCCAGAATTAAGTCTTCCTTGTTGAACGAAAGAGCTTCTAA
GATCAAGAACTTCTTGAAGCACGAAACTACTGACACTTTCAAGAGATTGAACTCTATTTCTAAGTTGAACGAAATCGAACCACCATTGCCATTGCACCAATCCATCTTCCCAGTTGGTGACAAGGAAT
TGTCTGATAGATCCGTCGATATTCCATTGCCATTCAGAACCATTCCACCATTGAACCACAACTTCTTGCCATCCGACTACGAATCTTTGAAGGATAAGAACTCTGCTTCCTGTATCCCAGTCAGATAC
CAAGCTCCAGTTTTGTTGGGTACTAACATTAAGAGAAACACTACCTTGACCTGGCCACAATTGTTCAAGCCAGTCACTTTGAAGCAAGTTTTGATCGAACCAAAGTTGAAGTTGAGAATTAAGAACTG
GATCGAAACCTCCTTCCACACTTTGGAAAAGCCAACTTTGAGAAACAGATTGTTGAACAGAATTAACCCAAACAAGCAACAAGGTTCCGGTGACGAATTGGCTAACTTCATCGTTCCAGATTTGGAA
GAAGACGAAAACTTGAGACCAGACTTCTACAGAAACGGTGAAGCTAACTCCTCTTTGTCTGAATTCGTTCCATTGATGATTTTGCACGGTAACTCTATTGGTAAGAAGACTTTGATCCAAACCATCAT
GAGAGAAATCGCTGGTGATGATAACTCCTACCAAATCTACGAAGTCAACTCTAACATGAACAGATCCAAGAAGGACTTGTTGGATATCTTGTTGGATTTCACTACCACTCACTACGTTAAGGACTCTT
CTAAGAGAAAGTCCGATTACGGTTTGGTCTTGTTCAACGATGTTGATGTTTTGTTCAAGGAACACGACAGAGGTTACTGGGCTATGATTTCTAAGTTGTGTGAATTCTCTAGAAGACCATTGGTTTTG
ACTTGTAAGGACTTGTCTTTGGTTCCATCTGAATTGATCGCTTTGGCTTCCGAACAAAACTCTTTGTTCCACACCAAGAAGATCTCCACTTCTACTGTCTACGCTTTCTTGACCAAGTACTTGAAGTCC
TTGGAAATTGAAGTTTGTGACGATTGGTTGAGAGACGTTGTCAAGCAAAACAACGCTGACATTAGAAAGTGTTTGATGCACTTGCAATTCTGGTGTGTCGACACTGAAGCTGATTTGATCTCCTCTAA
GAACAGATTGCCAGTCTTGACCTCTACTTTGGGTTCTTCTGTTAAGGACATTTCTCAATTGACTGATTTGTTGTCTATTAACGACGTTATTGGTCAAGCTACCTTGAACAGATCTATGGTCAGACAAGA
AATCGATTCTACCACCATGACCCCAGAAAAGGTTAACACCTTCCAAGACCAAAACTTGGATGACGAAATGAAGTTGAAGTTCGATTACGTTATCGACTACAAGTTGCACTTGAACGATCCAAACAGA
CAACCATTGTTGCCATTCGAATTGAACATCTACCAACACATTCAAGAACAATTGGAAGCTAGATACTCTTACGTTAGAGAAGCTAACCACAGATTGGACAACGAATACTTGGTTAACAGATTCAAGAA
GATGACTGAATCCACCTTGAACTTCTTGGCTTCTAGAATTCCAAAGTACGACCACTTGCAATCTGCTAGAAGAACCAGAAACTCTAAGAAGATCTCCGATATCTTGAACCAATTCAAGGGTATCTACA
ACGACGAAACTTTGAACGAAAACGCTGAAATCGACTTGTTGTCTGCTACCACTCAACAAATCAAGGCTGAAATTAACCCATTCGTCTTCGAAATTGCTAAGTCTGATGCTAACGTTAAGAACGAAAAC
AAGCAAATCTTCGAATTGCACTCTGAAAACGTCTCTGAAAGAAGATACAAGGATTTGGTTTACCAATTGTCTCAAGAAGGTGTTTTGAAGAACGTCTGGTTCAACGCTGATCCATCTATTGTCGTTAG
AAAGTGGGAACACTTGCACTCTGGTTTCTCTAAGAACAAGGAAAACTTGTACTTCCAAGGTGAAGATTACAAGGATCACGACGGTGATTACAAGGACCACGACATTGACTACAAGGACGACGACGA
CAAGTAAATTGAATTGAATTGAAATCGATAGATCAATTTTTTTCTTTTCTCTTTCCCCATCCTTTACGCTAAAATAATAGTTTATTTTATTTTTTGAATATTTTTTATTTATATACGTATATATAGACTATTAT
TTATCTTTTAATGATTATTAAGATTTTTATTAAAAAAAAATTCGCTCCTCTTTTAATGCCTTTATGCGCGGCCGCcaccgcggtggagctccaattcgccctatagtgagtcgtattacaattcactggccgtcgttttacaacgtcgtgactggg
aaaaccctggcgttacccaacttaatcgccttgcagcacatccccctttcgccagctggcgtaatagcgaagaggcccgcaccgatcgcccttcccaacagttgcgcagcctgaatggcgaatggcgcgacgcgccctgtagcggcgcattaagcgcggcgggtgtggtggttacgcgc
agcgtgaccgctacacttgccagcgccctagcgcccgctcctttcgctttcttcccttcctttctcgccacgttcgccggctttccccgtcaagctctaaatcgggggctccctttagggttccgatttagtgctttacggcacctcgaccccaaaaaacttgattagggtgatggttcacgtagtgggcc
atcgccctgatagacggtttttcgccctttgacgttggagtccacgttctttaatagtggactcttgttccaaactggaacaacactcaaccctatctcggtctattcttttgatttataagggattttgccgatttcggcctattggttaaaaaatgagctgatttaacaaaaatttaacgcgaattttaacaa
aatattaacgtttacaatttcctgatgcggtattttctccttacgcatctgtgcggtatttcacaccgcatatgatccgtcgagttcaagagaaaaaaaaagaaaaagcaaaaagaaaaaaggaaagcgcgcctcgttcagaatgacacgtatagaatgatgcattaccttgtcatcttcagtatc
atactgttcgtatacatacttactgacattcataggtatacatatatacacatgtatatatatcgtatgctgcagctttaaataatcggtgtcactacataagaacacctttggtggagggaacatcgttggtaccattgggcgaggtggcttctcttatggcaaccgcaagagccttgaacgcactctc
actacggtgatgatcattcttgcctcgcagacaatcaacgtggagggtaattctgctagcctctgcaaagctttcaagaaaatgcgggatcatctcgcaagagagatctcctactttctccctttgcaaaccaagttcgacaactgcgtacggcctgttcgaaagatctaccaccgctctggaaag
tgcctcatccaaaggcgcaaatcctgatccaaacctttttactccacgcgccagtagggcctctttaaaagcttgaccgagagcaatcccgcagtcttcagtggtgtgatggtcgtctatgtgtaagtcaccaatgcactcaacgattagcgaccagccggaatgcttggccagagcatgtatca
tatggtccagaaaccctatacctgtgtggacgttaatcacttgcgattgtgtggcctgttctgctactgcttctgcctctttttctgggaagatcgagtgctctatcgctaggggaccaccctttaaagagatcgcaatctgaatcttggtttcatttgtaatacgctttactagggctttctgctctgtcatctttg
ccttcgtttatcttgcctgctcatttttagtatattcttcgaagaaatcacattactttatataatgtataattcattatgtgataatgccaatcgctaagaaaaaaaaagagtcatccgctaggtggaaaaaaaaaaatgaaaatcattaccgaggcataaaaaaatatagagtgtactagaggagg
ccaagagtaatagaaaaagaaaattgcgggaaaggactgtgttatgacttccctgactaatgccgtgttcaaacgatacctggcagtgactcctagcgctcaccaagctcttaaaacggaattatggtgcactctcagtacaatctgctctgatgccgcatagttaagccagccccgacaccc
gccaacacccgctgacgcgccctgacgggcttgtctgctcccggcatccgcttacagacaagctgtgaccgtctccgggagctgcatgtgtcagaggttttcaccgtcatcaccgaaacgcgcga

4. pBC65 (Plasmid pRS306 containing PCNA gene from *S. cerevisiae* under the GAL1,10 promoter. (Gal1,10-PCNA / pRS306))

GTGGTGGTTACGCGCAGCGTGACCGCTACACTTGCCAGCGCCCTAGCGCCCGCTCCTTTCGCTTTCTTCCCTTCCTTTCTCGCCACGTTCGCCGGCTTTCCCCGTCAAGCTCTAAATCGGGGGCT
CCCTTTAGGGTTCCGATTTAGTGCTTTACGGCACCTCGACCCCAAAAAACTTGATTAGGGTGATGGTTCACGTAGTGGGCCATCGCCCTGATAGACGGTTTTTCGCCCTTTGACGTTGGAGTCCAC
GTTCTTTAATAGTGGACTCTTGTTCCAAACTGGAACAACACTCAACCCTATCTCGGTCTATTCTTTTGATTTATAAGGGATTTTGCCGATTTCGGCCTATTGGTTAAAAAATGAGCTGATTTAACAAAAA
TTTAACGCGAATTTTAACAAAATATTAACGCTTACAATTTCCTGATGCGGTATTTTCTCCTTACGCATCTGTGCGGTATTTCACACCGCATAGGGTAATAACTGATATAATTAAATTGAAGCTCTAATTT
GTGAGTTTAGTATACATGCATTTACTTATAATACAGTTTTTTAGTTTTGCTGGCCGCATCTTCTCAAATATGCTTCCCAGCCTGCTTTTCTGTAACGTTCACCCTCTACCTTAGCATCCCTTCCCTTTGC
AAATAGTCCTCTTCCAACAATAATAATGTCAGATCCTGTAGAGACCACATCATCCACGGTTCTATACTGTTGACCCAATGCGTCTCCCTTGTCATCTAAACCCACACCGGGTGTCATAATCAACCAAT
CGTAACCTTCATCTCTTCCACCCATGTCTCTTTGAGCAATAAAGCCGATAACAAAATCTTTGTCGCTCTTCGCAATGTCAACAGTACCCTTAGTATATTCTCCAGTAGATAGGGAGCCCTTGCATGAC
AATTCTGCTAACATCAAAAGGCCTCTAGGTTCCTTTGTTACTTCTTCTGCCGCCTGCTTCAAACCGCTAACAATACCTGGGCCCACCACACCGTGTGCATTCGTAATGTCTGCCCATTCTGCTATTCT
GTATACACCCGCAGAGTACTGCAATTTGACTGTATTACCAATGTCAGCAAATTTTCTGTCTTCGAAGAGTAAAAAATTGTACTTGGCGGATAATGCCTTTAGCGGCTTAACTGTGCCCTCCATGGAAA
AATCAGTCAAGATATCCACATGTGTTTTTAGTAAACAAATTTTGGGACCTAATGCTTCAACTAACTCCAGTAATTCCTTGGTGGTACGAACATCCAATGAAGCACACAAGTTTGTTTGCTTTTCGTGCA
TGATATTAAATAGCTTGGCAGCAACAGGACTAGGATGAGTAGCAGCACGTTCCTTATATGTAGCTTTCGACATGATTTATCTTCGTTTCCTGCAGGTTTTTGTTCTGTGCAGTTGGGTTAAGAATACT
GGGCAATTTCATGTTTCTTCAACACTACATATGCGTATATATACCAATCTAAGTCTGTGCTCCTTCCTTCGTTCTTCCTTCTGTTCGGAGATTACCGAATCAAAAAAATTTCAAGGAAACCGAAATCAA
AAAAAAGAATAAAAAAAAAATGATGAATTGAAAAGGTGGTATGGTGCACTCTCAGTACAATCTGCTCTGATGCCGCATAGTTAAGCCAGCCCCGACACCCGCCAACACCCGCTGACGCGCCCTGAC
GGGCTTGTCTGCTCCCGGCATCCGCTTACAGACAAGCTGTGACCGTCTCCGGGAGCTGCATGTGTCAGAGGTTTTCACCGTCATCACCGAAACGCGCGAGACGAAAGGGCCTCGTGATACGCCT
ATTTTTATAGGTTAATGTCATGATAATAATGGTTTCTTAGACGTCAGGTGGCACTTTTCGGGGAAATGTGCGCGGAACCCCTATTTGTTTATTTTTCTAAATACATTCAAATATGTATCCGCTCATGAG
ACAATAACCCTGATAAATGCTTCAATAATATTGAAAAAGGAAGAGTATGAGTATTCAACATTTCCGTGTCGCCCTTATTCCCTTTTTTGCGGCATTTTGCCTTCCTGTTTTTGCTCACCCAGAAACGCT
GGTGAAAGTAAAAGATGCTGAAGATCAGTTGGGTGCACGAGTGGGTTACATCGAACTGGATCTCAACAGCGGTAAGATCCTTGAGAGTTTTCGCCCCGAAGAACGTTTTCCAATGATGAGCACTTT
TAAAGTTCTGCTATGTGGCGCGGTATTATCCCGTATTGACGCCGGGCAAGAGCAACTCGGTCGCCGCATACACTATTCTCAGAATGACTTGGTTGAGTACTCACCAGTCACAGAAAAGCATCTTAC
GGATGGCATGACAGTAAGAGAATTATGCAGTGCTGCCATAACCATGAGTGATAACACTGCGGCCAACTTACTTCTGACAACGATCGGAGGACCGAAGGAGCTAACCGCTTTTTTGCACAACATGGG
GGATCATGTAACTCGCCTTGATCGTTGGGAACCGGAGCTGAATGAAGCCATACCAAACGACGAGCGTGACACCACGATGCCTGTAGCAATGGCAACAACGTTGCGCAAACTATTAACTGGCGAAC
TACTTACTCTAGCTTCCCGGCAACAATTAATAGACTGGATGGAGGCGGATAAAGTTGCAGGACCACTTCTGCGCTCGGCCCTTCCGGCTGGCTGGTTTATTGCTGATAAATCTGGAGCCGGTGAGC
GTGGGTCTCGCGGTATCATTGCAGCACTGGGGCCAGATGGTAAGCCCTCCCGTATCGTAGTTATCTACACGACGGGGAGTCAGGCAACTATGGATGAACGAAATAGACAGATCGCTGAGATAGGT
GCCTCACTGATTAAGCATTGGTAACTGTCAGACCAAGTTTACTCATATATACTTTAGATTGATTTAAAACTTCATTTTTAATTTAAAAGGATCTAGGTGAAGATCCTTTTTGATAATCTCATGACCAAAAT
CCCTTAACGTGAGTTTTCGTTCCACTGAGCGTCAGACCCCGTAGAAAAGATCAAAGGATCTTCTTGAGATCCTTTTTTTCTGCGCGTAATCTGCTGCTTGCAAACAAAAAAACCACCGCTACCAGCG
GTGGTTTGTTTGCCGGATCAAGAGCTACCAACTCTTTTTCCGAAGGTAACTGGCTTCAGCAGAGCGCAGATACCAAATACTGTTCTTCTAGTGTAGCCGTAGTTAGGCCACCACTTCAAGAACTCTG
TAGCACCGCCTACATACCTCGCTCTGCTAATCCTGTTACCAGTGGCTGCTGCCAGTGGCGATAAGTCGTGTCTTACCGGGTTGGACTCAAGACGATAGTTACCGGATAAGGCGCAGCGGTCGGGC
TGAACGGGGGGTTCGTGCACACAGCCCAGCTTGGAGCGAACGACCTACACCGAACTGAGATACCTACAGCGTGAGCTATGAGAAAGCGCCACGCTTCCCGAAGGGAGAAAGGCGGACAGGTATC
CGGTAAGCGGCAGGGTCGGAACAGGAGAGCGCACGAGGGAGCTTCCAGGGGGAAACGCCTGGTATCTTTATAGTCCTGTCGGGTTTCGCCACCTCTGACTTGAGCGTCGATTTTTGTGATGCTC
GTCAGGGGGGCGGAGCCTATGGAAAAACGCCAGCAACGCGGCCTTTTTACGGTTCCTGGCCTTTTGCTGGCCTTTTGCTCACATGTTCTTTCCTGCGTTATCCCCTGATTCTGTGGATAACCGTAT
TACCGCCTTTGAGTGAGCTGATACCGCTCGCCGCAGCCGAACGACCGAGCGCAGCGAGTCAGTGAGCGAGGAAGCGGAAGAGCGCCCAATACGCAAACCGCCTCTCCCCGCGCGTTGGCCGAT
TCATTAATGCAGCTGGCACGACAGGTTTCCCGACTGGAAAGCGGGCAGTGAGCGCAACGCAATTAATGTGAGTTAGCTCACTCATTAGGCACCCCAGGCTTTACACTTTATGCTTCCGGCTCGTAT
GTTGTGTGGAATTGTGAGCGGATAACAATTTCACACAGGAAACAGCTATGACCATGATTACGCCAAGCTCGAAATTAACCCTCACTAAAGGGAACAAAAGCTGGTACCGGGCCCCCCCTCGAGTTT
GCTAGCTTTGGCGCGCCTTATATTGAATTTTCAAAAATTCTTACTTTTTTTTTGGATGGACGCAAAGAAGTTTAATAATCATATTACATGGCATTACCACCATATACATATCCATATACATATCCATATCT
AATCTTACTTATATGTTGTGGAAATGTAAAGAGCCCCATTATCTTAGCCTAAAAAAACCTTCTCTTTGGAACTTTCAGTAATACGCTTAACTGCTCATTGCTATATTGAAGTACGGATTAGAAGCCGCC
GAGCGGGTGACAGCCCTCCGAAGGAAGACTCTCCTCCGTGCGTCCTCGTCTTCACCGGTCGCGTTCCTGAAACGCAGATGTGCCTCGCGCCGCACTGCTCCGAACAATAAAGATTCTACAATACT
AGCTTTTATGGTTATGAAGAGGAAAAATTGGCAGTAACCTGGCCCCACAAACCTTCAAATGAACGAATCAAATTAACAACCATAGGATGATAATGCGATTAGTTTTTTAGCCTTATTTCTGGGGTAATT
AATCAGCGAAGCGATGATTTTTGATCTATTAACAGATATATAAATGCAAAAACTGCATAACCACTTTAACTAATACTTTCAACATTTTCGGTTTGTATTACTTCTTATTCAAATGTAATAAAAGTATCAAC
AAAAAATTGTTAATATACCTCTATACTTTAACGTCAAGGAGAAAAAACTATACACCGGTGctgcaatacaccggtgATGTTAGAAGCAAAATTTGAAGAAGCATCCCTTTTCAAGAGAATAATTGATGGTTTCA
AAGATTGTGTCCAGTTGGTCAATTTCCAATGTAAAGAAGATGGTATCATTGCACAAGCTGTCGATGACTCAAGAGTTCTATTGGTCTCCTTGGAAATAGGTGTCGAAGCCTTCCAAGAATATAGATGT
GACCATCCTGTTACGTTAGGTATGGATCTAACCTCACTAAGTAAAATCCTACGTTGTGGTAACAACACCGATACATTAACACTAATTGCTGACAACACACCGGATTCCATCATCTTATTATTTGAGGAT
ACCAAGAAAGACCGTATAGCCGAATACTCTCTGAAATTGATGGATATCGATGCTGATTTCTTAAAGATTGAAGAATTACAGTACGACTCCACCCTGTCATTGCCATCTTCCGAATTCTCTAAAATTGTT
CGTGACTTGTCCCAATTGAGTGATTCTATTAATATCATGATCACCAAAGAAACAATAAAGTTTGTAGCTGACGGTGATATCGGATCAGGTTCAGTCATAATAAAACCATTCGTGGATATGGAACATCC
TGAAACAAGCATCAAACTTGAAATGGATCAACCTGTCGACTTGACGTTCGGAGCTAAATATTTATTGGACATCATTAAGGGCTCCTCCCTTTCTGATAGAGTTGGTATCAGGCTCTCCAGCGAAGCT
CCTGCTTTATTCCAATTTGATTTGAAGAGTGGGTTCCTACAGTTTTTCTTGGCTCCTAAATTTAATGACGAAGAATAAGCGGCCGCcaccgcggTAAAGCGCGGCCGCCACCGCGGTGGAGCTCCAATT
CGCCCTATAGTGAGTCGTATTACAATTCACTGGCCGTCGTTTTACAACGTCGTGACTGGGAAAACCCTGGCGTTACCCAACTTAATCGCCTTGCAGCACATCCCCCTTTCGCCAGCTGGCGTAATA
GCGAAGAGGCCCGCACCGATCGCCCTTCCCAACAGTTGCGCAGCCTGAATGGCGAATGGACGCGCCCTGTAGCGGCGCATTAAGCGCGGCGGGT

5. pBC66 (Plasmid pRS304 containing RFC1 gene from *S. cerevisiae* under the GAL1,10 promoter. (Gal1,10-3xFlag-Rfc1 / pRS304))

GACGTTGTAAAACGACGGCCAGTGAATTGTAATACGACTCACTATAGGGCGAATTGGAGCTCCACCGCGGTGGCGGCCGCGCTTTATCATGCTTTCGTTTTCCTTTTTTTGCTACCACCAGGTTTAC
TGGTGGCAGTCTTCCTCTTCGTAGGTTTGGCTTTCTGCTTGATAAGTTTATCCTTCTTCAAGTCTGTGCTACTATCTTGTGTTTCTTCATCATCTGCGGGAACTGGATTGTCATCTGCGTCAACGACAT
CTTCGAAATCGGGGGTGCTAGTACTGGTGCCAACACCACCACCACCAATAGTACTACCTGTTCTGTAAATTGCGACTGGATGTGTCATACTGTTGTATTTCCGCGTGAATCCACTTTTAACCGTAGC
TGGTATCTTTTTGATAATGGCTGTGGTCACATCAGGACCTACGAAAAACTCCATAATAGAATCCCAGTCTTCTTTGGTCAAATAGTAATCGTCCATTACCTCTATGACAGATGAAATTGCATCAGCACC
TTGCTTCAAAAATGGGTCCAATAATCTTTTTCTAAAAGTTGGTAGGTAATCCAGCCTTAGTCCAATTTTATCAGTAGATGTACCTAGTCTTGTATGATAATGTATCTCTTGCAGTAACCTATAATATTTG
GCAGATTTAGAATTTTGGCCCAACCAAGCTGTGAAATTGATTCTTCCCGCCATATGGCCGGCAACTTTTGATGCTGGATAAACAGATGAAAGAACAGCATGCAAAGGCAAAAGACTCCATAATTGCT
CACTACTACGAATCTTCTTTTCGACAATATCACCTAATGAAATACAGTTAGCCGCCTCAGCAACTGCTTCCAAATGTGACTGGCCGGGTTTCAAAACGCTTGGTCTTGTAGATAAATAGTTTTCCTGA
ATCATTAGGGGTGTGAAATCAAAGTCATCAAAATATAATGCGATCTTATCATTCAGTGTAAAGTTCCTTGAACCTATGTCTGAGTATATTTGTCCATCCAACATTTTATGGGCAATGTCAAAGGGTTTT
AGGGCAATATTCTTTTCCCATGCCTTTGAGATCTCGTTGATATTTTCATGGTTTATGGTTTTAGTAGTCGTAGATATCGTTGAAAGTAGATTAATAACCTGGCGGATATCACCTCTTGTAGTCTGTATC
AACCTATCAATGACATTTGGATCAAGTTTGAACTTTTCTCTGATCGCAATTGTCATTAATCTTGATTTGATACTGTTAGCATCAGGTCTTCTAAATTGGATATCAAGACATACTCTATCGAATGGTCTCA
TTTTTGGTAAATTACGTTCATTACAAATTAGAATCAATGGAGTTGACGTCTTACGACAAAACTGTGCCAGCTGGCCAACTCCCCCCCTATCGCCTCCGCTCATACCATCAACTTCATCCATGATAATG
ACAAAATGTTTCCCGTTCAAATTTTGAGCTTCTTCGTTGTGTTTGAAATATCCCACTACAGACATGTTATCAAGAGCGTTTTTAACACCGGCATTAAGTAAAGTTTTAGAGCGAACATCAGAAGCGTTT
TGTTCAAGAATGTCATACCCAAGCTCTTGTGCAACTAAATGAGCAGCAGTTGTCTTCCCAATACCAGGAGGACCGTATAACATTGCAGCCCTGAAAACACCAGAGCCATCTTTACCAGCATGTTTAA
AACTATTTTTCTTTGAATTTTCCCAATTAGCCAACCAGTTTTTTAATTTCATTACACTTCCTTTGTTACCACAGACCTGTTGTAGATTCGTTGGTGCGTACTTTACTGTCCACAATTTATCTTCTTCCCTA
ACCACATTGTCTCTCTCCAGATGGCCACCAGAAACTCTAGTGGCTGCAAGCTTCTTCGACCTTTCCTCTTCTTTCTTAACAAGCAATTCTGCTTCTTTGGTAGCAATATTATGCTGTTCTTCTAATTTTC
GACGTGCTTTTTCGGCAGCTTCCCCGTCACCACCTTCAGCAGGCATCCCAGCAATTAATTGCTTAAAGCCTTCTTCGTCTATGGCTTTGATTTTCAATTGCTTGATTTTTTCTAGTTTCTTTGGCCCTG
CTTCATCACCTAAGACTACTACAGAAGTTTTGCTTGAAATTGATTTAGTAACCCTTGCTCCGTATCTTTTCGCTAAAGCTTCCGATGCACCACGCTCCAAGGTTGGCAGAACACCTGTGAAGACAATT
GTTAGACCTAATAAACAATTTGGCTTTCCTTCTGGAAAACTACCTATTTCAGAAACAATTTCATCTGGATCCGCATTTGAATTTGCGCTTTTGAAATCAAATTTAGCATTCTCTTTCACATGAACATTTG
ACAGATCCAAGGATGGAATCTTATCTAGTACATCTTGAGCCGTAATATTCGAAGCCGAGTCTGAACTTTTAGAGGAGGGTTTGGTTTTTTTTGAACTTGCTGGCTTTACAGTTGGTGAAGAGGCCTTC
CTCTTAGCGGGAAGAGGTAGCTTTTTTTCTCCTTCAGGTGTCTCTGATACATCAATTACATTACTTACAGGCATCTTCTTTGGTGTTTTATTGGTTGATTCTTGGTCAGATTCAGTATCTAAGTCGATAA
CTTCTGGTTTAGACGAACCCACCTGTCTCGTAGGTCTGGATGTTGACGATCTTACGGATTTCTTATTTTTACCAAAGAAATCAGAAATATTGACCATcttgtcatcgtcatccttgtaatcgatgtcatgatctttataatcaccgt
catggtctttgtagtccattgttttataCACCGGTGTATAGTTTTTTCTCCTTGACGTTAAAGTATAGAGGTATATTAACAATTTTTTGTTGATACTTTTATTACATTTGAATAAGAAGTAATACAAACCGAAAATGTTGAA
AGTATTAGTTAAAGTGGTTATGCAGTTTTTGCATTTATATATCTGTTAATAGATCAAAAATCATCGCTTCGCTGATTAATTACCCCAGAAATAAGGCTAAAAAACTAATCGCATTATCATCCTATGGTTG
TTAATTTGATTCGTTCATTTGAAGGTTTGTGGGGCCAGGTTACTGCCAATTTTTCCTCTTCATAACCATAAAAGCTAGTATTGTAGAATCTTTATTGTTCGGAGCAGTGCGGCGCGAGGCACATCTGC
GTTTCAGGAACGCGACCGGTGAAGACGAGGACGCACGGAGGAGAGTCTTCCTTCGGAGGGCTGTCACCCGCTCGGCGGCTTCTAATCCGTACTTCAATATAGCAATGAGCAGTTAAGCGTATTAC
TGAAAGTTCCAAAGAGAAGGTTTTTTTAGGCTAAGATAATGGGGCTCTTTACATTTCCACAACATATAAGTAAGATTAGATATGGATATGTATATGGATATGTATATGGTGGTAATGCCATGTAATATG
ATTATTAAACTTCTTTGCGTCCATCCAAAAAAAAAAGTAAGAATTTTTGAAAATTCAATATAAGGCGCGCCAAAGCTAGCAAACTCGAGGGGGGGCCCGGTACCAGCTTTTGTTCCCTTTAGTGAGGGT
TAATTTCGAGCTTGGCGTAATCATGGTCATAGCTGTTTCCTGTGTGAAATTGTTATCCGCTCACAATTCCACACAACATACGAGCCGGAAGCATAAAGTGTAAAGCCTGGGGTGCCTAATGAGTGAG
CTAACTCACATTAATTGCGTTGCGCTCACTGCCCGCTTTCCAGTCGGGAAACCTGTCGTGCCAGCTGCATTAATGAATCGGCCAACGCGCGGGGAGAGGCGGTTTGCGTATTGGGCGCTCTTCCG
CTTCCTCGCTCACTGACTCGCTGCGCTCGGTCGTTCGGCTGCGGCGAGCGGTATCAGCTCACTCAAAGGCGGTAATACGGTTATCCACAGAATCAGGGGATAACGCAGGAAAGAACATGTGAGCA
AAAGGCCAGCAAAAGGCCAGGAACCGTAAAAAGGCCGCGTTGCTGGCGTTTTTCCATAGGCTCCGCCCCCCTGACGAGCATCACAAAAATCGACGCTCAAGTCAGAGGTGGCGAAACCCGACAG
GACTATAAAGATACCAGGCGTTTCCCCCTGGAAGCTCCCTCGTGCGCTCTCCTGTTCCGACCCTGCCGCTTACCGGATACCTGTCCGCCTTTCTCCCTTCGGGAAGCGTGGCGCTTTCTCATAGCT
CACGCTGTAGGTATCTCAGTTCGGTGTAGGTCGTTCGCTCCAAGCTGGGCTGTGTGCACGAACCCCCCGTTCAGCCCGACCGCTGCGCCTTATCCGGTAACTATCGTCTTGAGTCCAACCCGGTA
AGACACGACTTATCGCCACTGGCAGCAGCCACTGGTAACAGGATTAGCAGAGCGAGGTATGTAGGCGGTGCTACAGAGTTCTTGAAGTGGTGGCCTAACTACGGCTACACTAGAAGAACAGTATT
TGGTATCTGCGCTCTGCTGAAGCCAGTTACCTTCGGAAAAAGAGTTGGTAGCTCTTGATCCGGCAAACAAACCACCGCTGGTAGCGGTGGTTTTTTTGTTTGCAAGCAGCAGATTACGCGCAGAAA
AAAAGGATCTCAAGAAGATCCTTTGATCTTTTCTACGGGGTCTGACGCTCAGTGGAACGAAAACTCACGTTAAGGGATTTTGGTCATGAGATTATCAAAAAGGATCTTCACCTAGATCCTTTTAAATT
AAAAATGAAGTTTTAAATCAATCTAAAGTATATATGAGTAAACTTGGTCTGACAGTTACCAATGCTTAATCAGTGAGGCACCTATCTCAGCGATCTGTCTATTTCGTTCATCCATAGTTGCCTGACTCC
CCGTCGTGTAGATAACTACGATACGGGAGGGCTTACCATCTGGCCCCAGTGCTGCAATGATACCGCGAGACCCACGCTCACCGGCTCCAGATTTATCAGCAATAAACCAGCCAGCCGGAAGGGC
CGAGCGCAGAAGTGGTCCTGCAACTTTATCCGCCTCCATCCAGTCTATTAATTGTTGCCGGGAAGCTAGAGTAAGTAGTTCGCCAGTTAATAGTTTGCGCAACGTTGTTGCCATTGCTACAGGCATC
GTGGTGTCACGCTCGTCGTTTGGTATGGCTTCATTCAGCTCCGGTTCCCAACGATCAAGGCGAGTTACATGATCCCCCATGTTGTGCAAAAAAGCGGTTAGCTCCTTCGGTCCTCCGATCGTTGTC
AGAAGTAAGTTGGCCGCAGTGTTATCACTCATGGTTATGGCAGCACTGCATAATTCTCTTACTGTCATGCCATCCGTAAGATGCTTTTCTGTGACTGGTGAGTACTCAACCAAGTCATTCTGAGAATA
GTGTATGCGGCGACCGAGTTGCTCTTGCCCGGCGTCAATACGGGATAATACCGCGCCACATAGCAGAACTTTAAAAGTGCTCATCATTGGAAAACGTTCTTCGGGGCGAAAACTCTCAAGGATCTT
ACCGCTGTTGAGATCCAGTTCGATGTAACCCACTCGTGCACCCAACTGATCTTCAGCATCTTTTACTTTCACCAGCGTTTCTGGGTGAGCAAAAACAGGAAGGCAAAATGCCGCAAAAAAGGGAATA
AGGGCGACACGGAAATGTTGAATACTCATACTCTTCCTTTTTCAATATTATTGAAGCATTTATCAGGGTTATTGTCTCATGAGCGGATACATATTTGAATGTATTTAGAAAAATAAACAAATAGGGGTT
CCGCGCACATTTCCCCGAAAAGTGCCACCTGACGTCTAAGAAACCATTATTATCATGACATTAACCTATAAAAATAGGCGTATCACGAGGCCCTTTCGTCTCGCGCGTTTCGGTGATGACGGTGAAA
ACCTCTGACACATGCAGCTCCCGGAGACGGTCACAGCTTGTCTGTAAGCGGATGCCGGGAGCAGACAAGCCCGTCAGGGCGCGTCAGCGGGTGTTGGCGGGTGTCGGGGCTGGCTTAACTATG
CGGCATCAGAGCAGATTGTACTGAGAGTGCACCATAGATCAACGACATTACTATATATATAATATAGGAAGCATTTAATAGAACAGCATCGTAATATATGTGTACTTTGCAGTTATGACGCCAGATGG
CAGTAGTGGAAGATATTCTTTATTGAAAAATAGCTTGTCACCTTACGTACAATCTTGATCCGGAGCTTTTCTTTTTTTGCCGATTAAGAATTAATTCGGTCGAAAAAAGAAAAGGAGAGGGCCAAGAG
GGAGGGCATTGGTGACTATTGAGCACGTGAGTATACGTGATTAAGCACACAAAGGCAGCTTGGAGTATGTCTGTTATTAATTTCACAGGTAGTTCTGGTCCATTGGTGAAAGTTTGCGGCTTGCAGA
GCACAGAGGCCGCAGAATGTGCTCTAGATTCCGATGCTGACTTGCTGGGTATTATATGTGTGCCCAATAGAAAGAGAACAATTGACCCGGTTATTGCAAGGAAAATTTCAAGTCTTGTAAAAGCATA
TAAAAATAGTTCAGGCACTCCGAAATACTTGGTTGGCGTGTTTCGTAATCAACCTAAGGAGGATGTTTTGGCTCTGGTCAATGATTACGGCATTGATATCGTCCAACTGCATGGAGATGAGTCGTGG
CAAGAATACCAAGAGTTCCTCGGTTTGCCAGTTATTAAAAGACTCGTATTTCCAAAAGACTGCAACATACTACTCAGTGCAGCTTCACAGAAACCTCATTCGTTTATTCCCTTGTTTGATTCAGAAGCA
GGTGGGACAGGTGAACTTTTGGATTGGAACTCGATTTCTGACTGGGTTGGAAGGCAAGAGAGCCCCGAAAGCTTACATTTTATGTTAGCTGGTGGACTGACGCCAGAAAATGTTGGTGATGCGCTT
AGATTAAATGGCGTTATTGGTGTTGATGTAAGCGGAGGTGTGGAGACAAATGGTGTAAAAGACTCTAACAAAATAGCAAATTTCGTCAAAAATGCTAAGAAATAGGTTATTACTGAGTAGTATTTATTT
AAGTATTGTTTGTGCACTTGCCGATCTATGCGGTGTGAAATACCGCACAGATGCGTAAGGAGAAAATACCGCATCAGGAAATTGTAAGCGTTAATATTTTGTTAAAATTCGCGTTAAATTTTTGTTAAA
TCAGCTCATTTTTTAACCAATAGGCCGAAATCGGCAAAATCCCTTATAAATCAAAAGAATAGACCGAGATAGGGTTGAGTGTTGTTCCAGTTTGGAACAAGAGTCCACTATTAAAGAACGTGGACTCC
AACGTCAAAGGGCGAAAAACCGTCTATCAGGGCGATGGCCCACTACGTGAACCATCACCCTAATCAAGTTTTTTGGGGTCGAGGTGCCGTAAAGCACTAAATCGGAACCCTAAAGGGAGCCCCCG
ATTTAGAGCTTGACGGGGAAAGCCGGCGAACGTGGCGAGAAAGGAAGGGAAGAAAGCGAAAGGAGCGGGCGCTAGGGCGCTGGCAAGTGTAGCGGTCACGCTGCGCGTAACCACCACACCCG
CCGCGCTTAATGCGCCGCTACAGGGCGCGTCCATTCGCCATTCAGGCTGCGCAACTGTTGGGAAGGGCGATCGGTGCGGGCCTCTTCGCTATTACGCCAGCTGGCGAAAGGGGGATGTGCTGC
AAGGCGATTAAGTTGGGTAACGCCAGGGTTTTCCCAGTCAC

Table S3. Protein expression and purification strategies, related to STAR Methods.

Protein	Expression organism	Pull down strategy	N-/C- tag	Elution	Chromatography columns
**ORC**	*S. cerevisiae*	calmodulin	CBP-Orc1	EGTA	HiLoad Superdex 16/600
**Cdc6**	*E. coli*	glutathione	GST-Cdc6	PreScission	Hydroxyapatite, dialysis
**MCM-Cdt1**	*S. cerevisiae*	calmodulin	CBP-Mcm3	EGTA	HiLoad Superdex 16/600
**DDK**	*S. cerevisiae*	calmodulin	CBP-Dbf4	EGTA	Superdex200
**Dpb11**	*S. cerevisiae*	3xFlag	Dpb11-3xFlag	Flag peptide	Superdex200
**Pol ε**	*S. cerevisiae*	calmodulin	Dpb4-CBP	EGTA	Heparin, Superdex200
**exo- Polε**	*S. cerevisiae*	calmodulin	Dpb4-CBP	EGTA	Heparin, Superdex200
**ΔCAT Polε**	*S. cerevisiae*	calmodulin	Dpb4-CBP	EGTA	MonoQ, Superdex200
**GINS**	*E. coli*	Ni-NTA	6His-GINS	Imidazole	MonoQ, Superdex200
**Cdc45**	*S. cerevisiae*	3xFlag	internal 2xFlag	Flag peptide	Superdex200
**CDK**	*S. cerevisiae*	calmodulin	CBP-Clb5	EGTA	MonoQ, Superdex200
**Sld3/Sld7**	*S. cerevisiae*	IgG sepharose	TCP-Sld3	TEV	NiNTA (remove TEV), Superdex200
**Sld2**	*E. coli*	glutathione	GST-Sld2	PreScission	SP FF, dialysis
**RPA**	*E. coli*	-	-	-	HiTrap® Blue High Performance, Bio-Scale Mini CHT Type I Cartridge, MonoQ, Superdex200
**Ctf4**	*S. cerevisiae*	calmodulin	CBP-Ctf4	EGTA	MonoQ, Superdex200
**TopI**	*S. cerevisiae*	calmodulin	CBP-TopI	EGTA	Superdex200
**TopII**	*S. cerevisiae*	calmodulin	TopII-CBP	EGTA	MonoQ, Superdex200
**Polδ**	*S. cerevisiae*	calmodulin	Pol32-CBP	EGTA	Heparin, Superdex200
**Rfc1-RFC**	*S. cerevisiae*	calmodulin	CBP-Rfc3	EGTA	SP FF, Superdex200
**PCNA**	*E. coli*	ammonium sulfate	-	-	Heparin/SP FF/DEAE, MonoQ, Superdex200
**Mrc1**	*S. cerevisiae*	3xFlag	Mrc1-3xFlag	Flag peptide	MonoQ, Dialysis
**Csm3/Tof 1**	*S. cerevisiae*	calmodulin	CBP-Csm3	TEV	Superdex200
**Polα**	*S. cerevisiae*	calmodulin	CBP-Pri1	EGTA	MonoQ, Superdex200
**Mcm10**	*E. coli*	Flag/NiNTA	His-Mcm10-Flag	Flag peptide/ Imidazole	NiNTA, Superdex200
**Fen1**	*S. cerevisiae*	3xFlag	Fen1-3xFlag	Flag peptide	Heparin, Dialysis
**Cdc9**	*S. cerevisiae*	2xFlag	Cdc9-2xFlag	Flag peptide	MonoQ, Dialysis
**FACT**	*S. cerevisiae*	3xFlag	3xFlag-Pob3	Flag peptide	-
**Nhp6**	*S. cerevisiae*	untagged	-	-	Negative TCA precipitation, MonoS
**Rad53**	*E. coli*	NiNTA	Rad53-6His	Imidazole	Superdex200
**MRX**	*S. cerevisiae*	3xFlag	Xrs2-3xFlag	Flag peptide	Heparin, Superose6
**Elg1-RFC**	*S. cerevisiae*	calmodulin	CBP-Rfc3	EGTA	SP FF, Superdex200
